# Development of Novel Pt(IV)-Carbohydrate Derivatives as Targeted Anticancer Agents against Osteosarcoma

**DOI:** 10.3390/ijms24076028

**Published:** 2023-03-23

**Authors:** Eoin Moynihan, Silvia Panseri, Giada Bassi, Arianna Rossi, Elisabetta Campodoni, Eithne Dempsey, Monica Montesi, Trinidad Velasco-Torrijos, Diego Montagner

**Affiliations:** 1 Department of Chemistry, Maynooth University, W23 F2H6 Maynooth, Ireland; 2 Institute of Science, Technology and Sustainability for Ceramics, National Research Council (CNR), 48018 Faenza, Italy; 3 Department of Neuroscience, Imaging and Clinical Sciences, University of Studies “G. D’Annunzio”, 66100 Chieti, Italy; 4 Department of Chemical, Biological, Pharmaceutical and Environmental Sciences, University of Messina, 98166 Messina, Italy; 5 Kathleen Londsdale Institute for Human Health Research, Maynooth University, W23 F2H6 Maynooth, Ireland

**Keywords:** Pt(IV) pro-drugs, cisplatin, carbohydrate, selective targeting, osteosarcoma, healthy cell, click chemistry

## Abstract

Despite the enormous importance of cisplatin as a chemotherapeutic agent, its application is impacted by dose-limiting side effects and lack of selectivity for cancer cells. Researchers can overcome these issues by taking advantage of the pro-drug nature of the platinum(IV) oxidation state, and by modifying the coordination sphere of the metal centre with specific vectors whose receptors are overexpressed in tumour cell membranes (e.g., carbohydrates). In this paper we report the synthesis of four novel carbohydrate-modified Pt(IV) pro-drugs, based on the cisplatin scaffold, and their biological activity against osteosarcoma (OS), a malignant tumour which is most common in adolescents and young adults. The carbohydrate-targeting vectors and Pt scaffold are linked using copper-catalysed azide–alkyne cycloaddition (CuAAC) chemistry, which is synonymous with mild and robust reaction conditions. The novel complexes are characterised using multinuclear 1D-2D NMR (^1^H, ^13^C and ^195^Pt), IR, HR-MS, Elem. Analyses, and CV. Cytotoxicity on 2D and 3D and cell morphology studies on OS cell lines, as well as non-cancerous human foetal osteoblasts (hFOBs), are discussed.

## 1. Introduction

**Figure 1:**
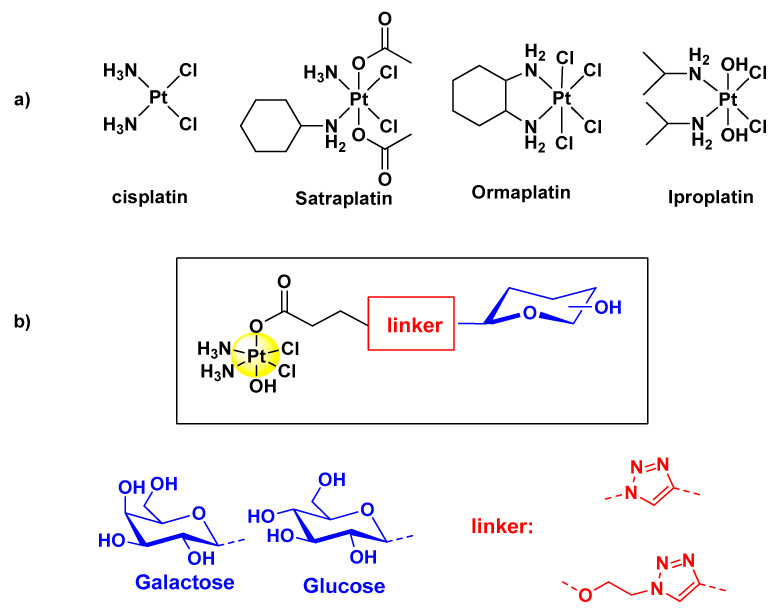
(**a**) Structures of cisplatin (*cis*-[(NH_3_)_2_PtCl_2_]) and of the three most promising Pt(IV) complexes, satraplatin (*cis,trans,cis-*[PtCl_2_(OAc)_2_(NH_3_)-cyclohexylamine]), ormaplatin ([PtCl_4_(cyclohexane-1,2-diamine)]), and iproplatin (*cis,trans,cis*-[PtCl_2_(OH)_2_(isopropylamine)_2_]). (**b**) General structure for the novel Pt(IV) complexes based on the cisplatin scaffold and functionalised with glucose and galactose.

Cisplatin has been one of the most commonly used Platinum(II) chemotherapeutic drugs since its approval for clinical use over 40 years ago. However, issues associated with acquired or innate resistance, as well as dose-limiting side effects, have stymied its therapeutic potential [1]. Second and third generation Pt(II)-based chemotherapeutics, carboplatin and oxaliplatin, are used to combat resistance, by lowering hydration rates in the case of carboplatin, and by a different mechanism of action that avoids cross-resistance with cisplatin in the case of oxaliplatin [2]. While these drugs have helped to alleviate cisplatin resistance in a variety of cancers, the main problem is still a lack of targeted therapy to reduce the severe side effects associated with platinum-based drugs [3]. Platinum(IV) complexes have the potential to overcome some of the drawbacks of currently available Pt(II) drugs. They are more kinetically inert in comparison to their Pt(II) counterparts, which results in lower toxic side effects for the patient. These complexes are referred to as pro-drugs as they must be reduced from the inactive Pt(IV) complex to their active Pt(II) species by the reducing environment within cancer cells, which leads to a more targeted approach of drug delivery to the cancer cells [4]. The most promising Pt(IV) drugs are satraplatin [5,6], ormaplatin [7,8] and iproplatin [9,10] but, despite promising results, none to date have been approved for widespread use in cancer treatments (Figure 1a) [3]. Promising strategies for imparting selectivity to Pt(IV) pro-drugs is the use of tumour-targeting ligands. Carbohydrates, such as glucose and galactose, can be used to exploit the Warburg effect [11], because cancer cells metabolise glucose differently from healthy cells. Rapid proliferation of cancer cells leads to the imbalance in the intake of oxygen and results in hypoxia, which drives cancer cells to choose anaerobic glycolysis to produce energy. This method of energy production is inefficient and leads to the overexpression of glucose transporters (GLUTs) on the cell membrane [12]. Several glyco-modified Pt(IV) complexes have been reported in literature, mainly from the group of Wang and co-workers [13,14,15,16,17], summarised excellently by Kenny and Marmion [3]. The most notable results were obtained when the Pt scaffold was cisplatin rather than carboplatin or oxalilplatin [18]. It was also determined that mono-functionalisation was more effective than bis-functionalised complexes [14]. Platinum-based complexes are extensively used to treat osteosarcoma (OS), the most common malignant primary bone tumour which affects adolescents and young adults, with a second spike of incidence in those over 50, and which tends to metastasize and invade para-carcinoma tissues [19]. Currently, treatment options incorporate surgery and combination chemotherapy, which cures 70% of patients. However, for patients with metastatic or relapsed OS, the modes of treatment have remained unchanged for over 30 years and result in a significantly lower survival rate among patients [20]. This reduction in survivability is partially because cisplatin is a standard drug in the treatment of OS and has limited effectiveness, stemming from chemoresistance and a lack of drug selectivity. This lack of selectivity and a need to overcome cisplatin resistance has led many to develop novel compounds to target specific molecular alterations in tumours, with the objective of restoring chemosensitivity [21]. Recently, our group reported the synthesis and biological evaluation against osteosarcoma of four acetylated glyco-modified Pt(IV) complexes, but, despite the promising results, the role of the sugars was not completely clarified [22]. The glycosylated complexes proved to have an enhanced cellular uptake, but this was probably due to the higher lipophilicity caused by the acetylated groups, rather than due to recognition by GLUTs. Indeed, these protecting groups are prone to enzymatic hydrolysis in the body, ultimately making them pro-drugs themselves [23]. The crystal structures of various GLUTs have enabled researchers to focus their work on the optimal presentation of their carbohydrates to target these transporters [24,25], and free sugars are employed as they more closely mimic the structure of the compounds recognised by various GLUTs that are overexpressed on cancer cell membranes. To try to clarify this aspect, we decided to synthesise four glyco-derivatives (two with glucose and two with galactose) for the previously reported complexes with the deprotected (free) sugars and evaluate their biological activity against two osteosarcoma cell lines and a non-cancerous human foetal osteoblast (hFOB), (Figure 1b). In this work we functionalise our carbohydrate-targeting vector in β configuration at the anomeric position, since an important factor for maintaining recognition is to conserve the sugars’ ability to form hydrogen bonds with the GLUT binding site through the atom bound to the anomeric position acting as a H-bond acceptor [26]. To maintain this H-bonding ability, we take advantage of the copper-catalysed azide–alkyne cycloaddition (CuAAC) reaction due to its versatile nature and mild reaction conditions for the conjugation of our carbohydrates to the platinum core. The resulting 1,2,3-Triazole is recognised as a strong bio-isostere for many functionalities, including amide bonds, and has H-bonding acceptor properties [27]. Additionally, we wanted to investigate the use of both an anomeric *O*-ethylene linker (which resembles naturally occurring glycosides) and *N*-triazolyl glycomimetics, to analyse if there is a significant difference in the H-bond acceptor ability, and subsequent activity, of either group (Figure 1b).

The anticancer activity of the glyco-derivatives reported here have been investigated in both standard 2D and in 3D scaffold-based OS in vitro models, using two different OS cell lines (MG63 and SAOS-2) and osteoblast cells line (hFOBs) as healthy cells to compare the effectiveness against a standard of cisplatin.

## 2. Results and Discussion

### 2.1. Synthesis and Characterisation

**Scheme 1:**
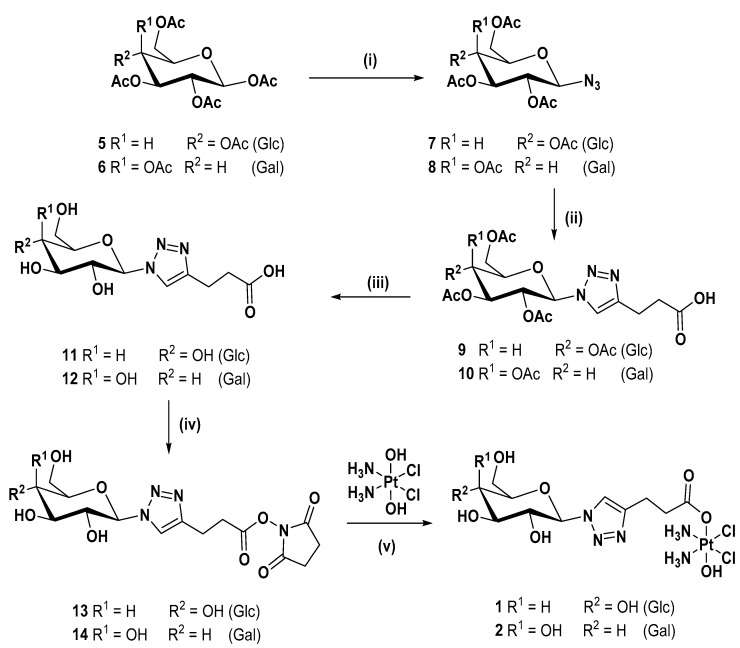
Synthetic route for the complexes **1** and **2**: (i) TMSN_3_, SnCl_4_, DCM, rt, 16 h, 91% (**7**), 89% (**8**); (ii) 4-pentynoic acid, CuSO_4_, sodium ascorbate, t-BuOH, THF, H_2_O, rt, 16 h, 64% (**9**), 74% (**10**); (iii) TEA, MeOH, H_2_O, 45 °C, 16 h, 95% (**11**), 97% (**12**); (iv) TSTU, TEA, DMF, rt, 20 min, 68% (**13**), 68% (**14**); (v) DMSO, 40 °C, 48 h, 59% (**1**), 35% (**2**).

**Scheme 2:**
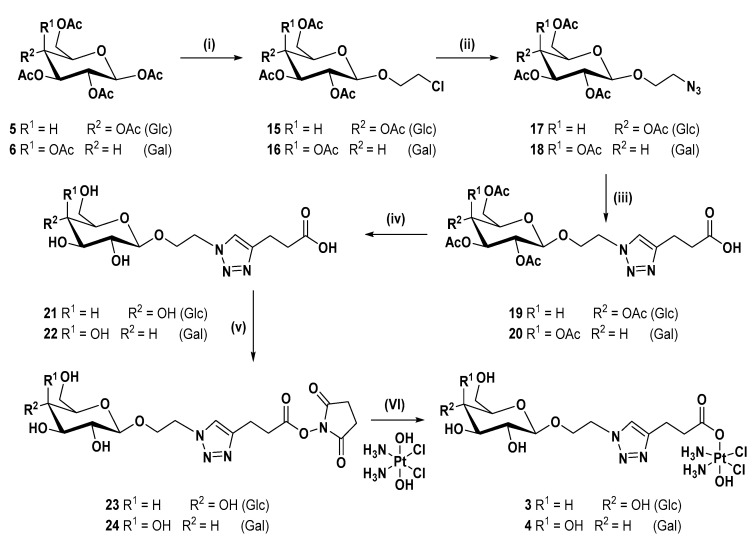
Synthetic route for complexes **3** and **4**: (i) 2-Chloroethanol, BF_3_.OEt_2_, 3 Å MS, 0 °C to rt, DCM, 16 h, 39% (**15**), 40% (**16**); (ii) NaN_3_, DMF, 80 °C, 16 h, 59% (**17**), 57% (**18**); (iii) CuSO_4_, sodium ascorbate, t-BuOH, THF, H_2_O, rt, 16 h, 44% (**19**), 47% (**20**); (iv) TEA, MeOH, H_2_O, 45 °C, 16 h, 86% (**21**), 96% (**22**); (v) TSTU, TEA, DMF, 20 min, rt, 60% (**23**), 75% (**24**); (vi) DMSO, 40 °C, 48 h, 47% (**3**), 61% (**4**).

As described in the introduction, the aim of this work is to synthesise Pt(IV) anticancer pro-drugs based on a cisplatin scaffold modified with free sugars (glucose and galactose) that could act as delivery vectors to enhance selectivity for cancer cells, exploiting the Warburg effect. With respect to our recent work [22], the sugars here are de-acetylated to obtain a more reliable recognition by the GLUT receptors overexpressed on the cancer cell membrane, as discussed in the introduction. The sugar moieties are connected to the platinum centre via a triazolyl linker exploiting the versatility and mild conditions of click chemistry copper azide–alkyne cycloaddition (CuAAC) reactions, producing four novel complexes. The synthesis of the novel glycosylated Pt(IV) complexes proved to be rather challenging; in fact, the direct deprotection of the corresponding acetylated glycosylated precursor complexes, recently reported by us [22], did not produce the expected results. The use of standard deprotection methods (i.e., NEt_3_ in MeOH) resulted in the decomposition of the complexes, forming a series of impurities and a complex mixture which was very difficult to purify and isolate. After several attempts and modifications, the final complexes were successfully synthesised, as shown in Scheme 1 and Scheme 2.

Scheme 1 represents the synthetic mechanism route to produce the N-glycoside (glucose and galactose) derivatives with shorter chains. The synthesis of compounds **5**–**10** has been reported by us previously [22]. To summarise briefly, the per-acetylated glucose **5** and galactose **6** are transformed to the corresponding β-azides, **7** and **8**, through reaction with azidotrimethylsilane and tin tetrachloride. Following this, **7** and **8** are reacted with 4-pentynoic acid, using CuAAC conditions at room temperature, to produce the carboxylic acids **9** and **10**. At this point, the acetylated sugars are deprotected with triethylamine in a mixture of MeOH/H_2_O at 45 °C to yield carboxylic acids **11** and **12**. Many standard coupling reagents and conditions were screened in order to identify a suitable procedure that would allow for the synthesis of activated N-hydroxysuccinimide (NHS) esters **13** and **14** in the presence of the hydroxyl groups in the deprotected sugars. Initial attempts for the classic activation with NHS using EDCI or TBTU did not yield the desired products. Finally, optimised reaction conditions with TSTU (N,N,N′,N′-Tetramethyl-O-(N-succinimidyl)uronium tetrafluoroborate) produced the activated NHS-esters **13** and **14** with good yield and purity. The advantage of using TSTU is that the reaction does not require the use of an additional coupling reagent and vastly reduces reaction time to a mere 20 min. The final complexes **1** and **2** were obtained through the dropwise addition of **13** and **14** to oxoplatin in DMSO over 2 days. The final complexes were characterised using ^1^H, ^13^C, ^195^Pt-NMR, mass spectroscopy and cyclic voltammetry, and the purity was assessed with El. Anal. And HPLC (See Experimental Section and Supporting Information). 

Complexes **3** and **4,** with a O-ethylene linker, were synthesised according to Scheme 2, in a manner similar to the synthesis of the N-triazolyl derivatives **1** and **2,** described earlier. Compounds **19** and **20**, recently reported by us [22], were hydrolysed under mild basic conditions to afford deacetylated carboxylic acids **21** and **22**. The same challenges described above remained for the synthesis of free sugar complexes **3** and **4** and consequently, the same strategies were applied. The successful formation of the NHS active esters **23** and **24** was achieved once again with the use of TSTU, and the final complexes **3** and **4** were obtained through the reaction of the activated deprotected sugar moieties **23** and **24** with oxoplatin in DMSO. These complexes were characterised using the same techniques as for complexes **1** and **2**. All the complexes show the typical features of Pt(IV) species based on a cisplatin scaffold, with a broad triplet around 6 ppm assigned to the NH_3_ groups in the ^1^H-NMR spectrum, the ^195^Pt signal around 1600 ppm, typical of Pt in the oxidation state +4 and the typical Pt isotopic pattern in the mass spectra (Supplementary Materials Figures S1–S16).

**Figure 2:**
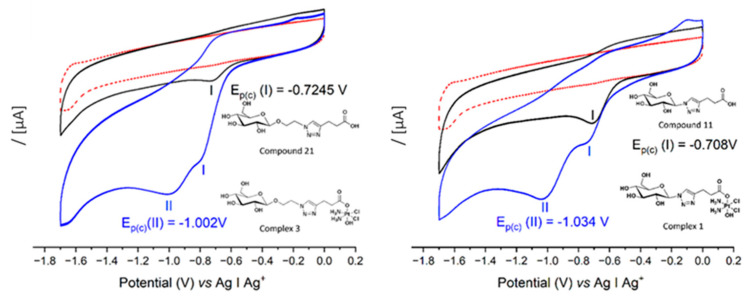
The cyclic voltammograms of the complexes **1** and **3** (blue), with evidence of irreversible cathodic processes I and II and of the axial ligands **11** and **21** (black), showing process I only, with background supporting electrolyte (red). All experiments were performed at the same concentration ([compound] = 2 mM) in deuterated 0.1 M DMSO-[*n*-Bu_4_N][PF_6_] at a scan rate of 0.1 Vs^−1^.

The stability in physiological conditions of complexes **1** and **3**, taken as examples for the different substituents at the anomeric position) was analysed over a period of 96 h via ^1^H, ^13^C and ^195^Pt-NMR in a solution of DMSO containing PBS buffer (pH = 6.8) and minimum decomposition was observed (Figure S17a–f in Supplementary Materials). The redox behaviour of complexes **1** and **3** (cathodic processes $E_{p}^{c}\;\left(I\right)$, $E_{p}^{c}\;\left(\mathrm{II}\right)$, Figure 2) and of the corresponding axial glycol-ligands **11** and **21** ($E_{p}^{c}\;\left(I\right)$, Figure 2) was studied via Cyclic Voltammetry with E^0^_Pt(IV)/Pt(II)_ = −1.034 V and −1.002 V for **1** and **3,** respectively $E_{p}^{c}\;\left(\mathrm{II}\right)$. The electrochemical reduction was irreversible, in line with previous reported Pt(IV) species based on cisplatin [28], and no major difference was observed according to the type of linker used to connect the carbohydrate moiety and the Pt centre (Figure 2, Figures S18 and S19 in Supplementary Materials). 

**Figure 3:**
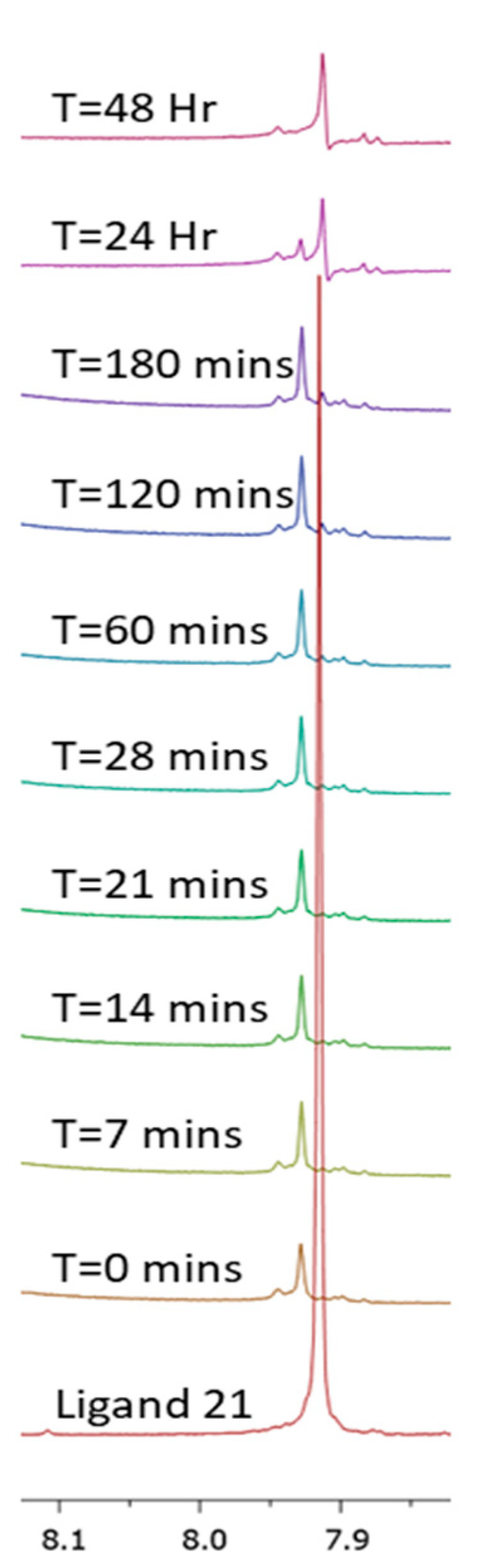
Reduction of complex **3** by a 10-fold excess of ascorbic acid. ^1^H NMR spectra over 48 h in DMSO.

For a confirmation of the activation through intracellular reduction that occurs in Pt(IV) species, the behaviour of the Pt(IV) pro-drugs was studied by means of the addition of an excess of ascorbic acid into a solution of complex **3** [29]. The reduction process was followed by ^1^H-NMR with complete release of the carbohydrate axial ligand after 48 h, as observed by the disappearance of the triazole proton at δ = 7.93 of complex **3** and the concomitant formation of the corresponding peak at δ = 7.91 of the free carboxylic acid **21** (Figure 3 and Figure S20 in Supplementary Materials).

### 2.2. Biological Evaluation

The anticancer activity of the four complexes were tested in vitro in 2D and 3D against two osteosarcoma cancer cell lines (SAOS and MG63) and also in 2D against a healthy osteoblast cell line (hFOBs), to evaluate if the strategic use of glucose and galactose as vectors is enhancing the selectivity.

**Figure 4:**
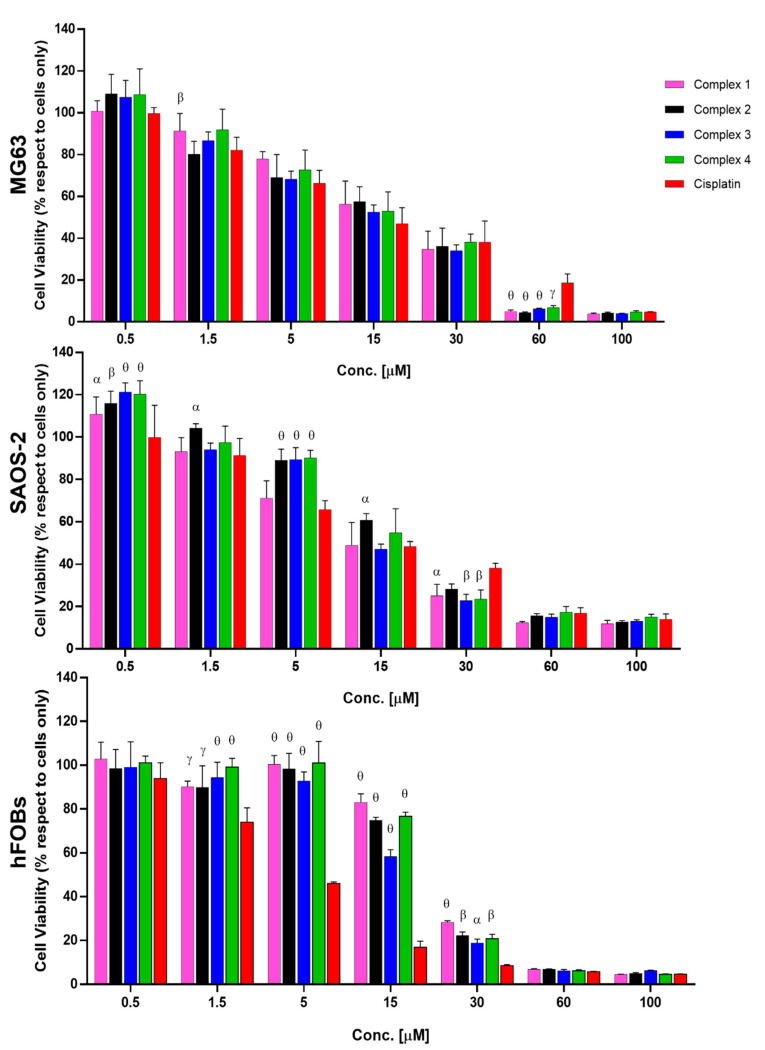
2D drug screening of complexes **1–4** by MTT assay. Cell viability evaluation after 72 h incubation with complexes. Data are reported in the graph as percentage (%) mean ± standard error of the mean. Statistical analyses with respect to cisplatin are reported in the graph, *p* value is α ≤ 0.05, β ≤ 0.01, γ ≤ 0.001 and θ ≤ 0.0001.

The evaluation of cell viability demonstrated a dose-dependent cancer cell toxicity exerted by all the drugs (Figure 4). Although no significant differences in the IC_50_ (Table 1, Figures S21–S23 in Supplementary Materials) were detected in the four complexes compared to cisplatin, at high concentrations the complexes showed better performance with respect to cisplatin. In MG63 cells, the mean effect of the complexes **1**–**4** showed a ~3-fold reduction of the cell viability with respect to cisplatin, and ~1.5-fold reduction for SAOS-2 (Figure 4). The higher anticancer activity of all the complexes **1**–**4,** with respect to cisplatin, was statistically significant at 30 µM for SAOS-2 and 60 µM for MG63. The different cellular behaviour between the cell lines can be attributed to the intrinsic biological differences of the two osteosarcoma cell lines [30].

Most importantly, in the healthy cell hFOBs, the cisplatin began to be extremely toxic at very low concentration, 1.5 µM (*p* ≤ 0.001 with respect to cells only), while complexes **1**–**4** showed a cytotoxic effect at much higher concentrations, ≥ 15 µM (complex 1 *p* ≤ 0.05; 2 *p* ≤ 0.001; 3 *p* ≤ 0.0001; 4 *p* ≤ 0.001 respect to cells only). The evaluation of the IC_50_ values confirmed the cell viability results; in fact, in the hFOBs, the IC_50_ of the cisplatin (i.e., 4.1 µM) was far lower compared to the IC_50_ values of complexes **1**–**4,** which were > 16 µM (Table 1). Moreover, it was shown that the toxic effect of the complexes is statistically significantly lower, with respect to the effect of cisplatin, at 1.5 µM, 5 µM, 15 µM, and 30 µM (Figure 4). The cell behaviours observed in the cell viability assay were also confirmed using morphological analysis, where a reduction in cell density directly related to the increase in drug concentration was detected (Figure S24 in Supplementary Materials).

The results demonstrated that complexes **1**–**4** all have selectivity for these cancer cells and a lower toxic effect on healthy hFOBs. We believe that this selectivity could be ascribable to the overexpression of the GLUT family members, specifically GLUT-1, observed in cancer cells leading to an increased glycolytic activity [31,32]. The higher toxicity of all complexes with respect to the cisplatin, observed in both MG63 and SAOS-2 at 30 µM and 60 µM, strengthens the hypothesis of the role of the GLUT receptor overexpression in the uptake, induced by the presence of sugar moieties connected to the drug, and consequently in the increased anticancer effect. 

**Figure 5:**
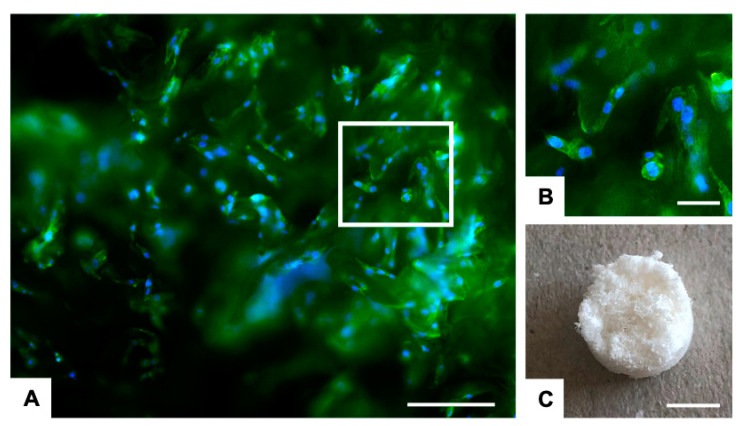
3D scaffold-based osteosarcoma model (3D OS model). (**A**) Analysis of cell morphology of 3D MG63 culture on bone-mimetic scaffold 48 h after seeding. F-Actin filaments in green (FITC) and cell nuclei in blue (DAPI); scale bars 200 µm. (**B**) Image enlargement (white square) of the cell morphology details; scale bars 50 µm. (**C**) Representative image of the 3D bone-mimetic scaffold; scale bars 4 mm.

In order to confirm these promising results, a proof of concept in vitro was performed using more relevant cancer models. In fact, it is well known that the use of conventional 2D approaches show some limitations because they fail to mimic a real tumour’s complexity, leading to a low predictivity of preclinical results [33,34]. To overcome this limitation, and to strengthen the results obtained in the previous 2D study, an MG63 cell line was cultured on a 3D scaffold that mimics the feature of the bone extracellular matrix (Figure 5C) from a physical, chemical, and nanostructural point of view, as previously demonstrated [35,36].

After the seeding of the MG63 cell line, the cells were able to interact and colonise the scaffold, providing a more mimetic 3D scaffold-based osteosarcoma model (3D OS model) which was then used to test the effect of the proposed drugs. As shown in Figure 5A,B, MG63 cells easily adhered to the nanostructure of the biomimetic scaffolds and, 48 h after seeding, the scaffolds were nearly completely covered by the cells, exhibiting their characteristic morphology and a high level of cell/material interactions. 

It is well known that the behaviour of the cells cultured in 3D condition is different respect to the standard 2D model [37]. For this reason, before testing the complexes in the 3D OS mode, a preliminary evaluation of the cisplatin effective concentration was performed by comparing the toxicity of cisplatin 15 µM in 2D and in the 3D OS model. As shown in Figure S25 in Supplementary Materials, 15 µM of cisplatin did not compromise the cell viability in 3D OS model, though it significantly reduced the viable cells grown in 2D standard culture conditions (*p*-value ≤ 0.0001).

**Figure 6:**
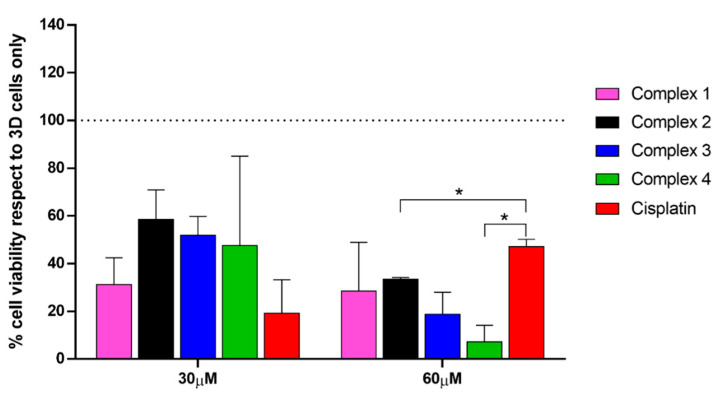
Anti-cancerous effect of **1–4** complexes on 3D OS model**.** Cell viability evaluation after 72 h of incubation with complexes using MTT assay. Data are reported in the graph as percentage (%) mean ± standard error of the mean. Statistical analyses with respect to cisplatin are reported in the graph, * *p* value ≤ 0.05.

Based on these results, the complexes **1**–**4** and the cisplatin were tested at 30 and 60 µM in the 3D tumour model. At 60 µM, all the complexes showed higher toxic effect respect to the cisplatin, and these differences are statistically significant for complexes **2** and **4** with *p* value ≤ 0.05 (Figure 6). Morphological analysis (Figure S26 in Supplementary Materials) confirms the cell viability results. 

This proof of concept highlights the cancer cells selectivity of the complexes for the considered OS cells, compared to the cisplatin action, and reinforces the data obtained in the 2D in vitro study, demonstrating better performances of the complexes **1**–**4** as anticancer drugs. 

## 3. Materials and Methods

### 3.1. General Methods

All reagents and reactants were purchased from commercial sources. The two sources used were Sigma-Aldrich (St.Louis, MO, USA) and Fluorochem (Graphite way, Hadfield, UK). All solvents were used without further purification. Cisplatin and oxoplatin were synthesised as previously reported [38,39]. Compounds **7–10** and **15–20** were synthesised as reported by us [22].

The elemental analysis studies (carbon, hydrogen, and nitrogen) were performed by means of a PerkinElmer 2400 series II analyser (Waltham, MA, USA). HR mass spectra were recorded with a Waters LCT Premier XE Spectrometer (Milford, MA, USA). NMR: ^1^H, ^13^C and ^195^Pt NMR spectra were obtained in a solution of D_2_O or DMSO-*d*_6_ at 300 K, in 5 mm sample tubes, with a premium shielded Agilent Varian 500 MHz (operating at 500.13, 125.75, and 107.49 MHz, respectively)(Santa Clara, CA, USA). The ^1^H and ^13^C chemical shift was referenced to the residual impurity of the solvent. The external reference was Na_2_PtCl_4_ in D_2_O (adjusted to δ = −1628 ppm from Na_2_PtCl_6_) for ^195^Pt. The stability was followed using high-performance liquid chromatography (HPLC) with a Phenomenex Luna C18 (5 μM, 100 Å, 250 mm × 4.60 mm i.d.) column (Torrance, CA, USA) at room temperature at a flow rate of 1.0 mL/min with 254 nm UV detection. Mobile phase containing 80:20 acetonitrile (0.1% trifluoroacetic acid): water (0.1% trifluoroacetic acid): the complexes were dissolved in DMF (0.5 mL) and diluted to a final concentration of 0.5 mM, using acetonitrile and water solution (1/1) and 2 mM 4-(2-hydroxyethyl)piperazine-1-ethanesulfonic acid (HEPES) buffer (pH 6.8). Infrared (IR) spectra were precisely recorded in the region 4000–400 cm^−1^ on a Perkin Elmer spectrum 100 FT/IR spectrometer (Shelton, CT, USA). The solid samples were run using ATR. An extensive biological evaluation of the activity of all the compounds was performed in human osteosarcoma cell line in vitro models as reported below.

### 3.2. Cyclic Voltammetry

Measurements were made using a Solartron SI2187 Electrochemical Potentiostat/Galvanostat (Berwyn, PA, USA) and a CHI Instruments 1200 Potentiostat (Austin, TX, USA). Non-aqueous voltammetry of 2 mM of each of **11**, **21**, **1** and **3** was carried out at a glassy carbon working electrode (0.07 cm^2^) in a three-electrode configuration with Pt wire counter and non-aqueous Ag|Ag^+^ reference electrode in 0.1 M tetrabutylammonium hexafluorophosphate (TBAPF_6_) supporting electrolyte in DMSO as solvent. The working electrodes were prepared by polishing with 1 μm microcrystalline diamond suspension on a micro-cloth, followed by rinsing in deionised water. Voltammograms were generated over the range 0.2 to −1.7 V vs. Ag/Ag^+^ in a deaerated solution (N_2_ bubbling 10 min), with a cathodic scan direction at 100 mV∙s^−1^ in all cases.

### 3.3. Stability and Reduction Studies

The stability was analysed via ^1^H, ^13^C and ^195^Pt NMR. Complexes **1** and **3** (taken as examples for the different substituents at the anomeric position) were dissolved in a 1/1 mixture of DMSO and PBS (pH = 6.8), and the spectra were collected after 0 and 96 h. The reduction was followed by ^1^H-NMR. Complex 3 (5 mg, 7.53 µmol) was dissolved in 500 µL of DMSO-*d_6_* and ascorbic acid (13 mg, 10 Equiv.) was added. ^1^H NMR spectrum was recorded every 7 min for 30 min, then every hour until 24 h and then after 48 h.

### 3.4. In Vitro Biological Evaluation 

In vitro tests of the four Pt(IV)-Carbohydrate derivatives complexes (**1**–**4**) were performed to evaluate their biological activity towards two osteosarcoma cell lines (MG63 and SAOS-2) and a non-cancerous cell line of human foetal osteoblasts (hFOBs), compared to cisplatin. The drugs were reconstituted in dimethyl sulfoxide (DMSO) at 1 mg/mL concentration, before being diluted in culture media at the required concentration. A 2D in vitro screening of all the drugs was performed at 72 h in a wide concentration range (0.5; 1.5, 5, 15, 30, 60 and 100 µM) on the three different cell lines in terms of cell viability, and the IC_50_ was calculated. The cell morphology in the presence of the complexes was evaluated at 15 and 30 µM concentrations, for MG63 and SAOS-2 and hFOBs cells, respectively, according to the IC_50_ at 72 h. Moreover, as more predictive in vitro cell culture systems, 3D tumour scaffold-based models of osteosarcoma were developed, and the anticancer activity of cisplatin and complexes **1**–**4** on MG63 cells, in terms of cell viability and morphology, was investigated at 30 and 60 µM concentrations after 72 h of culture. For the models, a composite hydroxyapatite-based scaffold (MgHA/Coll), as a bone-like matrix, was used in combination with MG63 cells. For both the 2D and 3D in vitro cell cultures, cisplatin was used as a control group, and cells only were used as a negative control. 

*Cell culture.* Human Osteosarcoma cell lines MG63 (ATCC^®^ CRL1427™), SAOS-2 (ATCC^®^ HTB-85™), and Human Foetal Osteoblasts (hFOBs 1.19) (ATCC^®^ CRL-11372™) were purchased from American Type Culture Collection (ATCC) and used for this study. The MG63 cell line was cultured in DMEM F12-GlutaMAX™ Modified Medium (Gibco), supplemented with 10% Foetal Bovine Serum (FBS) (Gibco) and 1% of penicillin/streptomycin mixture (pen/strep) (100 U/mL–100 µg/mL, Gibco). The SAOS-2 cell line was cultured in McCoy’s 5A Modified Medium (Gibco), supplemented with 15% and 10% FBS, respectively, and 1% pen/strep. The hFOBs cell line was cultured in DMEM F12 no phenol red, with L-glutamine supplemented with 10% FBS and 0.3 mg/mL Geneticin (G418, Gibco). Cells were kept in an incubator at 37 °C under controlled humidity and 5% CO_2_ atmosphere conditions. Cells were detached from culture flasks via trypsinization and centrifuged. The cell number and viability were determined using the trypan blue dye exclusion test, and all cell handling procedures were performed under a laminar flow hood in sterility conditions. 

*Synthesis of bone-mimetic scaffolds.* The Mg-doped hydroxyapatite collagen composite scaffolds were designed and synthesised at ISSMC of CNR of Italy [35]. In brief, 150 g of collagen gel (1 wt%, Opocrin SpA, MO, Italy) was diluted into a phosphoric acid solution (2.4 g in 500 mL; H_3_PO_4_, 85 wt.%, Sigma-Aldrich) at room temperature to obtain an acidic aqueous homogenous suspension. Separately, a basic aqueous suspension was obtained by mixing 2.7 g of calcium hydroxide (Ca(OH)_2_, 95 wt.%, Sigma) and 0.35 g of magnesium chloride (MgCl_2_·6H_2_O, 99 wt.%, Sigma) in 500 mL of milli-Q water at room temperature to obtain a basic aqueous homogenous suspension. The acidic suspension was dripped into the basic one at 25 ± 2 °C under continuous stirring condition and maturated for 2 h. Later, the slurry solution was rinsed thrice in milli-Q water and filtered through metallic sieve (150 m) to exclude unreacted counter ions. The recovered slurry solution was cross-linked with 2 wt.% BDDGE (respect to Collagen) at 25 ± 2 °C for 24 h and at 4 °C for other 24 h. Later, the solution was rinsed thrice in milli-Q water to remove any residues and freeze-dried (−40 °C and +25 °C) for 48 h under 0.086 mbar vacuum conditions (LIO 3000 PLT, 5PASCAL, Italy). The obtained scaffolds (8 × 4 mm), named bone-mimetic scaffolds, were sterilised using 25 kGy γ-ray irradiation before use. 

*3D scaffold-based osteosarcoma models (3D OS model).* For the development of the in vitro 3D scaffold-based osteosarcoma model, bone-mimetic scaffolds were used as bone-like matrix in combination with MG63 cells. The scaffolds were conditioned in culture media for 24 h before the cell seeding. MG63 cell line was seeded with a density of 3.0 × 10^4^ per scaffold by dropping the cellular suspension on the material upper surface followed by 30 min pre-adhesion at 37 °C before cell media addition. The 3D OS model has been grown in the incubator under standard culture medium condition for 48 h to allow cell colonization of the scaffold, then the medium was changed, and the drugs were added. The in vitro 3D OS models were cultured in the presence of the drugs for 72 h at 37 °C under controlled humidity and 5% CO_2_ atmosphere conditions; the cells grown in 3D in standard condition, without the drugs, were used as control group (3D cells only). All cell handling procedures were performed under a laminar flow hood in sterility conditions. 

*MTT cell viability assay*. A quantitative analysis of cell viability was carried out by using MTT assay, following the manufacturer’s instructions. For the in vitro 2D cell cultures, all cell lines were seeded at a density of 5.0 × 10^3^ cells/well in 96 well-plates. For in vitro 3D cell cultures, see at the “3D scaffold-based models of osteosarcoma” paragraph of Materials and Methods section. The MTT reagent [3-(4,5-dimethylthiazol-2-yl)-2,5-diphenyltetrazolium bromide] (5 mg/mL) was dissolved in phosphate saline buffer 1X (PBS 1X). At 72 h, the cells were incubated with 10% media volume MTT solution for 2 h at 37 °C, 5% CO_2_ and controlled humidity conditions. The cell culture media was removed and substituted with DMSO (Merck) dissolving formazan crystals, derived from MTT conversion by metabolically active cells. For the 3D scaffold-based models of osteosarcoma, each scaffold was transferred into a 2 mL Eppendorf, and completely broken using pestles after DMSO addition. After 15 min of incubation under slight stirring conditions, the absorbance of formazan was read at 570 nm by using a Multiskan FC Microplate Photometer (Thermo Fisher Scientific, Waltham, MA, USA). The values of absorbance are directly proportional to the number of metabolic active cells in each well. One experiment was carried out, and a biological triplicate for each condition was performed. For the 3D tumour models, one biological experiment was performed, and two scaffolds for each condition were used. 

*Cell morphology evaluation*. For the in vitro 2D cell cultures, all cell lines were seeded at a density of 5.0 × 10^3^ cells/well in 96 well-plates, for the in vitro 3D cell cultures cells were treated as previously described. Both the 2D and 3D cell cultures were fixed in 4% buffered Paraformaldehyde (PFA) following the manufacturer’s instructions. The fixed samples were permeabilised in PBS 1X with 0.1% (*v*/*v*) Triton X-100 (Merck) for 5 min at room temperature and F-actin filaments were highlighted with Alexa Fluor 488 Phalloidin (Invitrogen) for 20 min at room temperature in the dark. DAPI (600 nM) counterstaining was performed for cell nuclei identification, following the manufacturer’s instructions. The images were acquired by using an Inverted Ti-E Fluorescent Microscope. 

*Statistical Analysis.* Statistical analysis was performed using GraphPad Prism Software (8.0.1 version). The results of the MTT assay of the in vitro 2D drug screening are reported in the graphs as mean percentage of cell viability, with respect to cells only ± standard deviation, and they were analysed using two-way analysis of variance (two-way ANOVA) and Dunnett’s multiple comparisons test. The MTT results were further analysed using one-way analysis of variance (one-way ANOVA) and Dunnett’s multiple comparisons test. IC_50_ values were calculated as Log(inhibitor) versus mean percentage of dead cells, with respect to cells only, and the obtained values are reported in the graphs ±95% confidence interval (CI) for each cell line. The MTT results for the 3D tumour engineered models of osteosarcoma were reported in the graph as percentage mean ± standard error of the mean, and they were analysed with two-way ANOVA and Dunnett’s multiple comparisons test. A further analysis was performed using the unpaired *t*-test on all drugs, with respect to cisplatin. 

### 3.5. Synthetic Procedures

Compounds **7–10** and **15–20** were synthesised as reported by us [22].

***N*-(*β*-D-glucopyranosyl-1,2,3-triazol-4-yl)-propanoic acid (11)**

**9** (0.612 g, 1.29 mmol) was suspended in a mixture of methanol (6 mL) and water (3 mL) and heated at 45 °C. NEt_3_ (0.2 mL) was added, and the reaction was allowed to stir overnight. The progress was checked using TLC (95:5 DCM:MeOH). The solvent was removed under vacuum and the residue was redissolved in H_2_O and stirred with Amberlite H^+^ resin for 40 min. The Amberlite was filtered, and the residue was dried via lyophilization and reacted in the following step without further purification (0.375 g, 1.04 mmol, 95%). R_f_ = 0.40 (DCM:MeOH:H_2_O 60:35:5). $[{\alpha ]}_{D}^{21.4}$+1.12 (c 0.825, MeOH). ^1^H NMR (500 MHz, D_2_O) δ 7.97 (s, 1H, triaz-C*H*), 5.67 (d, *J* = 9.2 Hz, 1H, H-1), 3.95 (t, *J* = 9.2 Hz, 1H, H-2), 3.87 (dd, *J* = 12.4, 2.0 Hz, 1H, H-6), 3.77–3.64 (m, 3H, H-3, H-6′, H-5), 3.62–3.56 (m, 1H, H-4), 2.96 (t, *J* = 7.4 Hz, 2H, triaz-C*H*_2_), 2.55 (t, *J* = 7.4 Hz, 2H, C*H*_2_CO). ^13^C NMR (125 MHz, D_2_O) δ 180.90 (*C*O), 147.92 (triaz-*C*), 122.15 (triaz-*C*H), 87.35 (C-1), 78.79 (C-5), 75.87 (C-3), 72.22 (C-2), 68.92 (C-4), 60.40 (C-6), 36.18 (*C*H_2_CO), 21.49 (triaz-*C*H_2_). IR (ATR) 3256.84, 2915.58, 1567.38, 1396.39, 1042.54, 898.47, 599.49 cm^−1^. HR-MS (+): *m*/*z* calcd for C_11_H_17_N_3_O_7_ + H^+^ [M + H]^+^ 304.1145, found 304.1140. HR-MS (+): *m*/*z* calcd for C_11_H_17_N_3_O_7_ + Na^+^ [M + Na]^+^ 326.0964, found 326.0958.

***N*-(*β*-D-glucopyranosyl-1,2,3-triazol-4-yl)-(3-oxopropyl-(oxy(2,5-dioxopyrrolidin-1-yl))) (13)**

**11** (0.2 g, 0.659 mmol) was added to a flask containing TSTU (0.218 g, 0.725 mmol, 1.1 equiv.) and placed under N_2_. Anhydrous DMF (10 mL) and anhydrous Triethylamine (0.101 mL, 0.725 mmol, 1.1 equiv.) were added. The reaction was stirred for 20 min, and reaction progress was monitored by TLC (60:35:5 DCM:MeOH:H_2_O). The solvent was removed in vacuo and the residue was washed with DCM. The precipitate was collected via centrifugation and dried, yielding a light pink powder (Yield 0.182 g, 0.454 mmol, 68%). R_f_ = 0.92 (DCM:MeOH:H_2_O 60:35:5). $[\alpha {]}_{D}^{23.5}$+ 3.75 (c 0.8, H_2_O). ^1^H NMR (500 MHz, DMSO) δ 8.11 (s, 1H, triaz-C*H*), 5.48 (d, *J* = 9.3 Hz, 1H, H-1), 5.32 (d, *J* = 6.0 Hz, 1H, O*H* of C-2), 5.26 (d, *J* = 4.6 Hz, 1H, O*H* of C-3), 5.14 (d, *J* = 5.4 Hz, 1H, O*H* of C-4), 4.62 (s, 1H,O*H* of C-6), 3.74–3.66 (m, 2H, H-2, H-6), 3.43 (d, *J* = 7.6 Hz, 2H, H-6′, H-5), 3.39 (dd, *J* = 8.9, 3.9 Hz, 1H, H-3), 3.21 (dt, *J* = 13.9, 7.0 Hz, 1H, H-4), 3.10–3.05 (m, 2H, C*H_2_*CO), 3.02–2.98 (m, 2H, triaz-C*H_2_*), 2.82 (s, 4H, C*H*_2_C*H*_2_-succ) ppm. ^13^C NMR (125 MHz, DMSO) δ 170.25 (*C*O succ ×2), 168.38 (*C*O), 144.37 (triaz-*C*), 121.40 (triaz-*C*H), 87.44 (C-1), 79.92 (C-5), 76.95 (C-3), 72.16 (C-2), 69.60 (C-4), 60.74 (C-6), 29.58 (*C*H_2_CO), 25.47 (*C*H_2_*C*H_2_-succ), 20.27 (triaz-*C*H_2_) ppm. IR (ATR) 3298.62, 2917.68, 1731.73, 1708.88, 1406.06, 1367.70, 1206.78, 1019.20, 818.67, 647.68 cm^−1^. HR-MS (+): *m*/*z* calcd for C_15_H_20_N_4_O_9_ + H^+^ [M + H]^+^ 401.1309, found 401.1300. HR-MS (+): *m*/*z* calcd for C_15_H_20_N_4_O_9_ + Na^+^ [M + Na]^+^ 423.1128, found 423.1119.

**[Pt^IV^(NH_3_)_2_(11)(OH)Cl_2_] (1)**

**13** (0.321 g, 0.801 mmol) dissolved in DMSO (15 mL) was added dropwise overnight to a suspension of Oxoplatin (0.280 g, 0.841 mmol, 1.05 equiv.) in DMSO (5 mL) and stirred in the dark at 40 °C for 48 h. The yellow suspension was filtered and the DMSO was evaporated via lyophilization. The remaining oil was treated with methanol (10 mL) and the white/yellow precipitate that was formed was washed with DCM and diethyl ether. The product was dried under reduced pressure (Yield 0.293 g, 0.473 mmol, 59%). $[\alpha {]}_{D}^{22.5}$+2.22 (c 0.9, H_2_O). ^1^H NMR (500 MHz, DMSO) δ 8.06 (s, 1H, triaz-C*H*), 6.17–5.79 (m, 6H, 2x NH_3_), 5.44 (d, *J* = 9.3 Hz, 1H, H-1), 5.32 (d, *J* = 6.0 Hz, 1H, O*H* of C-2), 5.27 (d, *J* = 4.8 Hz, 1H, O*H* of C-3), 5.14 (d, *J* = 5.4 Hz, 1H, O*H* of C-4), 4.63 (t, *J* = 5.6 Hz, 1H, O*H* of C-6), 3.77–3.65 (m, 2H, H-2, H-6), 3.43 (dt, *J* = 11.9, 4.9 Hz, 3H, H-6′, H-3, H-5), 3.21 (dd, *J* = 8.9, 5.2 Hz, 1H, H-4), 2.83 (t, *J* = 7.6 Hz, 2H, triaz-C*H_2_*), 2.49 (m, 2H, C*H*_2_CO). ^13^C NMR (125 MHz, DMSO) δ 179.89 (*C*O), 146.33 (triaz-*C*), 121.23 (triaz-*C*H), 87.42 (C-1), 79.90 (C-5), 76.97 (C-3), 72.01 (C-2), 69.60 (C-4), 60.77 (C-6), 35.96 (C*H_2_*CO), 21.93 (triaz-*C*H_2_). ^195^Pt{^1^H} NMR (108 MHz, DMSO) δ 1047.07 ppm. IR (ATR) 3221.35, 1619.15, 1351.11, 1094.74, 579.32, 438.05 cm^−1^. HR-MS (+): *m*/*z* calcd for C_11_H_23_Cl_2_N_5_O_8_Pt + H^+^ [M + H]^+^ 619.0644, found 620.0639. El. Anal. Calcd. for C_11_H_23_Cl_2_N_5_O_8_Pt: % C = 21.33; H = 3.74; N = 11.31; found: % C = 21.85; H = 3.39; N = 11.82.

***N*-(*β*-D-galactopyranosyl-1,2,3-triazol-4-yl)-propanoic acid (12)**

Compound **12** was synthesised according to the procedure reported for compound 11 (Yield 0.098 g, 0.323 mmol, 97%). R_f_ = 0.51 (DCM:MeOH:H_2_O 60:35:5) $[\alpha {]}_{D}^{23.7}$+ 16 (c 0.625, MeOH). ^1^H NMR (500 MHz, D_2_O) δ 7.96 (s, 1H, triaz-C*H*), 5.55 (d, *J* = 9.2 Hz, 1H, H-1), 4.11 (t, *J* = 9.5 Hz, 1H, H-2), 3.99 (dd, *J* = 3.3, 0.7 Hz, 1H, H-4), 3.89 (td, *J* = 6.0, 0.8 Hz, 1H, H-5), 3.78 (dd, *J* = 9.8, 3.3 Hz, 1H, H-3), 3.69 (d, *J* = 6.2 Hz, 2H, H-6, H-6′), 2.89 (t, *J* = 7.4 Hz, 2H, triaz-C*H_2_*), 2.52 (t, *J* = 7.4 Hz, 2H, C*H_2_*CO). ^13^C NMR (125 MHz, D_2_O) δ 179.95 (*C*O), 147.63 (*C*-triaz), 121.90 (*C*H-triaz), 87.93 (C-1), 78.16 (C-5), 72.92 (C-3), 69.63 (C-2), 68.52 (C-4), 60.78 (C-6), 35.46 (*C*H_2_CO), 21.20 (triaz-*C*H_2_). IR (ATR) 3247.67, 1565.21, 1399.66, 1053.32, 883.22, 553.54 cm^−1^. HR-MS (+): *m*/*z* calcd for C_11_H_17_N_3_O_7_ + H^+^ [M + H]^+^ 304.1145, found 304.1138. HR-MS (+): *m*/*z* calcd for C_11_H_17_N_3_O_7_ + Na^+^ [M + Na]^+^ 326.0964, found 326.0956.

***N*** **-(*β*-D-galactopyranosyl-1,2,3-triazol-4-yl)-(3-oxopropyl-(oxy(2,5-dioxopyrrolidin-1-yl))) (14)**

Compound **14** was prepared according to the method reported for compound 13 (Yield 0.204 g, 0.509 mmol, 68%). R_f_ = 0.9 (DCM:MeOH:H_2_O 60:35:5). $[\alpha {]}_{D}^{23.5}$ − 4.28 (c 0.7, H_2_O). ^1^H NMR (500 MHz, DMSO) δ 8.07 (s, 1H, triaz-C*H*), 5.44 (d, *J* = 9.2 Hz, 1H, H-1), 5.16 (d, *J* = 6.0 Hz, 1H, O*H* of C-2), 5.03 (d, *J* = 5.7 Hz, 1H, O*H* of C-3), 4.70 (t, *J* = 5.7 Hz, 1H, O*H* of C-6), 4.66 (d, *J* = 4.9 Hz, 1H, O*H* of C-4), 4.03–3.96 (m, 1H, H-2), 3.76 (t, *J* = 3.75 Hz, 1H, H-4), 3.70 (t, *J* = 6.1 Hz, 1H, H-5), 3.56–3.44 (m, 3H, H-3, H-6, H-6′), 3.09 (t, *J* = 7.0 Hz, 2H, C*H_2_*CO), 3.02–2.99 (m, 2H, triaz-C*H*_2_), 2.82 (s, 2H, C*H*_2_C*H*_2_-succ). ^13^C NMR (125 MHz, DMSO) δ 170.23 (*C*O succ ×2), 168.39 (*C*O), 144.42 (triaz-*C*), 121.12 (triaz-*C*H), 88.06 (C-1), 78.37 (C-5), 73.670 (C-3), 69.32 (C-2), 68.42 (C-4), 60.45 (C-6), 29.59 (*C*H_2_CO), 25.46 (*C*H_2_*C*H_2_-succ), 20.28 (triaz-*C*H_2_) ppm. IR (ATR) 3309.24, 2917.95, 1731.99, 1368.41, 1205.61, 1020.46, 891.00, 820.54, 647.48 cm^−1^. HR-MS (+): *m*/*z* calcd for C_15_H_20_N_4_O_9_ + H^+^ [M + H]^+^ 401.1309, found 401.1305. HR-MS (+): *m*/*z* calcd for C_15_H_20_N_4_O_9_ + Na^+^ [M + Na]^+^ 423.1128, found 423.1127.

**[Pt^IV^(NH_3_)_2_(12)(OH)Cl_2_] (2)**

Complex **2** was synthesised according to the method reported for complex 1 (Yield 0.111 g, 0.179 mmol, 35%). $[\alpha {]}_{D}^{23.5}$+ 10 (c 0.9, H_2_O). ^1^H NMR (500 MHz, DMSO) δ 8.00 (s, 1H, triaz-C*H*), 6.11–5.80 (br. t, 6H, 2x NH_3_), 5.39 (d, *J* = 9.2 Hz, 1H, H-1), 5.18 (d, *J* = 5.9 Hz, 1H, O*H* of C-2), 5.03 (d, *J* = 5.6 Hz, 1H, O*H* of C-3), 4.70 (t, *J* = 5.6 Hz, 1H, O*H* of C-6), 4.64 (d, *J* = 5.1 Hz, 1H, O*H* of C-4), 4.05–3.98 (m, 1H, H-2), 3.75 (t, *J* = 3.6 Hz, 1H, H-4), 3.68 (t, *J* = 5.9 Hz, 1H, H-5), 3.56–3.47 (m, 3H, H-3, H-6, H-6′), 2.83 (t, *J* = 7.45 Hz, 2H, triaz-C*H_2_*), 2.53–2.51 (m, 2H, C*H_2_*CO, overlaps with DMSO). ^13^C NMR (125 MHz, DMSO) δ 179.90 (*C*O), 146.35 (triaz-*C*), 120.97 (triaz-*C*H), 88.01 (C-1), 78.35 (C-5), 73.73 (C-3), 69.19 (C-2), 68.46 (C-4), 60.47 (C-6), 36.01 (*C*H_2_CO), 21.94 (triaz-*C*H_2_). ^195^Pt{^1^H} NMR (108 MHz, DMSO) δ 1045.09 ppm. IR (ATR) 3224.69, 1617.95, 1352.08, 1090.21, 890.51, 563.88, 437.95 cm^−1^. HR-MS (+): *m*/*z* calcd for C_11_H_23_Cl_2_N_5_O_8_Pt + H^+^ [M + H]^+^ 619.0644, found 620.0634. El. Anal. Calcd. for C_11_H_23_Cl_2_N_5_O_8_Pt: % C = 21.33; H = 3.74; N = 11.31; found: % C = 21.64; H = 3.82; N = 11.72.

***N*** **-[2-*O*-(*β*-D-glucopyranosyl)-ethyl-1,2,3-triazol-4-yl)]-propanoic acid (21)**

Compound **21** was synthesised according to the procedure used for compound 11 (Yield 0.053 g, 0.152 mmol, 89%). R_f_ = 0.56 (DCM:MeOH:H_2_O 60:35:5). $[\alpha {]}_{D}^{24.4}$+4.18 (c 0.716, MeOH). ^1^H NMR (500 MHz, D_2_O) δ 7.80 (s, 1H, triaz-C*H*), 4.57 (dd, *J* = 14.3, 3.5 Hz, 2H, C*H_2_*-triaz), 4.35 (d, *J* = 6.8 Hz, 1H, H-1), 4.27–4.20 (m, 1H, OC*H*), 4.05 (dt, *J* = 6.2, 4.1 Hz, 1H, OC*H’*), 3.84 (d, *J* = 12.3 Hz, 1H, H-6), 3.67–3.60 (m, 1H, H-6′), 3.43–3.27 (m, 3H, H-3, H-4, H-5), 3.21–3.18 (m, 5H, H-2), 2.92 (td, *J* = 7.3, 1.3 Hz, 2H, triaz-C*H_2_*), 2.53 (t, *J* = 7.4 Hz, 2H, C*H_2_*CO). ^13^C NMR (125 MHz, D_2_O) δ 180.75 (*C*O), 147.40 (triaz-*C*), 123.72 (triaz-*C*H), 102.38 (C-1), 75.87 (C-3), 75.55 (C-5), 72.89 (C-2), 69.51 (C-4), 68.09 (O*C*H_2_), 60.65 (C-6), 50.11 (*C*H_2_-triaz), 36.01 (*C*H_2_CO), 21.38 (triaz-*C*H_2_). IR (ATR) 3334.48, 2885.68, 1711.54, 1553.96, 1358.20, 1219.58, 1030.84, 828.66, 494.93 cm^−1^. HR-MS (+): *m*/*z* calcd for C_13_H_21_N_3_O_8_ + H^+^ [M + H]^+^ 348.1407, found 348.1402. HR-MS (+): *m*/*z* calcd for C_13_H_21_N_3_O_8_ + Na^+^ [M + Na]^+^ 370.1226, found 370.1218.

***N*** **-[2-*O*-*(β*-D-glucopyranosyl)-ethyl-1,2,3-triazol-4-yl)]-(3-oxopropyl-(oxy(2,5-dioxopyrrolidin-1-yl))) (23)**

Compound **23** was synthesised according to the procedure for compound 13. (Yield 0.380 g, 0.855 mmol, 80%). R_f_ = 0.86 (DCM:MeOH:H_2_O 60:35:5). $[\alpha {]}_{D}^{23.2}$+ 1.07 (c 0.93, MeOH). ^1^H NMR (500 MHz, DMSO) δ 7.99 (s, 1H, triaz-C*H*), 5.10 (d, *J* = 5.0 Hz, 1H, O*H* of C-2), 4.99 (d, *J* = 4.8 Hz, 1H, O*H* of C-3), 4.94 (d, *J* = 5.3 Hz, 1H, O*H* of C-4), 4.52 (dt, *J* = 6.8, 4.2 Hz, 3H, C*H_2_*-triaz, O*H* of C-6), 4.21 (d, *J* = 7.8 Hz, 1H, H-1), 4.06 (ddd, *J* = 10.6, 5.9, 4.4 Hz, 1H, OC*H*), 3.86 (ddd, *J* = 11.2, 6.7, 4.4 Hz, 1H, OC*H’*), 3.67 (ddd, *J* = 11.5, 5.9, 1.8 Hz, 1H, H-6), 3.43 (dt, *J* = 11.7, 5.9 Hz, 1H, H-6′), 3.16–3.08 (m, 2H, H-3, H-5), 3.08–3.01 (m, 3H, C*H_2_*CO, H-4), 3.00–2.93 (m, 3H, H-2, triaz-C*H*_2_), 2.81 (s, 4H, C*H_2_*C*H_2_*-succ). ^13^C NMR (125 MHz, DMSO) δ 170.20 (*C*O succ x2), 168.33 (*C*O), 144.17 (triaz-*C*), 123.18 (triaz-*C*H), 102.86 (C-1), 76.97 (C-3), 76.57 (C-5), 73.31 (C-2), 70.01 (C-4), 67.31 (O*C*H_2_), 61.08 (C-6), 49.54 (*C*H_2_-triaz), 29.81 (*C*H_2_CO), 25.45 (*C*H_2_*C*H_2_-succ), 20.37 (triaz-*C*H_2_). IR (ATR) 3369.66, 2886.72, 1728.66, 1366.57, 1206.12, 1033.96, 813.22, 645.50 cm^−1^. HR-MS (+): *m*/*z* calcd for C_17_H_24_N_4_O_10_ + H^+^ [M + H]^+^ 445.1571, found 445.1561. HR-MS (+): *m*/*z* calcd for C_17_H_24_N_4_O_10_ + Na^+^ [M + Na]^+^ 467.1390, found 467.1384.

**[Pt^IV^(NH_3_)_2_(21)(OH)Cl_2_] (3)**

Complex **3** prepared according to the method reported for complex 1. (Yield 0.218 g, 0.328 mmol, 47%). $[\alpha {]}_{D}^{21.8}$*+* 15 (c 0.4, H_2_O). ^1^H NMR (500 MHz, DMSO) δ 7.93 (s, 1H, triaz-C*H*), 5.98 (t, *J* = 48.2 Hz, 6H, 2x NH_3_), 5.10 (d, *J* = 4.9 Hz, 1H, O*H* of C-2), 4.98 (d, *J* = 4.7 Hz, 1H, O*H* of C-3), 4.93 (d, *J* = 5.2 Hz, 1H, O*H* of C-4), 4.57–4.46 (m, 3H, C*H*_2_-triaz, O*H* of C-6), 4.21 (d, *J* = 7.8 Hz, 1H, H-1), 4.10–4.03 (m, 1H, OC*H*), 3.89–3.83 (m, 1H, OC*H’*), 3.67 (dd, *J* = 10.7, 4.4 Hz, 1H, H-6), 3.43 (dd, *J* = 11.4, 5.6 Hz, 1H, H-6′), 3.16–3.09 (m, 2H, H-3, H-5), 3.03 (dd, *J* = 9.0, 4.0 Hz, 1H, H-4), 2.98–2.93 (m, 1H, H-2), 2.80 (t, *J* = 7.2 Hz, 2H, triaz-C*H*_2_), 2.46 (d, *J* = 7.3 Hz, 2H, C*H*_2_CO). ^13^C NMR (125 MHz, DMSO) δ 179.94 (*C*O), 146.18 (triaz-*C*), 122.90 (triaz-*C*H), 102.82 (C-1), 76.95 (C-3), 76.54 (C-5), 73.32 (C-2), 69.99 (C-4), 67.27 (O*C*H_2_), 61.06 (C-6), 49.42 (*C*H_2_-triaz), 36.12 (*C*H_2_CO), 22.01 (triaz-*C*H_2_). ^195^Pt{^1^H} NMR (108 MHz, DMSO) δ 1047.34 ppm. IR (ATR) 3247.15, 1623.71, 1356.54, 1035.26, 578.92, 426.46 cm^−1^. HR-MS (+): *m*/*z* calcd for C_13_H_27_Cl_2_N_5_O_9_Pt + H^+^ [M + H]^+^ 663.0907, found 664.0904. El. Anal. Calcd. for C_13_H_27_Cl_2_N_5_O_9_Pt: % C = 23.54; H = 4.10; N = 10.56; found: % C = 23.27; H = 4.91; N = 10.11.

***N*** **-[2-*O*-(*β*-D-galactopyranosyl)-ethyl-1,2,3-triazol-4-yl)]-propanoic acid** **(22)**

Compound **22** was prepared according to the procedure reported for compound 11 (Yield 0.231 g, 0.665 mmol, 96%). R_f_ = 0.30 (DCM:MeOH:H_2_O 60:35:5). $[\alpha {]}_{D}^{23.2}$+ 4.24 (c 0.707, MeOH). ^1^H NMR (500 MHz, D_2_O) δ 7.85 (s, 1H, triaz-C*H*), 4.61 (t, *J* = 5.1 Hz, 2H, C*H_2_*-triaz), 4.31 (d, *J* = 7.9 Hz, 1H, H-1), 4.26 (dt, *J* = 11.5, 4.7 Hz, 1H, OC*H*), 4.09–4.04 (m, 1H, OC*H*’), 3.87 (d, *J* = 3.4 Hz, 1H, H-4), 3.75–3.67 (m, 2H, H-6, H-6′), 3.62 (dd, *J* = 7.7, 4.6 Hz, 1H, H-5), 3.58 (dd, *J* = 9.9, 3.5 Hz, 1H, H-3), 3.45 (dd, *J* = 9.9, 7.9 Hz, 1H, H-2), 2.96 (t, *J* = 7.3 Hz, 2H, triaz-C*H*_2_), 2.65 (t, *J* = 7.3 Hz, 2H, C*H_2_*CO). ^13^C NMR (125 MHz, D_2_O) δ 178.90 (*C*O), 146.93 (*C*-triaz), 123.87 (*C*H-triaz), 102.97 (C-1), 75.08 (C-5), 72.58 (C-3), 70.55 (C-2), 68.53 (C-4), 68.02 (O*C*H_2_), 60.87 (C-6), 50.19 (*C*H_2_-triaz), 34.49 (*C*H_2_CO), 20.72 (triaz-*C*H_2_). IR (ATR) 3282.63, 2926.54, 1568.45, 1398.02, 1044.17, 781.51, 533.03 cm^−1^. HR-MS (+): *m*/*z* calcd for C_13_H_21_N_3_O_8_ + H^+^ [M + H]^+^ 348.1407, found 348.1403. HR-MS (+): *m*/*z* calcd for C_13_H_21_N_3_O_8_ + Na^+^ [M + Na]^+^ 370.1226, found 370.1221.

***N*** **-[2-*O*-(*β*-D-galactopyranosyl)-ethyl-1,2,3-triazol-4-yl)]-(3-oxopropyl-(oxy(2,5-dioxopyrrolidin-1-yl))) (24)**

Compound **24** was prepared according to the procedure reported for compound 13 (Yield 0.360 g, 0.810 mmol, 75%). R_f_ = 0.84 (DCM:MeOH:H_2_O 60:35:5). $[\alpha {]}_{D}^{22.8}$+ 5.5 (c 0.366, MeOH). ^1^H NMR (500 MHz, DMSO) δ 7.99 (s, 1H, triaz-C*H*), 4.95 (d, *J* = 4.6 Hz, 1H, O*H* of C-2), 4.76 (d, *J* = 5.3 Hz, 1H, O*H* of C-3), 4.59 (t, *J* = 5.7 Hz, 1H, O*H* of C-6), 4.51 (qdd, *J* = 14.3, 6.4, 4.2 Hz, 2H, C*H*_2_-triaz), 4.39 (d, *J* = 4.6 Hz, 1H, O*H* of C-4), 4.15 (d, *J* = 7.3 Hz, 1H, H-1), 4.05 (ddd, *J* = 10.7, 6.2, 4.3 Hz, 1H, OC*H*), 3.83 (ddd, *J* = 11.1, 6.7, 4.3 Hz, 1H, OC*H’*), 3.63–3.60 (m, 1H, H-4), 3.55–3.44 (m, 2H, H-6, H-6′), 3.34–3.24 (m, 3H, H-2, H-5, H-3), 3.07–3.03 (m, 2H, C*H*_2_CO), 3.00–2.95 (m, 2H, triaz-C*H*_2_), 2.81 (s, 4H, C*H_2_*C*H_2_*-succ). ^13^C NMR (125 MHz, DMSO) δ 170.22 (*C*O succ x2), 168.35 (*C*O), 144.19 (triaz-*C*), 123.16 (triaz-*C*H), 103.48 (C-1), 75.37 (C-5), 73.30 (C-3), 70.41 (C-2), 68.15 (C-4), 67.20 (O*C*H_2_), 60.46 (C-6), 49.56 (*C*H_2_-triaz), 29.82 (*C*H_2_CO), 25.46 (*C*H_2_*C*H_2_-succ), 20.38 (triaz-*C*H_2_). IR (ATR) 3378.02, 2939.53, 1728.86, 1366.45, 1206.01, 1046.41, 648.73 cm^−1^. HR-MS (+): *m*/*z* calcd for C_17_H_24_N_4_O_10_ + H^+^ [M + H]^+^ 445.1571, found 445.1559. HR-MS (+): *m*/*z* calcd for C_17_H_24_N_4_O_10_ + Na^+^ [M + Na]^+^ 467.1390, found 467.1385.

**[Pt^IV^(NH_3_)_2_(22)(OH)Cl_2_] (4)**

Complex **4** was prepared according to the procedure reported for complex 1 (Yield 0.296 g, 0.446 mmol, 61%). $[\alpha {]}_{D}^{22.8}$+ 3.36 (c 0.9, H_2_O). ^1^H NMR (500 MHz, DMSO) δ 7.93 (s, 1H, triaz-C*H*), 5.98 (t, *J* = 48.4 Hz, 6H, 2× NH_3_), 4.96 (d, *J* = 4.3 Hz, 1H, O*H* of C-3), 4.77 (d, *J* = 4.4 Hz, 1H, O*H* of C-2), 4.59 (d, *J* = 5.1 Hz, 1H, O*H* of C-6), 4.55–4.41 (m, 2H, C*H_2_*-triaz), 4.40 (d, *J* = 4.4 Hz, 1H, OH of C-4), 4.15 (d, *J* = 7.1 Hz, 1H, H-1), 4.05 (ddd, *J* = 14.4, 9.4, 6.4 Hz, 1H, OC*H*), 3.84 (ddd, *J* = 11.0, 6.5, 4.6 Hz, 1H, OC*H’*), 3.62 (s, 1H, H-4), 3.50 (dd, *J* = 12.2, 6.4 Hz, 2H, H-6, H-6′), 3.35 (s, 1H, H-5 overlaps with H_2_O), 3.30–3.27 (m, 2H, H-2, H-3), 2.82–2.77 (m, 2H, triaz-C*H_2_*), 2.46 (d, *J* = 7.3 Hz, 2H, C*H_2_*CO). ^13^C NMR (125 MHz, DMSO) δ 179.93 (*C*O), 146.19 (triaz-*C*), 122.88 (triaz-*C*H), 103.48 (C-1), 75.37 (C-5), 73.27 (C-3), 70.43 (C-2), 68.15 (C-4), 67.19 (O*C*H_2_), 60.46 (C-6), 49.46 (*C*H_2_-triaz), 36.12 (*C*H_2_CO), 22.01 (triaz-*C*H_2_). ^195^Pt{^1^H} NMR (108 MHz, DMSO) δ 1046.82 ppm. IR (ATR) 3215.30, 1618.19, 1358.28, 1061.49, 575.94 cm^−1^. HR-MS (+): *m*/*z* calcd for C_13_H_27_Cl_2_N_5_O_9_Pt + H^+^ [M + H]^+^ 663.0907, found 664.0902. El. Anal. Calcd. for C_13_H_27_Cl_2_N_5_O_9_Pt: % C = 23.54; H = 4.10; N = 10.56; found: % C = 23.18; H = 4.75; N = 10.06.

## 4. Conclusions

Four novel Pt(IV) pro-drugs, based on a cisplatin scaffold with carbohydrate vectors in axial positions, were synthetised, linking the sugar moiety and the metal centre via CuAAC click chemistry. These pro-drugs were functionalised with deprotected glyco-moieties that act as real vectors to selectively target cancer cells. Most of the carbohydrate-functionalised Pt-based complexes reported in literature contain protected acetylated sugars, due to an easy synthesis and purification procedure. The complexes were tested on a panel of two 2D and 3D OS (Osteosarcoma) cell lines, as well as on healthy OS cells. All the complexes showed very promising activity, comparable to the reference cisplatin, demonstrating that the presence of a monosaccharide does not hamper the anticancer effect. Notably, the complexes are much less active against the healthy line, showing a promising selectivity for these OS cell lines, with respect to cisplatin. The complexes were also particularly active in the 3D model, a more reliable system compared to 2D, with the most promising activity shown by complexes **2** and **4** with a galactose substituent. The role of galactose in the metabolism of cancer cells is attracting significant attention because of potential diagnostic and therapeutic possibilities [40,41]. While the role of the free sugars as targeting vectors is not completely confirmed, the selectivity shown in 2D studies is a solid base to hypothesise that the carbohydrate moieties play an important role in targeted therapies. More specific biological studies (beyond the scope of this work) should be conducted (i.e., inhibition of the GLUTs receptors), but this selectivity was not observed in the analogue protected-Pt(IV) pro-drugs, recently reported by us. While all the complexes showed very promising activity, the discrimination between the two linkers is not observed. These complexes have been conjugated to graphene oxide nanoparticles that act as delivering agents, to further enhance the selectivity. The next step in our studies is the synthesis and characterisation of analogous complexes, where the carbohydrate is conjugated via the C2 carbon (not the anomeric carbon) that was demonstrated to be the best in term of cellular recognition [42]. 

## Figures and Tables

**Table 1:** IC_50_ (µM) values of the complexes **1**–**4** and cisplatin against MG63, SAOS-2 and hFOBs cells.

	Cisplatin		Complex 1		Complex 2		Complex 3		Complex 4	
	$IC_{50}\;$	95% CI	$IC_{50}\;$	95% CI	$IC_{50}\;$	95% CI	$IC_{50}\;$	95% CI	$IC_{50}$	95% CI
**MG63**	12.20	+3.1; −2.6	15.90	+3.3; −2.9	13.60	+5.2; −4.0	13.0	+3.2; −2.7	14.90	+4.2; −3.5
**SAOS-2**	13.70	+3.2; −2.7	12.94	+3.4; −3.0	19.09	+3.4; −3.0	15.18	+3.7; −3.1	17.22	+4.5; −3.7
**hFOBs**	4.109	+0.42; −0.47	23.36	+1.72; −1.82	20.85	+1.92; −2.11	16.88	+1.7; −1.75	20.91	+0.42; −0.47

## Data Availability

The data presented in this study are available in the article and in the Supplementary Material.

## Supplementary Materials

The following supporting information can be downloaded at: https://www.mdpi.com/article/10.3390/ijms24076028/s1.
