# Supplementary material for: Development of Novel Pt(IV)-Carbohydrate Derivatives as Targeted Anticancer Agents against Osteosarcoma
Source: Int J Mol Sci. 2023 Mar 23;24(7):6028. doi: 10.3390/ijms24076028 (PMC10094171; doi:10.3390/ijms24076028)
Supplement: Supplementary file 1 [file ijms-24-06028-s001.zip › ijms-2280092-supplementary.pdf]

Supporting Information

Development of novel Pt(IV)-Carbohydrate derivatives as targeted anticancer agents against Osteosarcoma

Eoin Moynihan, Silvia Panseri, Giada Bassi, Arianna Rossi, Elisabetta Campodoni, Eithne Dempsey, Monica Montesi, Trinidad Velasco Torrijos, Diego Montagner

NMR Spectra

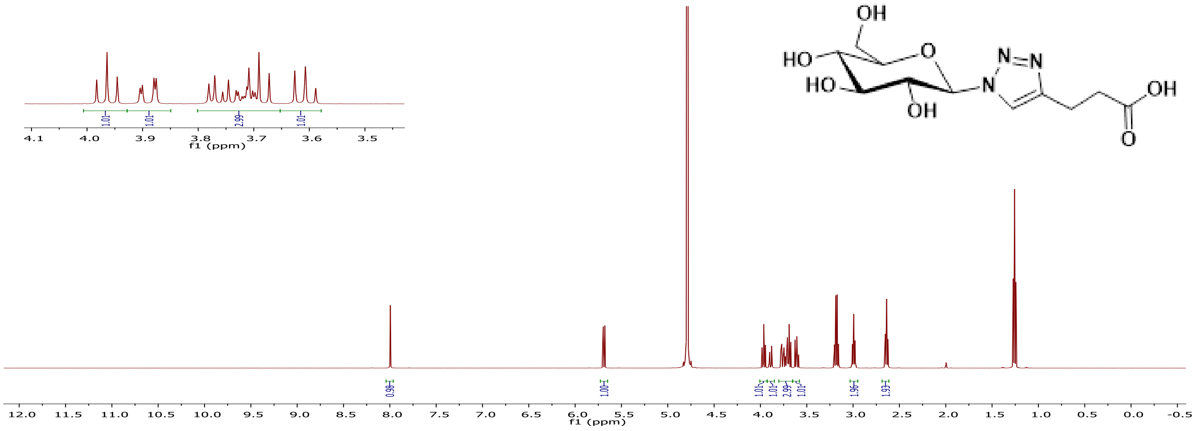

Figure S1a: ^{1}H NMR spectrum of **11** in D_2O

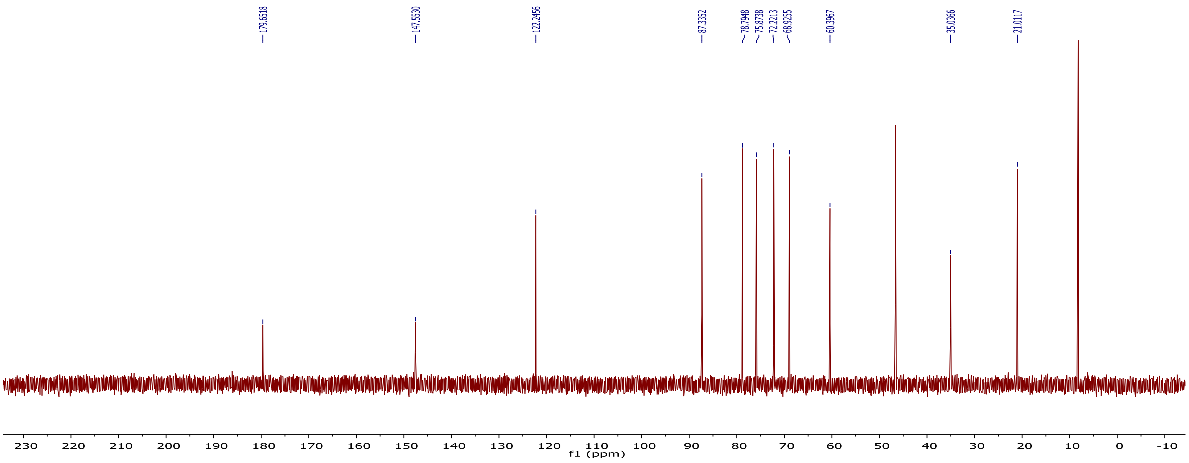

Figure S1b: ^{13}C NMR spectrum of **11** in D_2O

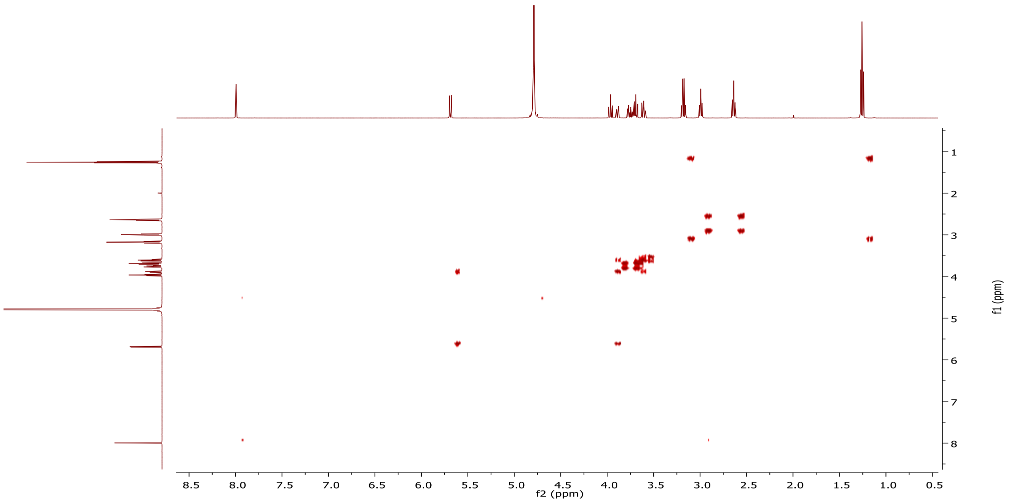

Figure S1c: COSY NMR spectrum of **11** in D_2O

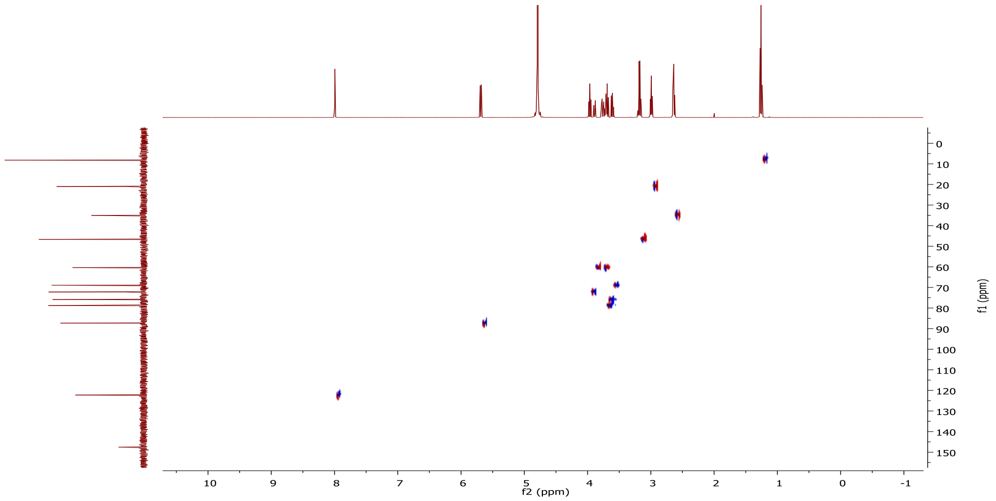

Figure S1d: HSQC NMR spectrum of **11** in D_2O

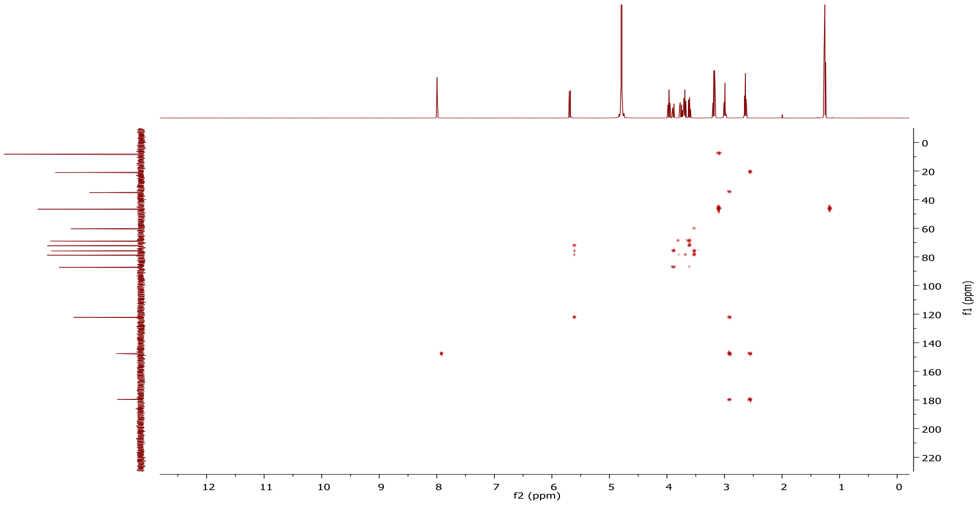

Figure S1e: HMBC NMR spectrum of **11** in D_2O

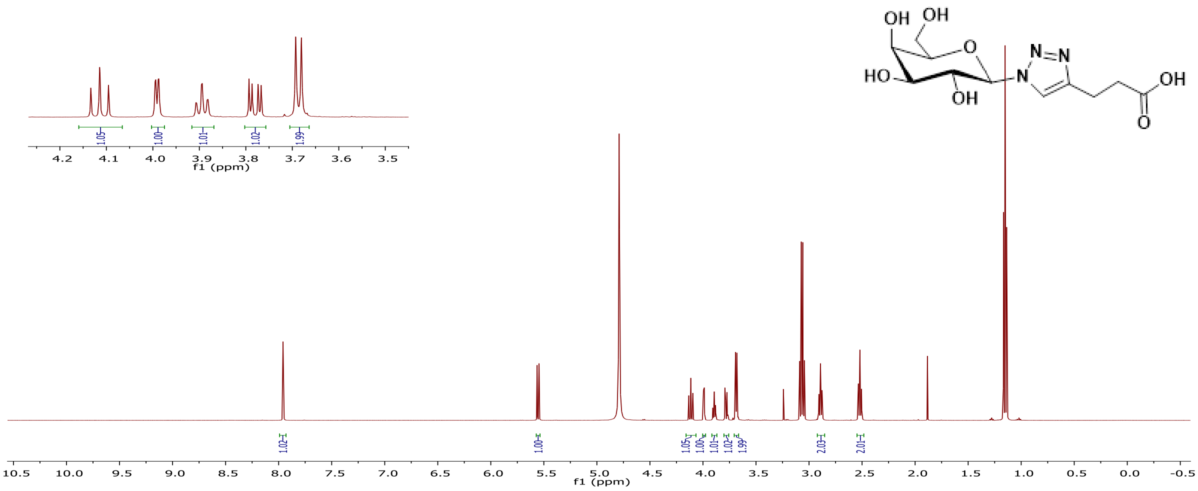

Figure S2a: ^{1}H NMR spectrum of **12** in D_2O

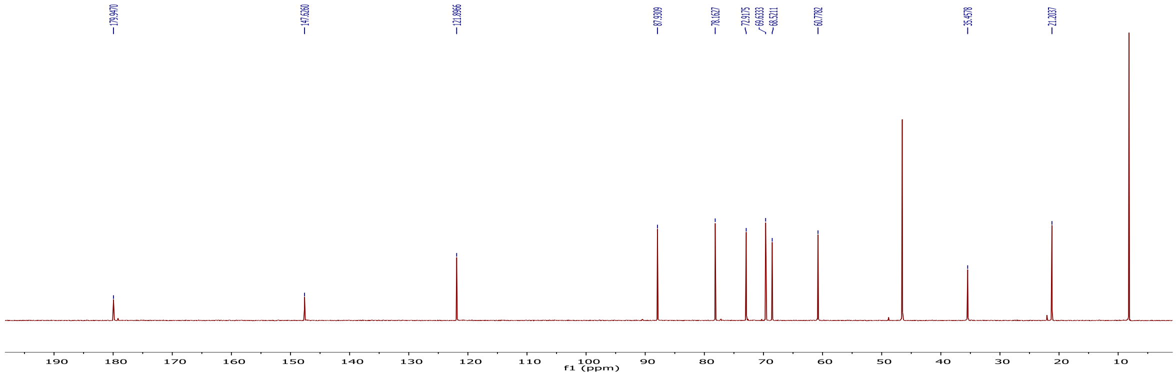

Figure S2b: ^{13}C NMR spectrum of **12** in D_2O

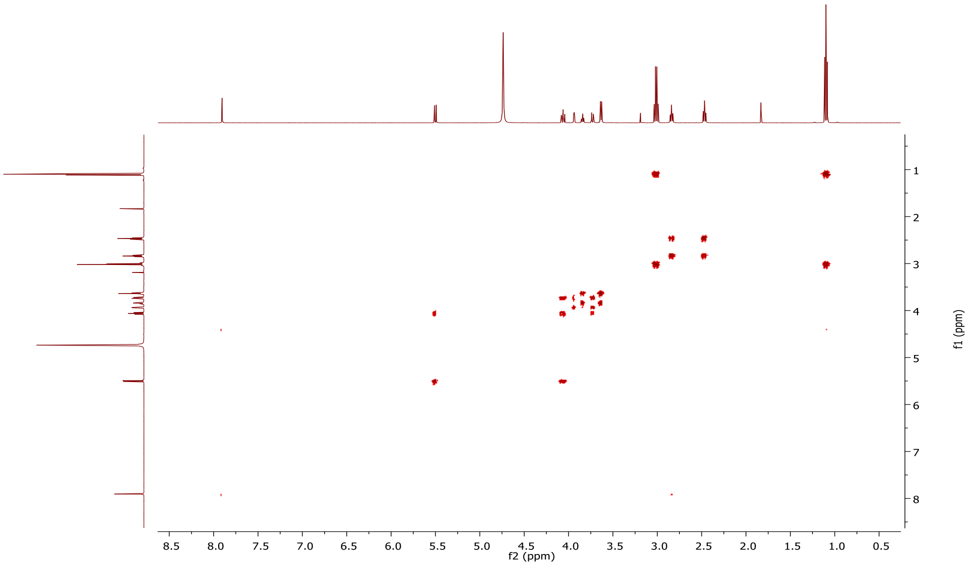

Figure S2c: COSY NMR spectrum of **12** in D_2O

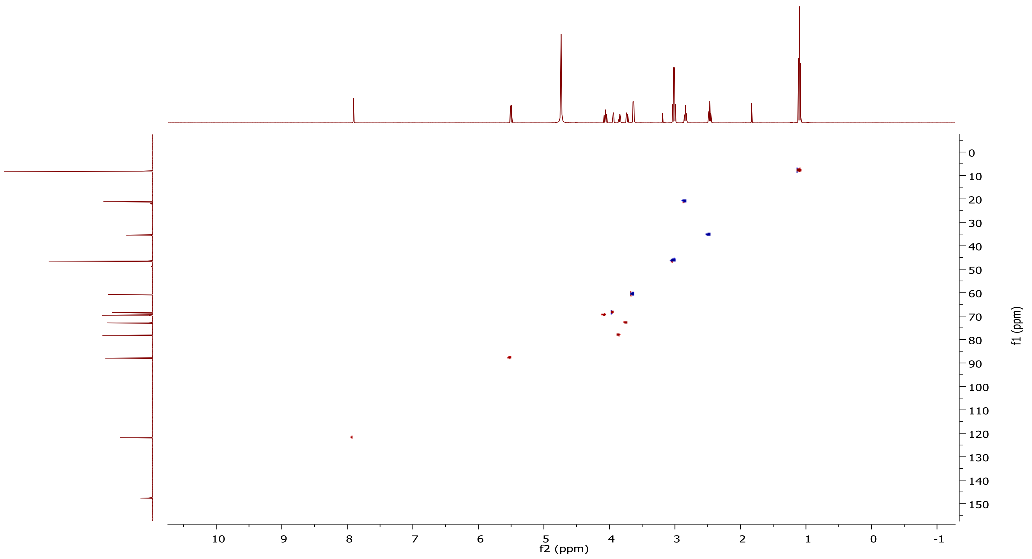

Figure S2d: HSQC NMR spectrum of **12** in D_2O

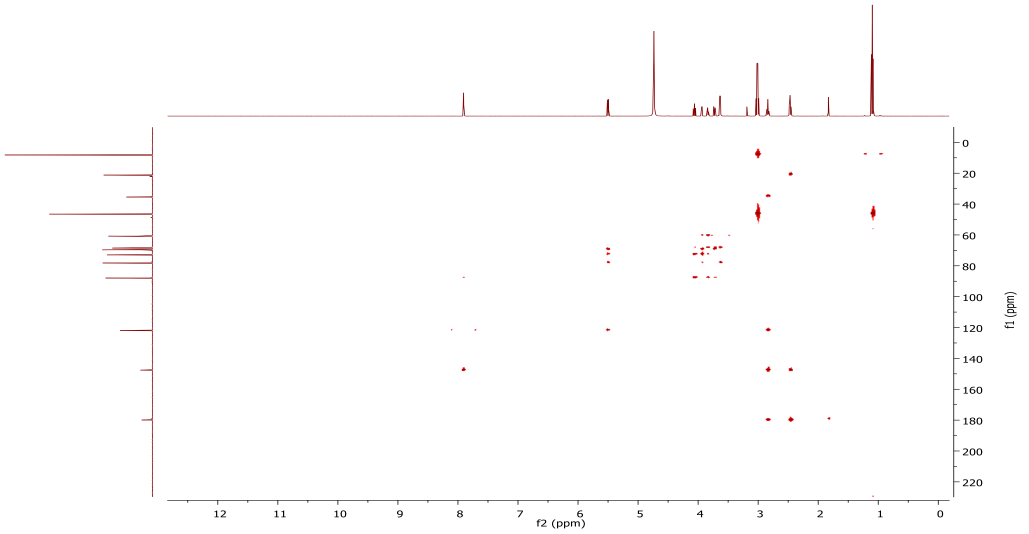

Figure S2e: HMBC NMR spectrum of **12** in D_2O

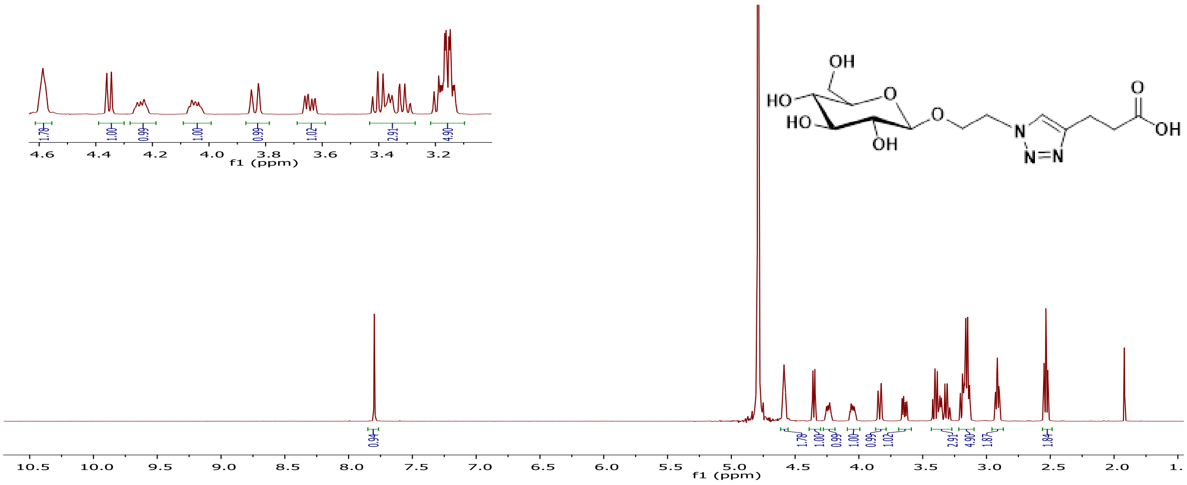

Figure S3a: ^{1}H NMR spectrum of **21** in D_2O

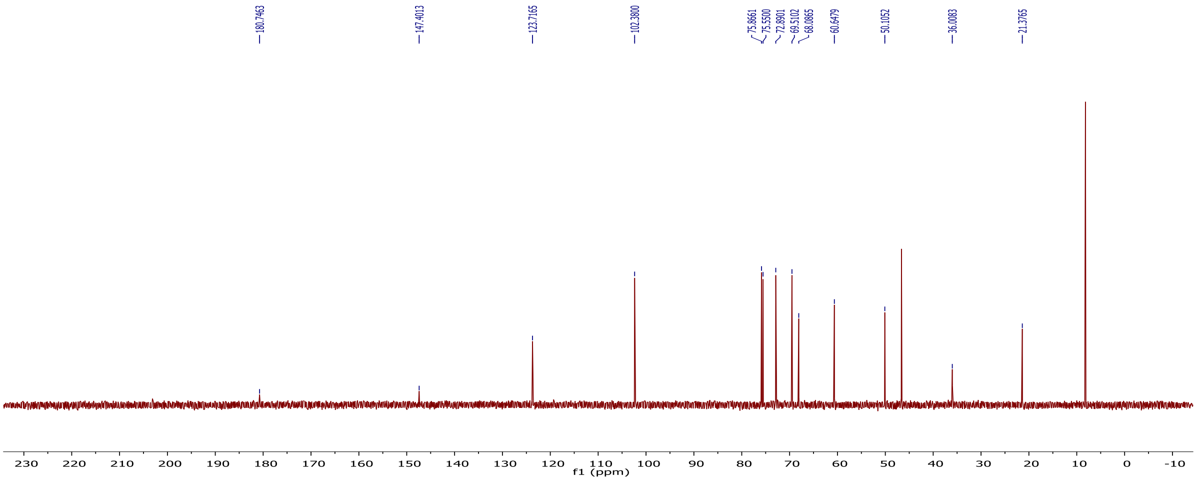

Figure S3b: ^{13}C NMR spectrum of **21** in D_2O

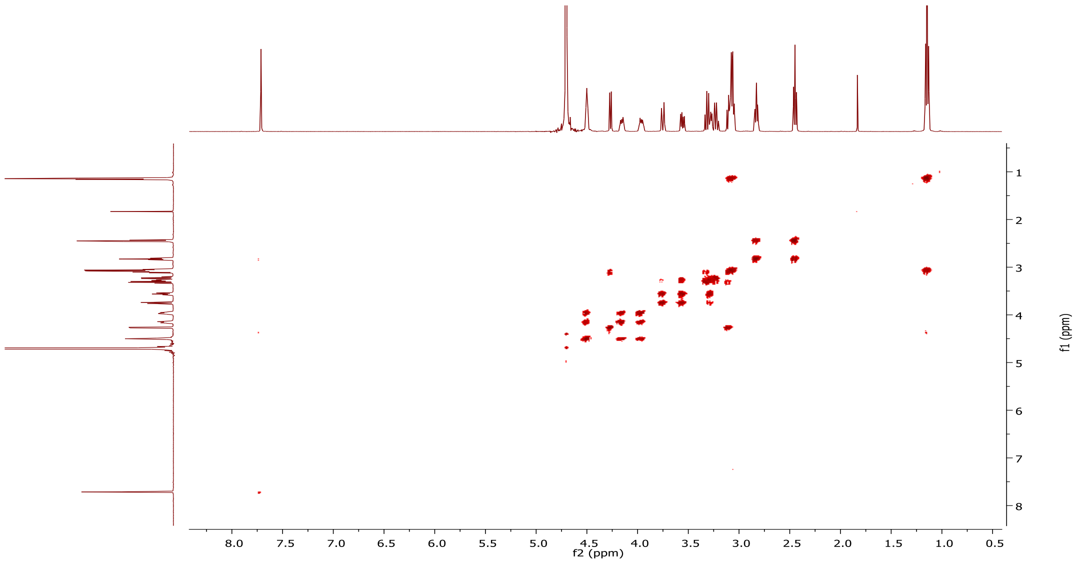

Figure S3c: COSY NMR spectrum of **21** in D_2O

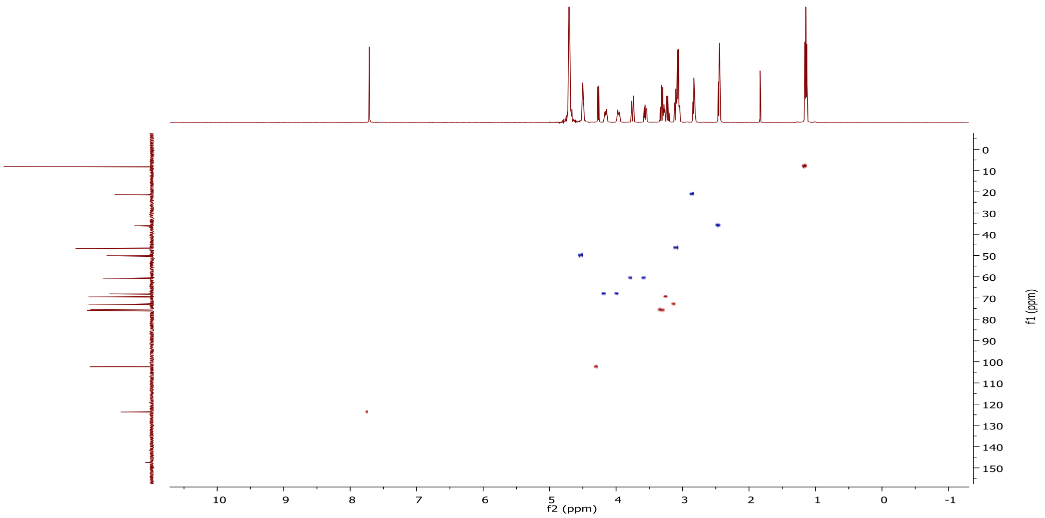

Figure S3d: HSQC NMR spectrum of **21** in D_2O

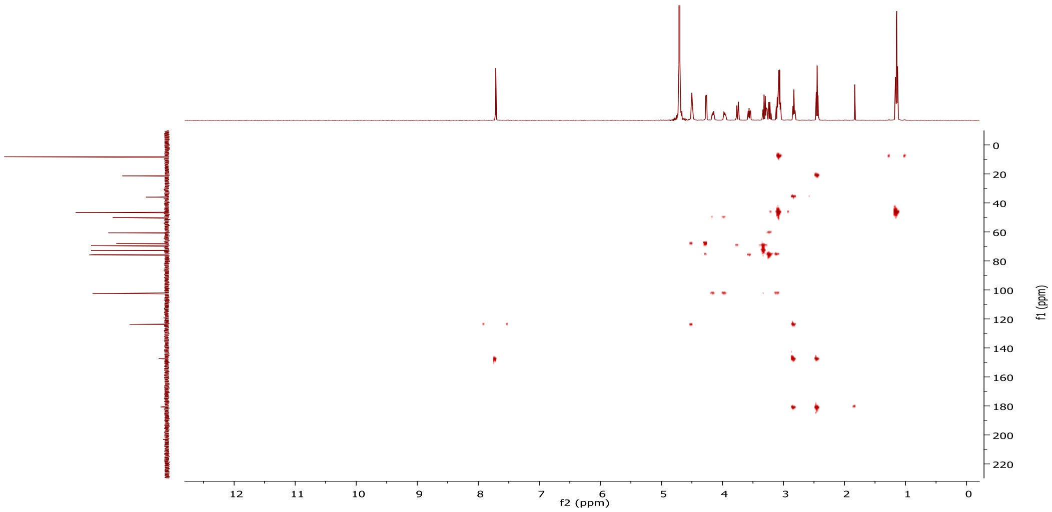

Figure S3e: HMBC NMR spectrum of **21** in D_2O

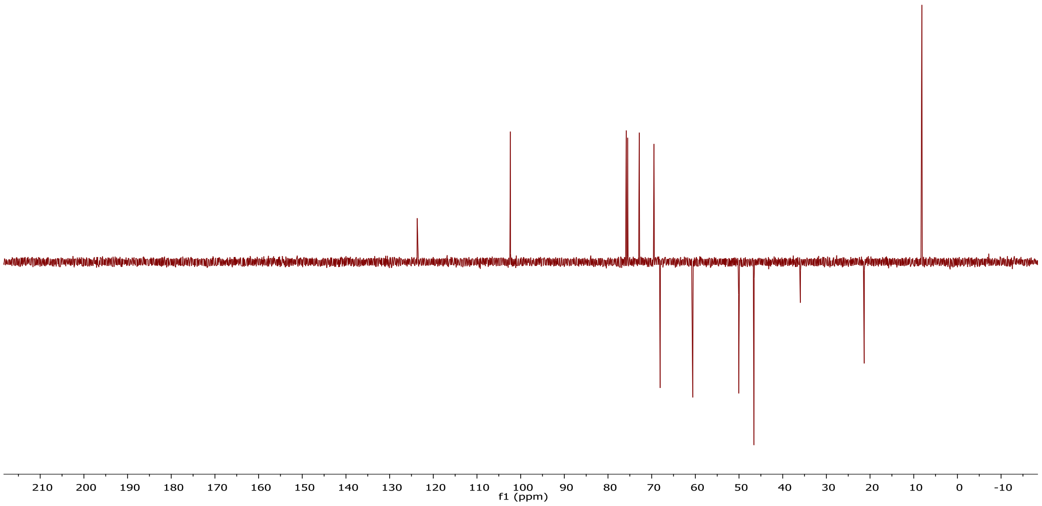

Figure S3f: DEPT-135 NMR spectrum of **21** in D_2O

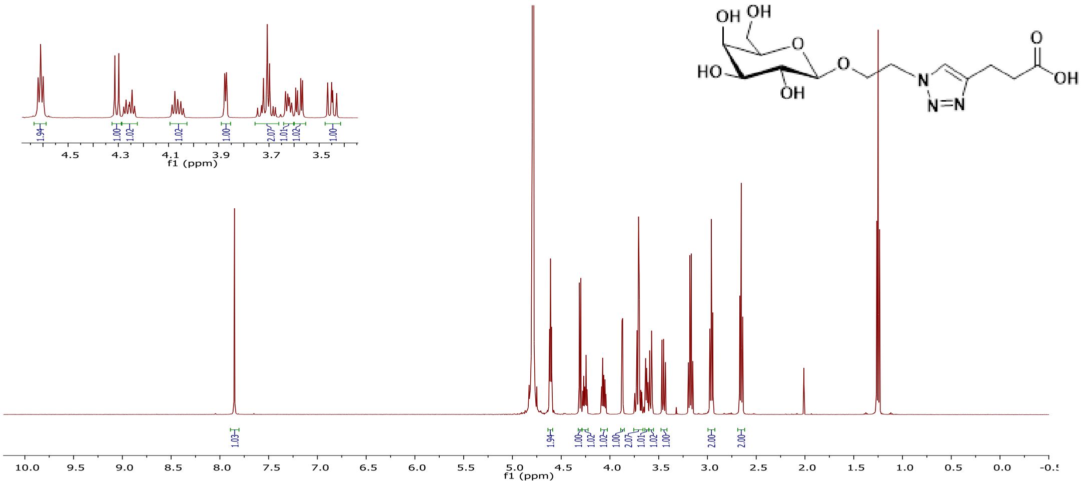

Figure S4a: ^{1}H NMR spectrum of **22** in D_2O

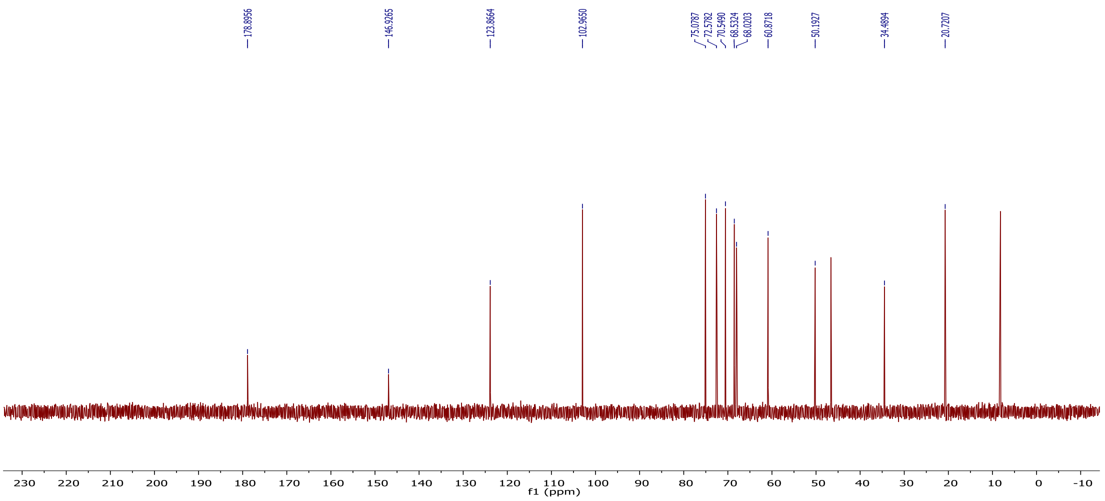

Figure S4b: ^{13}C NMR spectrum of **22** in D_2O

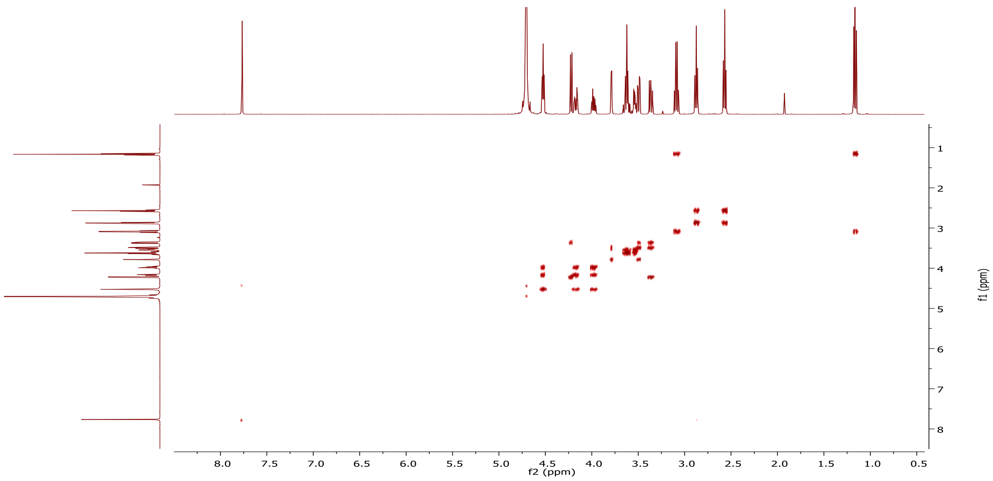

Figure S4c: COSY NMR spectrum of **22** in D_2O

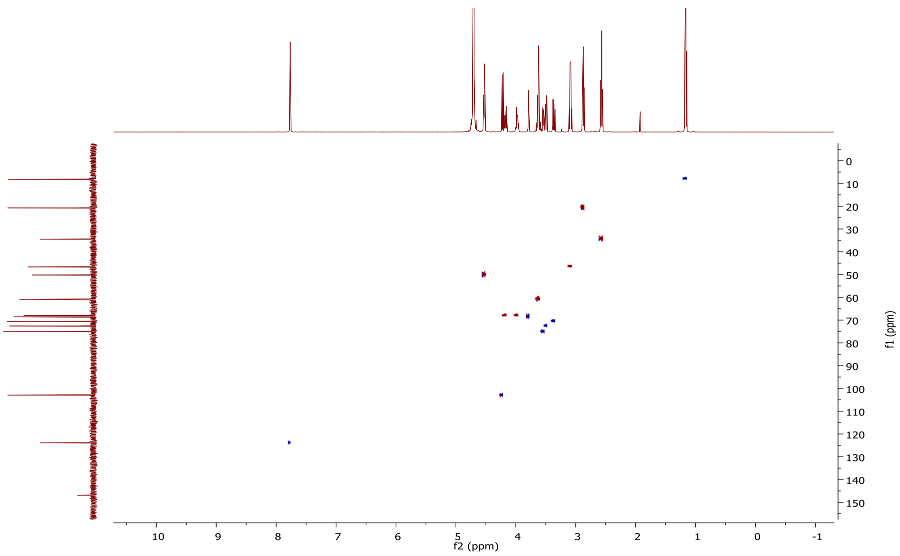

Figure S4d: HSQC NMR spectrum of **22** in D_2O

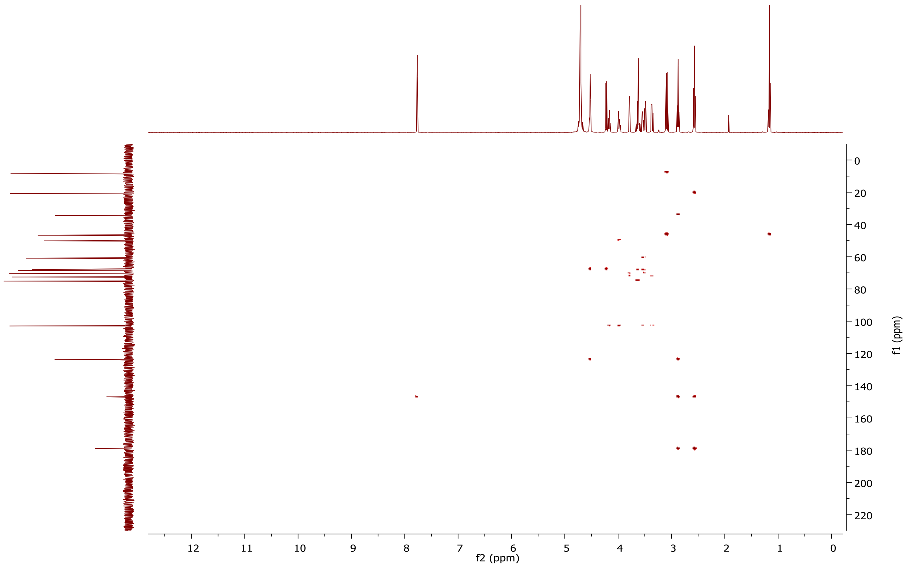

Figure S4e: HMBC NMR spectrum of **22** in D_2O

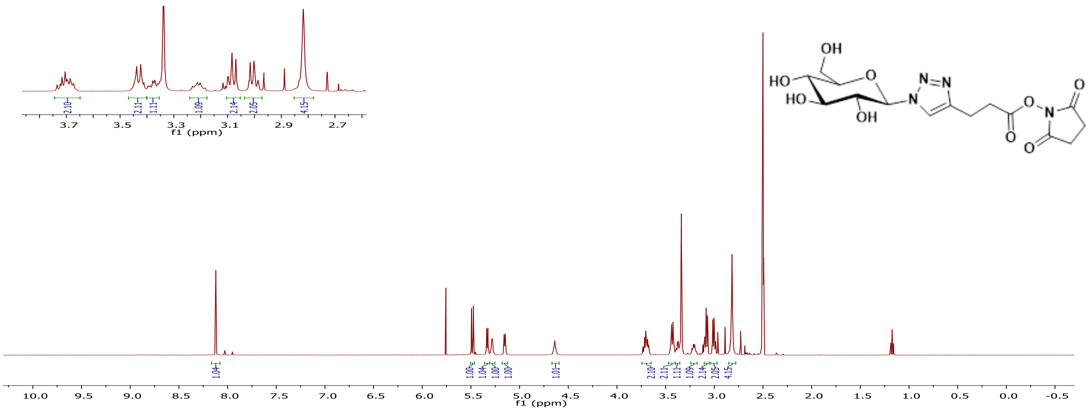

Figure S5a: ^{1}H NMR spectrum of **13** in DMSO

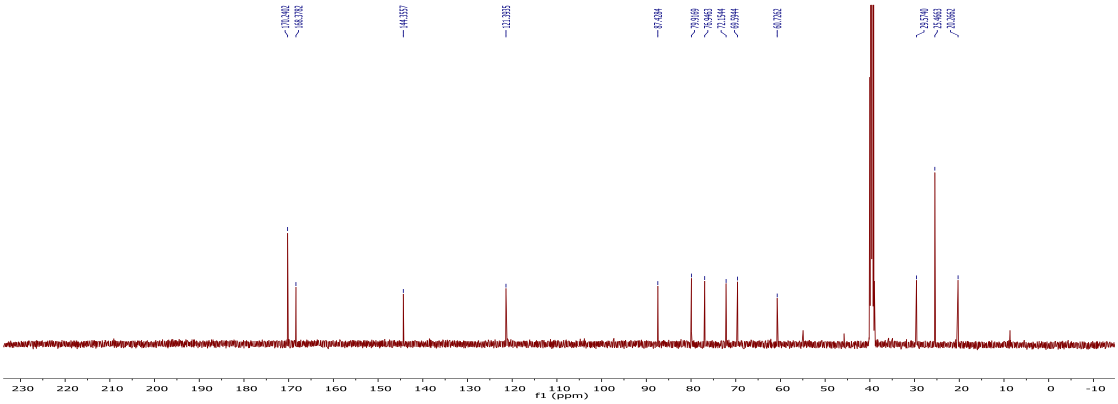

Figure S5b: ^{13}C NMR spectrum of **13** in DMSO

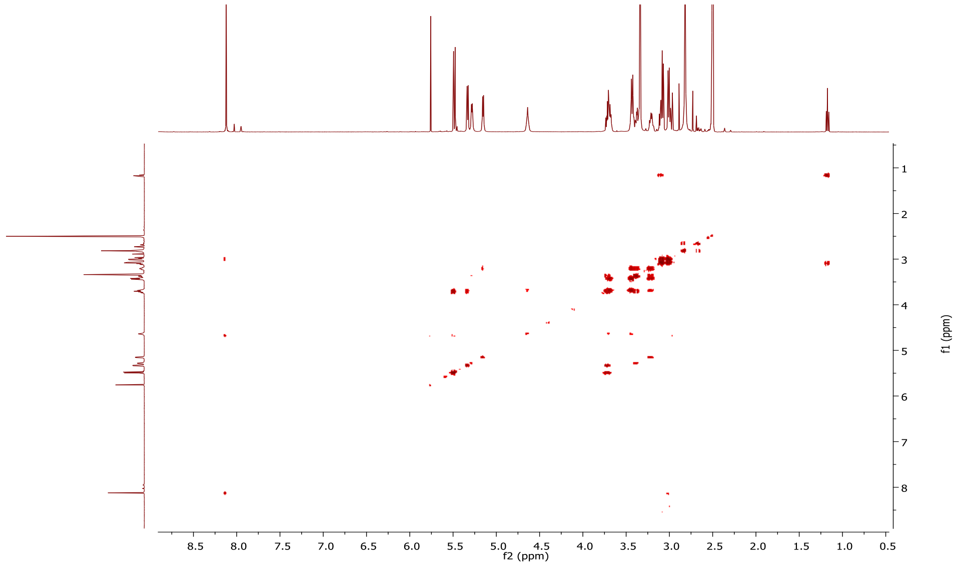

Figure S5c: COSY NMR spectrum of **13** in DMSO

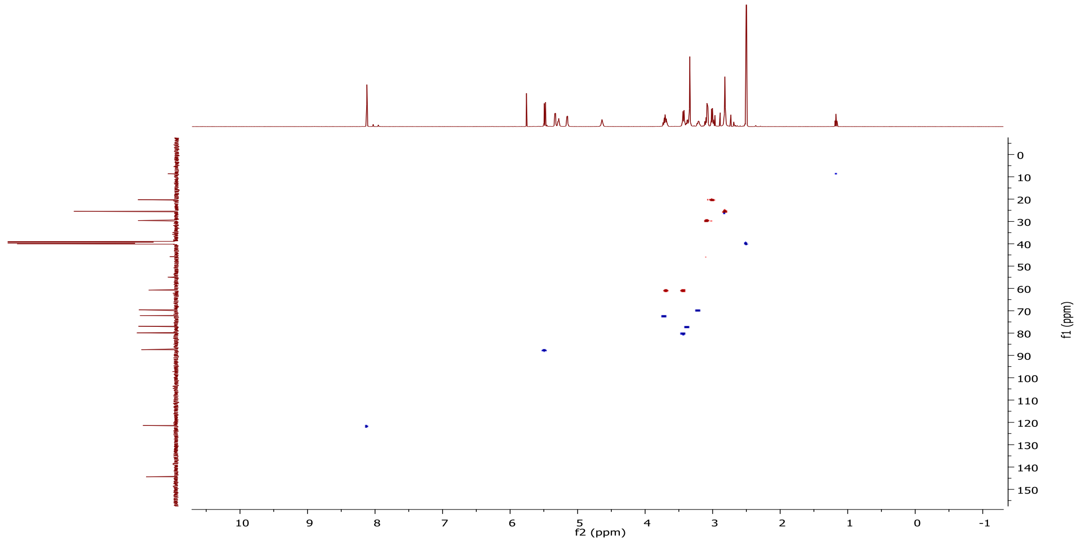

Figure S5d: HSQC NMR spectrum of **13** in DMSO

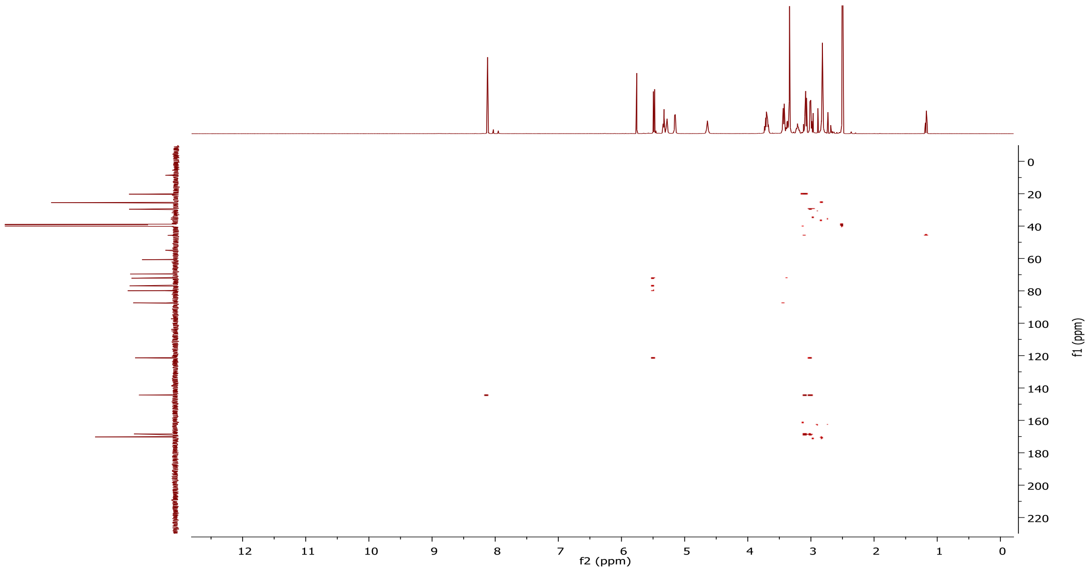

Figure S5e: HMBC NMR spectrum of **13** in DMSO

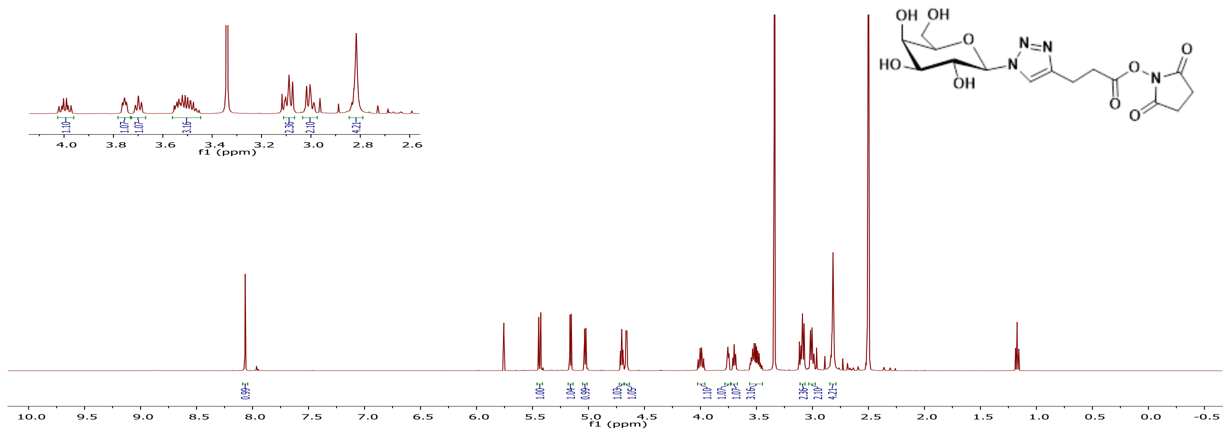

Figure S6a: ^{1}H NMR spectrum of **14** in DMSO

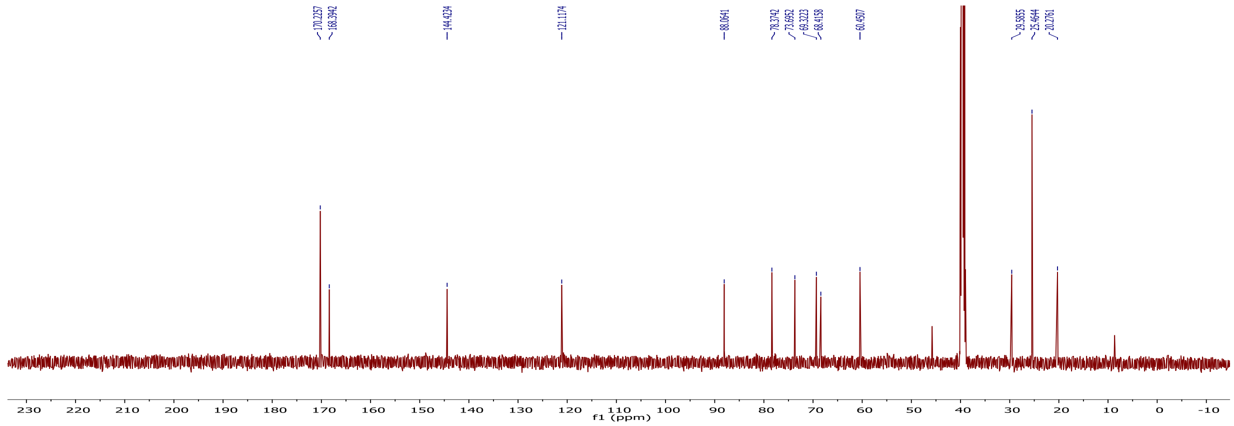

Figure S6b: ^{13}C NMR spectrum of **14** in DMSO

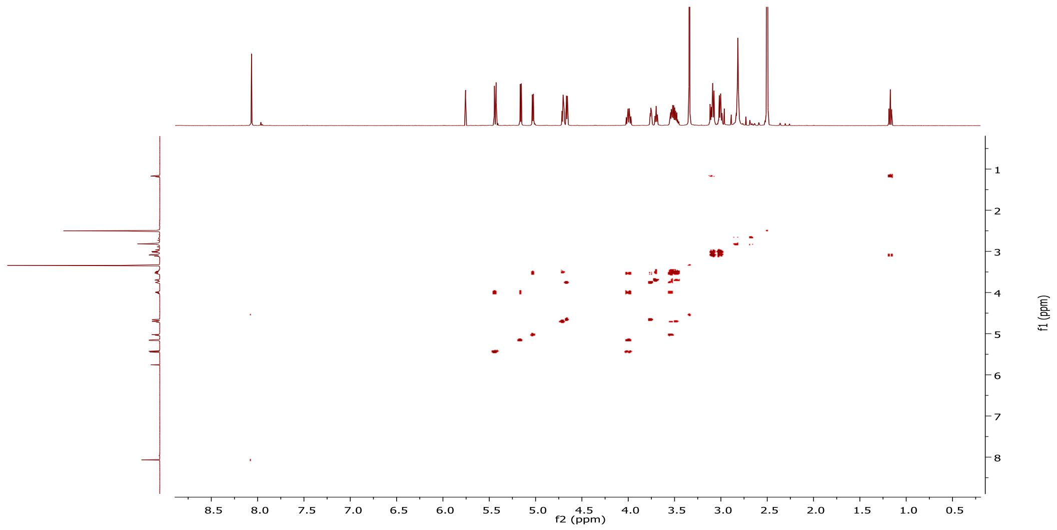

Figure S6c: COSY NMR spectrum of **14** in DMSO

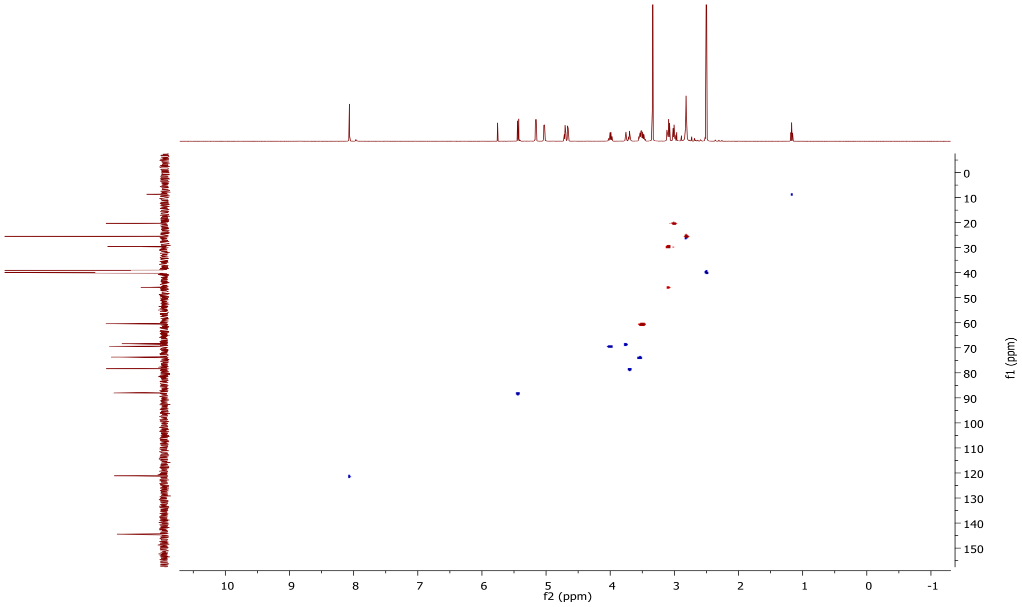

Figure S6d: HSQC NMR spectrum of **14** in DMSO

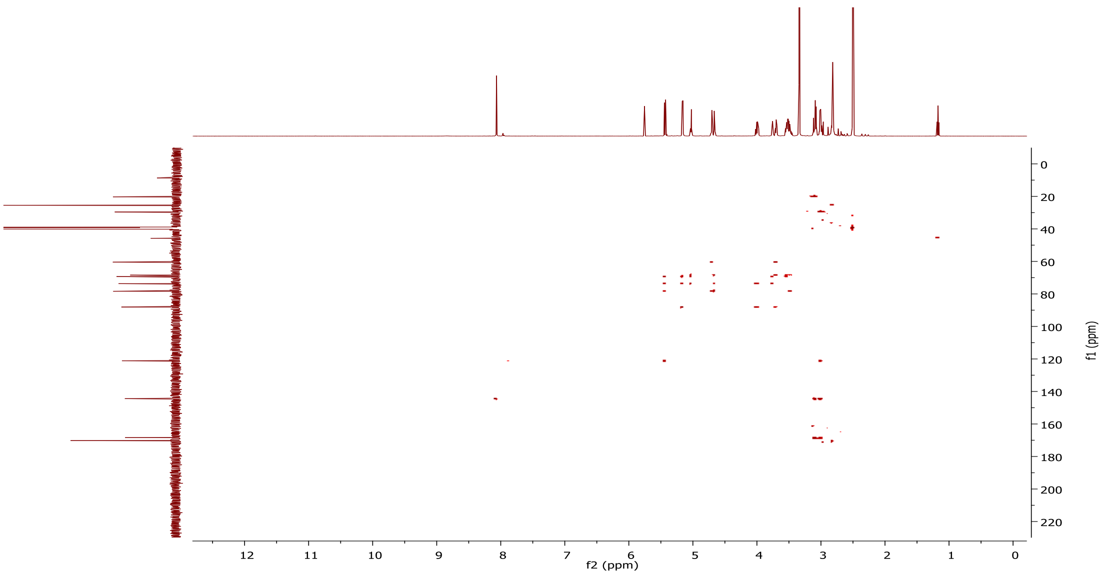

Figure S6e: HMBC NMR spectrum of **14** in DMSO

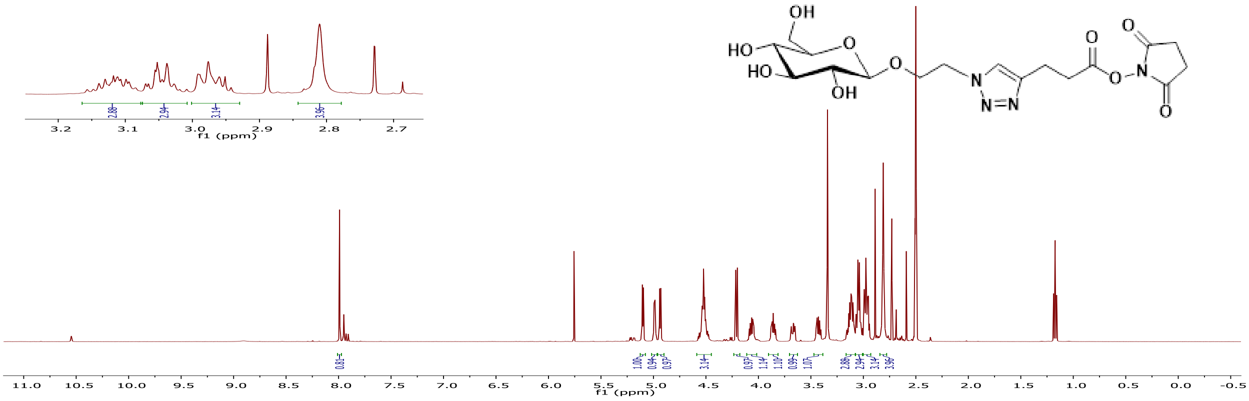

Figure S7a: ^{1}H NMR spectrum of **23** in DMSO

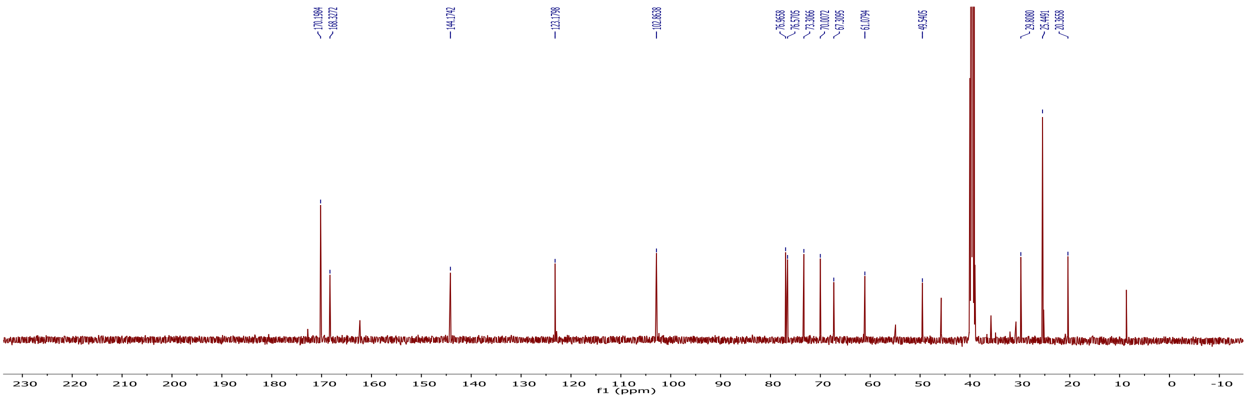

Figure S7b: ^{13}C NMR spectrum of **23** in DMSO

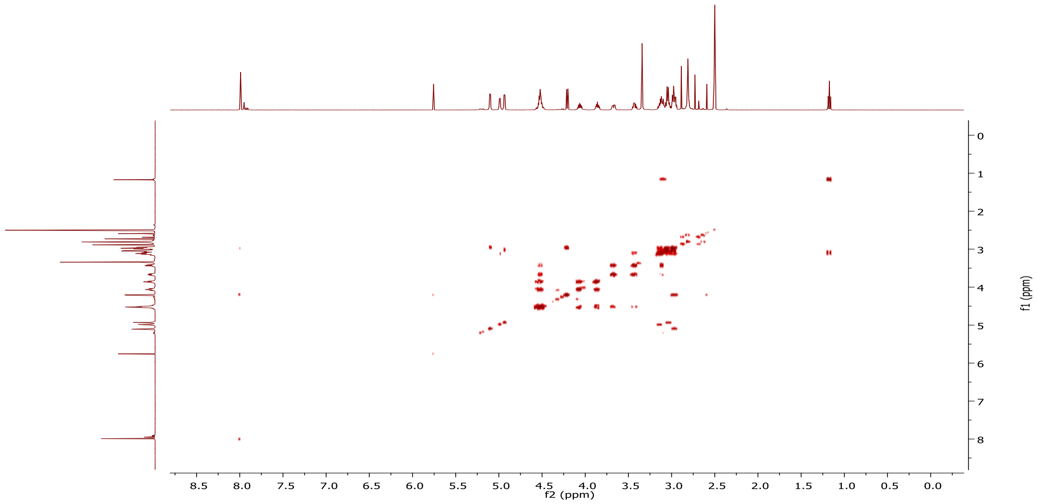

Figure S7c: COSY NMR spectrum of **23** in DMSO

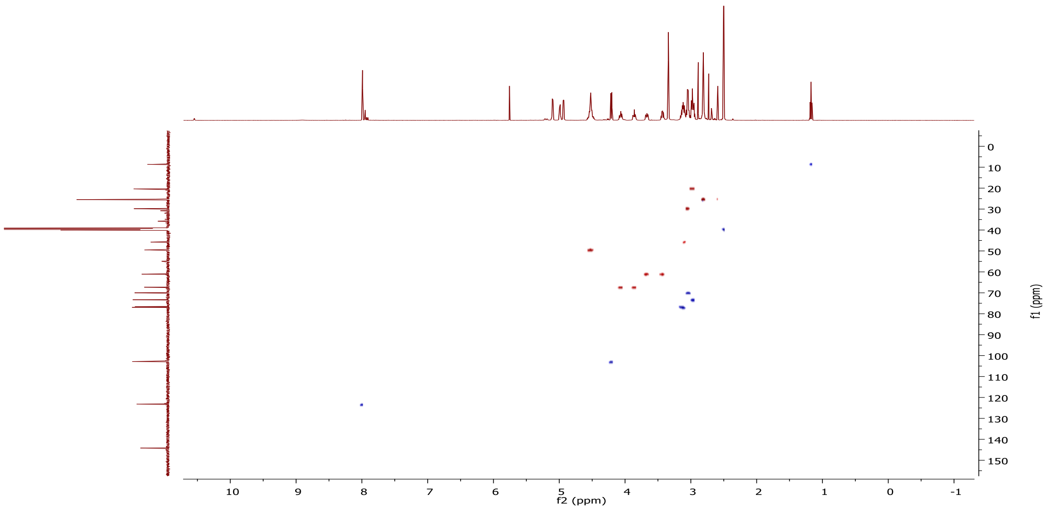

Figure S7d: HSQC NMR spectrum of **23** in DMSO

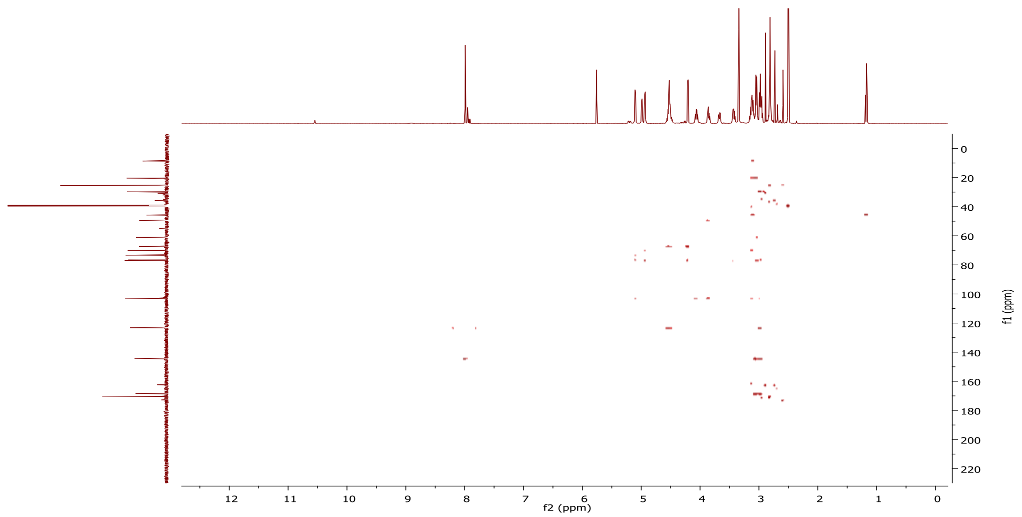

Figure S7e: HMBC NMR spectrum of **23** in DMSO

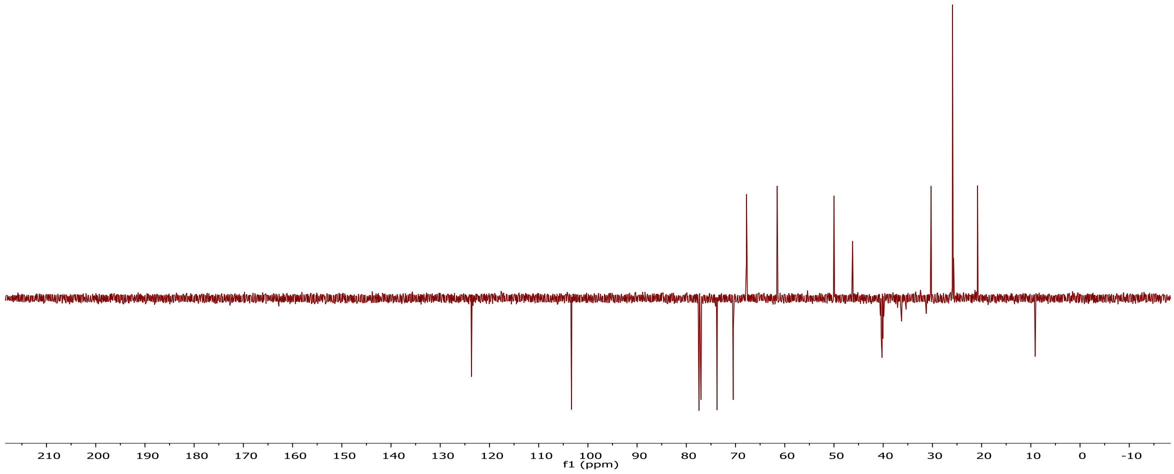

Figure S7f: DEPT-135 NMR spectrum of **23** in DMSO

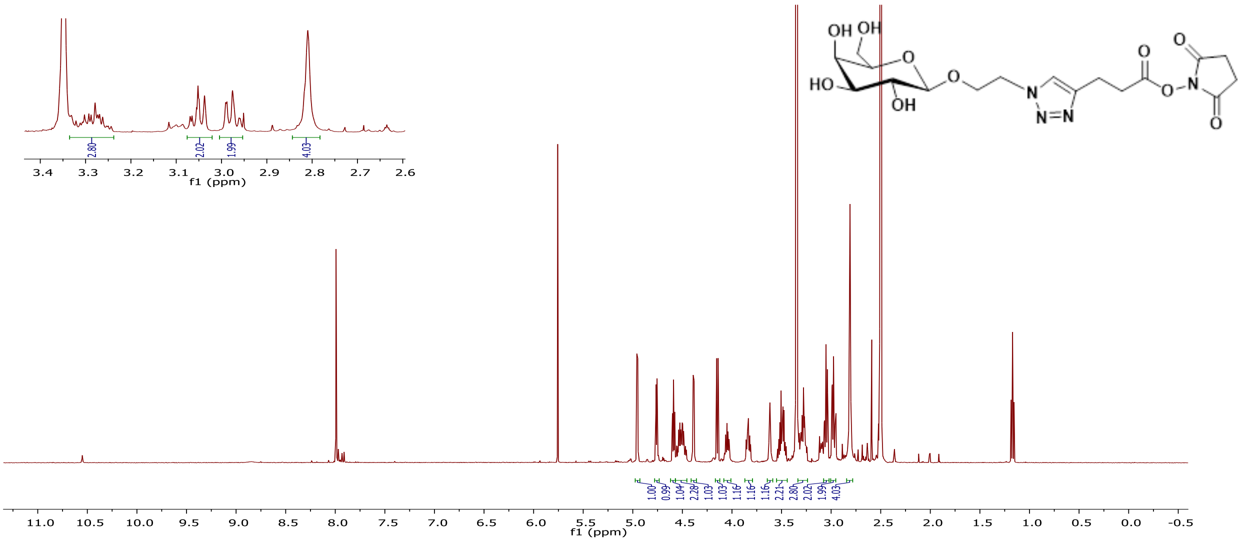

Figure S8a: ^{1}H NMR spectrum of **24** in DMSO

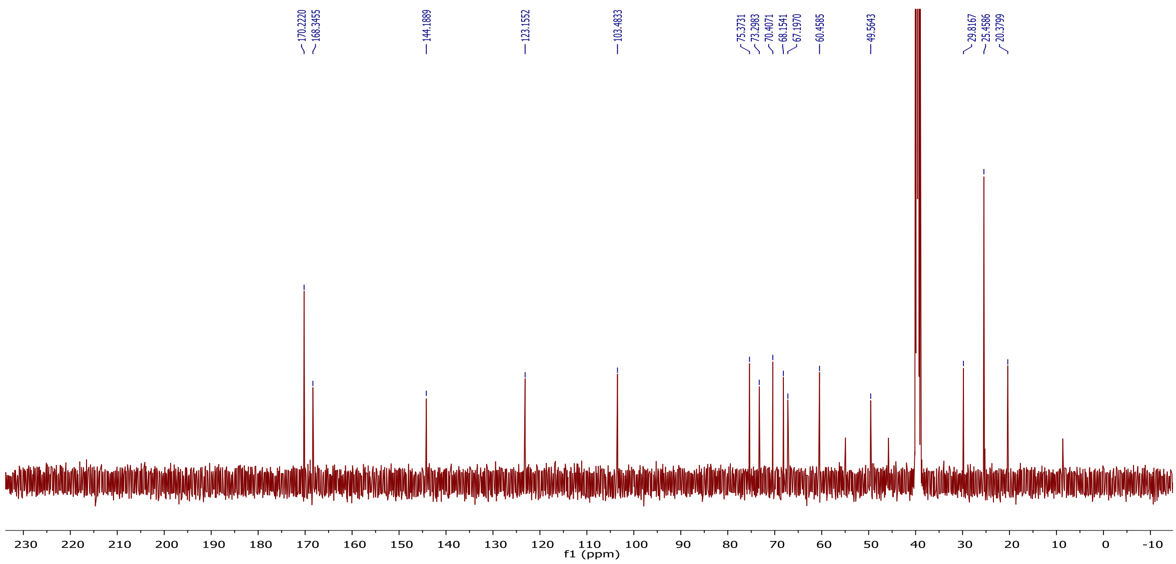

Figure S8b: ^{13}C NMR spectrum of **24** in DMSO

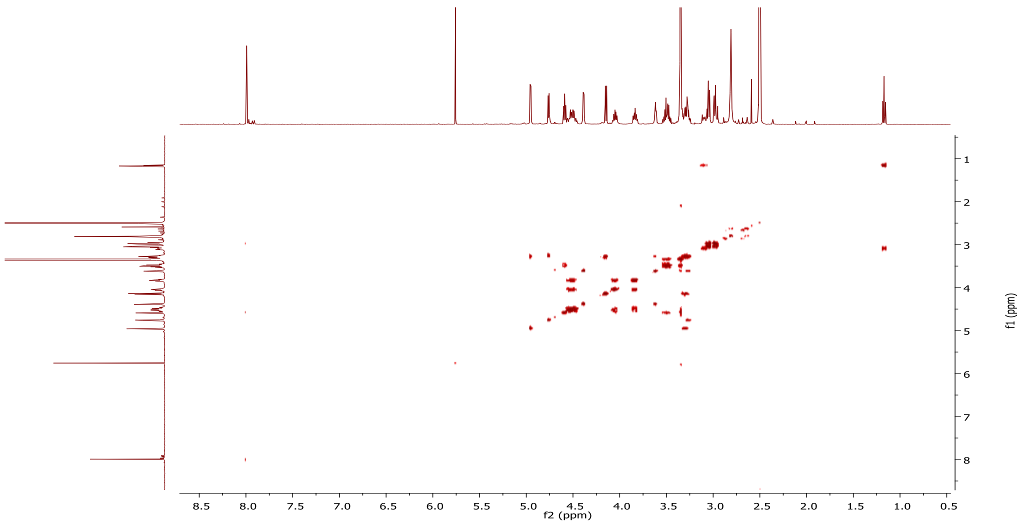

Figure S8c: COSY NMR spectrum of **24** in DMSO

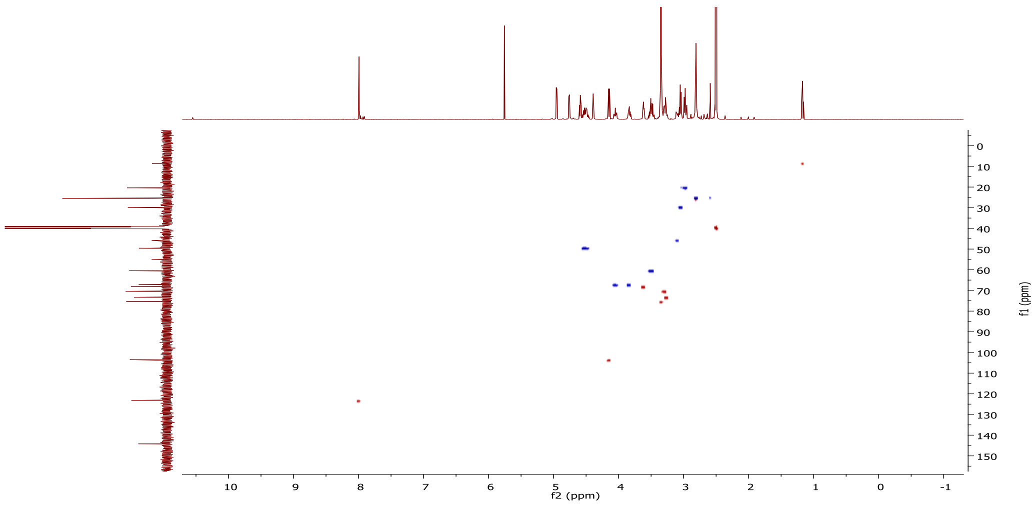

Figure S8d: HSQC NMR spectrum of **24** in DMSO

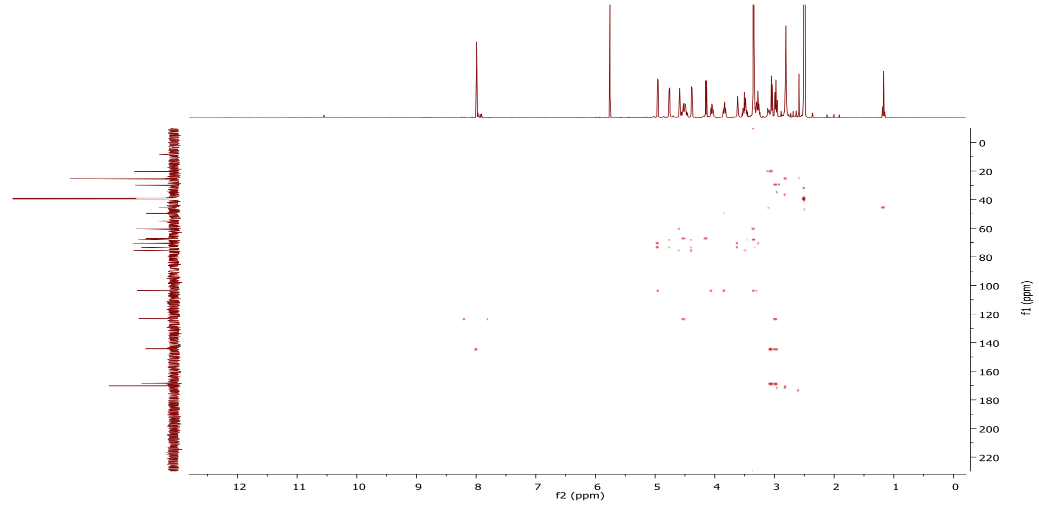

Figure S8e: HMBC NMR spectrum of **24** in DMSO

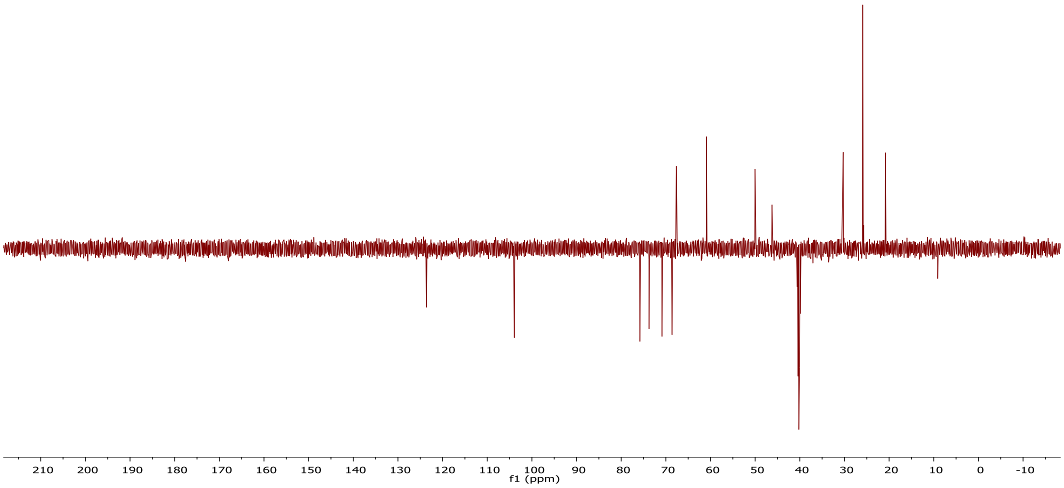

Figure S8f: DEPT-135 NMR spectrum of **24** in DMSO

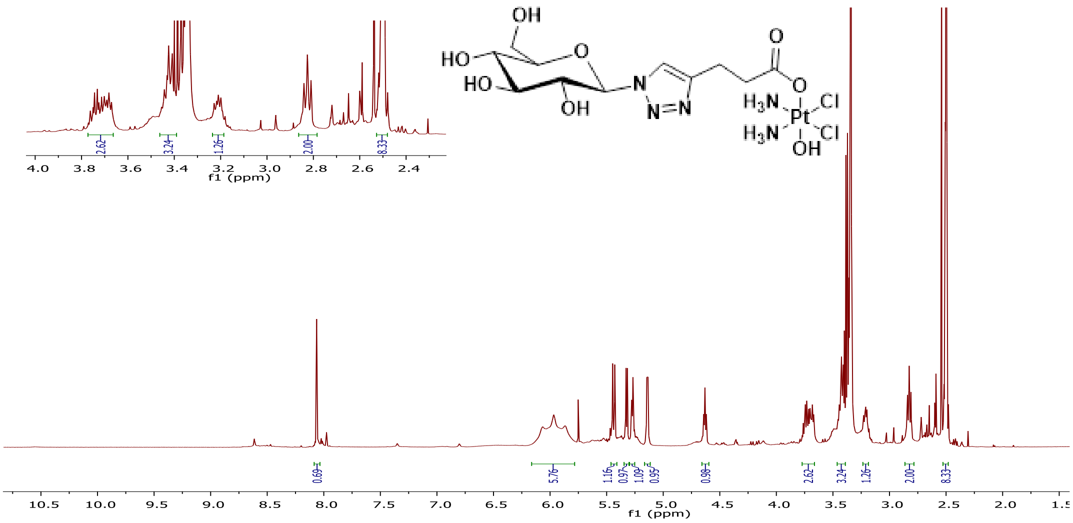

Figure S9a: ^{1}H NMR spectrum of **1** in DMSO

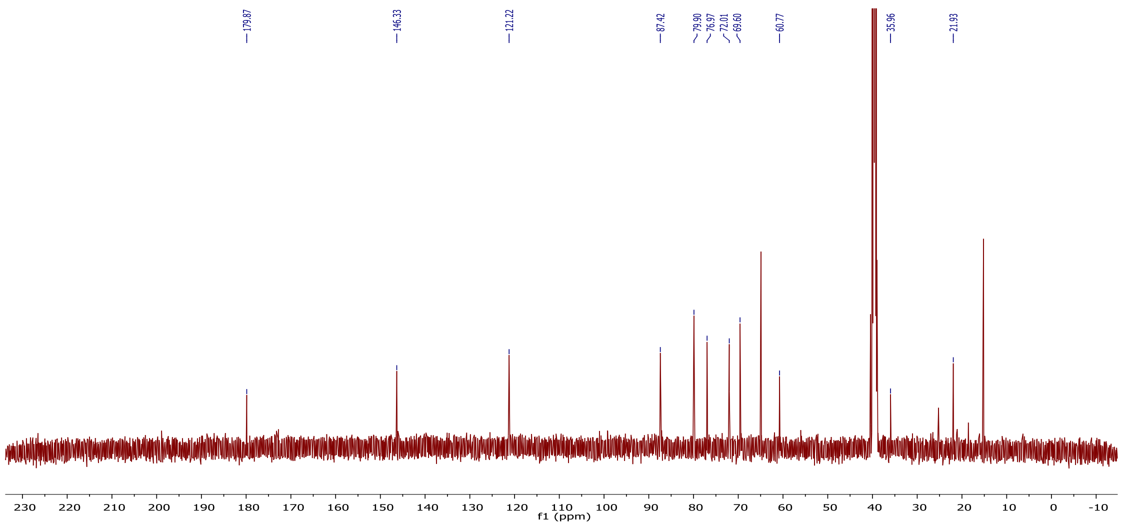

Figure S9b: ^{13}C NMR spectrum of **1** in DMSO

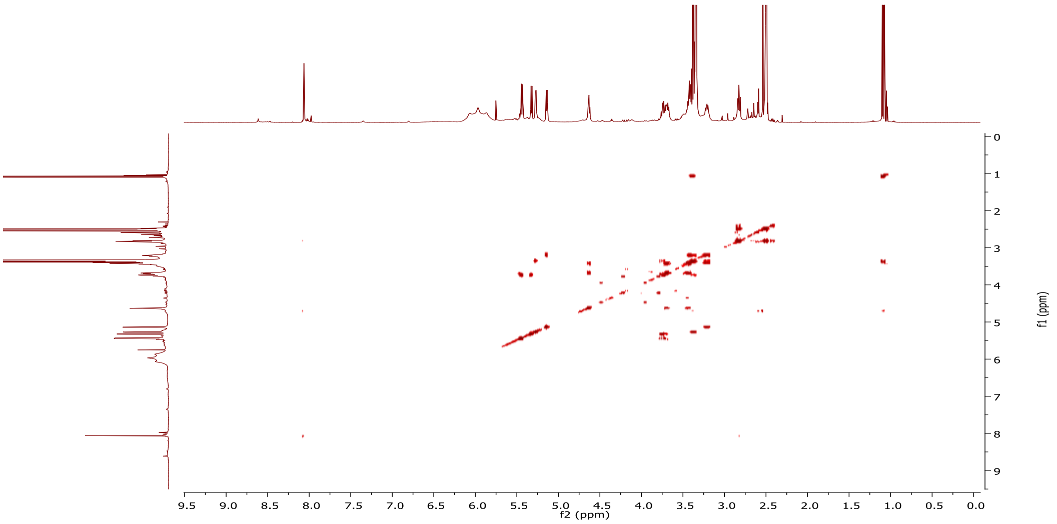

Figure S9c: COSY NMR spectrum of **1** in DMSO

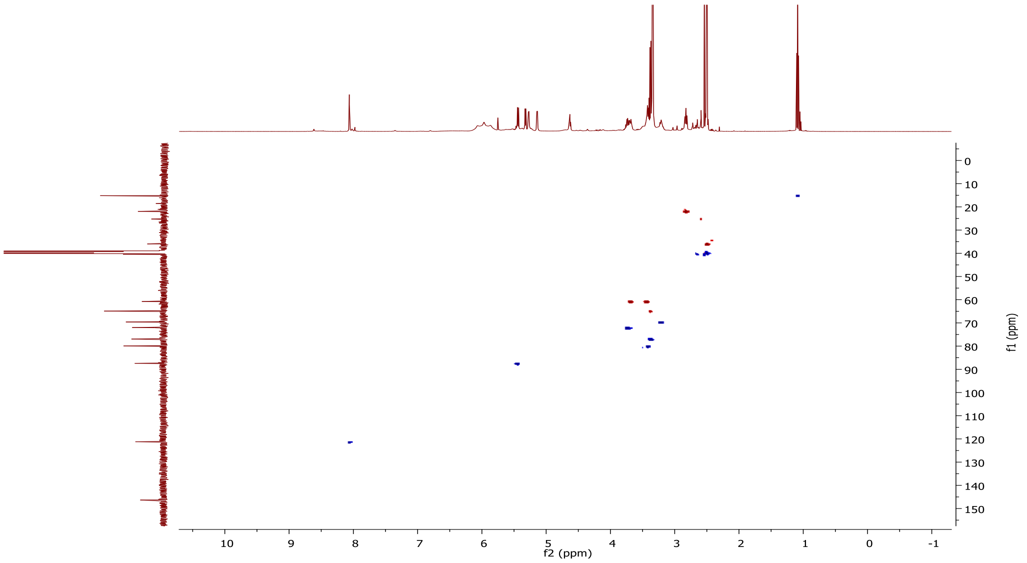

Figure S9d: HSQC NMR spectrum of **1** in DMSO

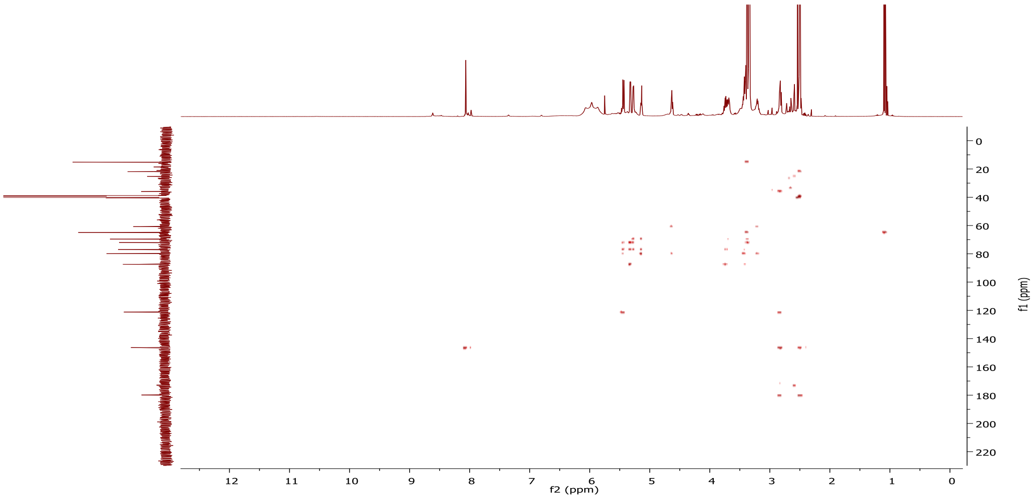

Figure S9e: HMBC NMR spectrum of **1** in DMSO

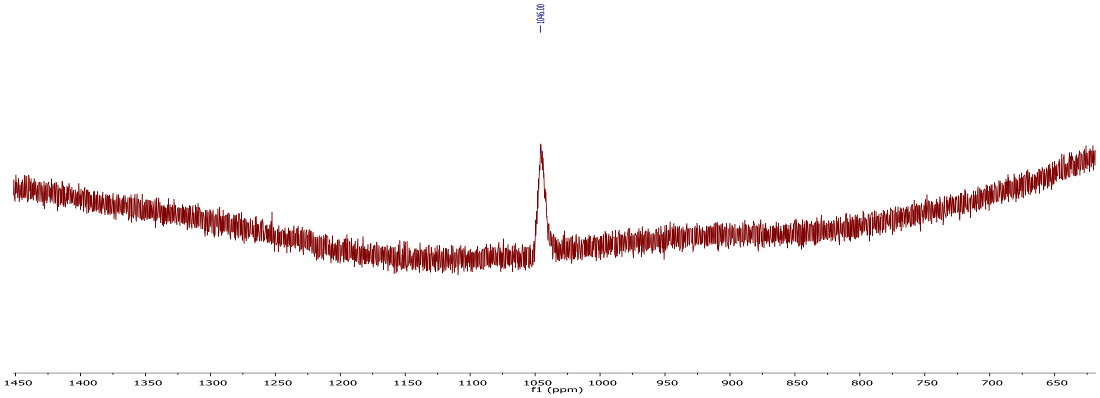

Figure S9f: ^{195}Pt NMR spectrum of **1** in DMSO

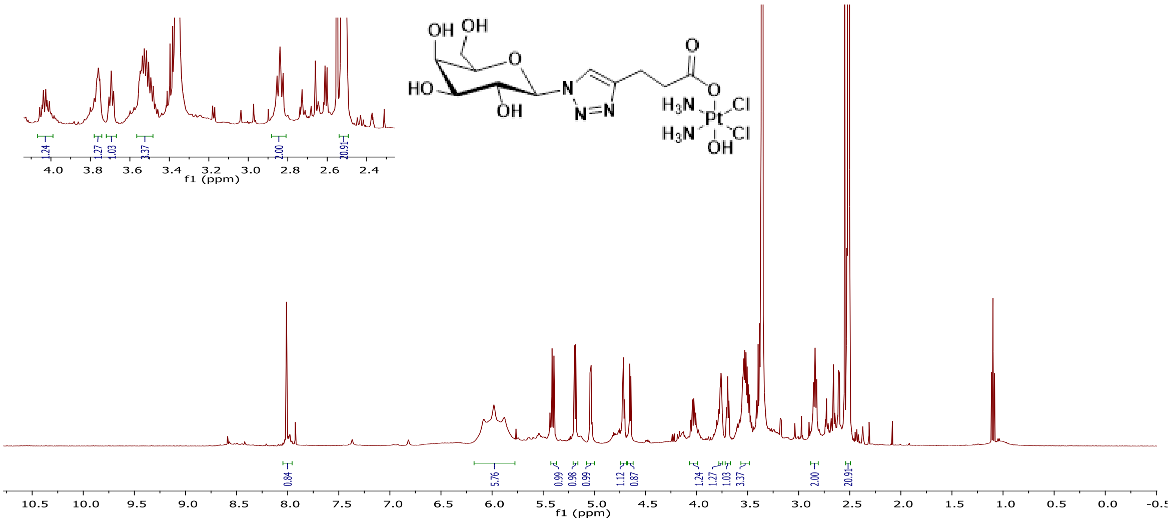

Figure S10a: ^{1}H NMR spectrum of **2** in DMSO

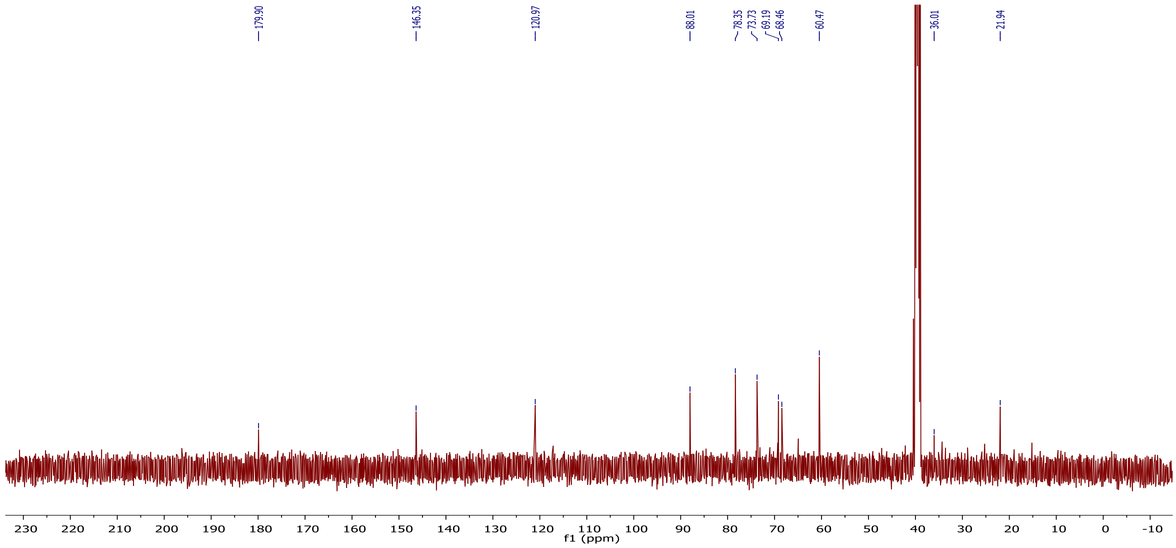

Figure S10b: ^{13}C NMR spectrum of **2** in DMSO

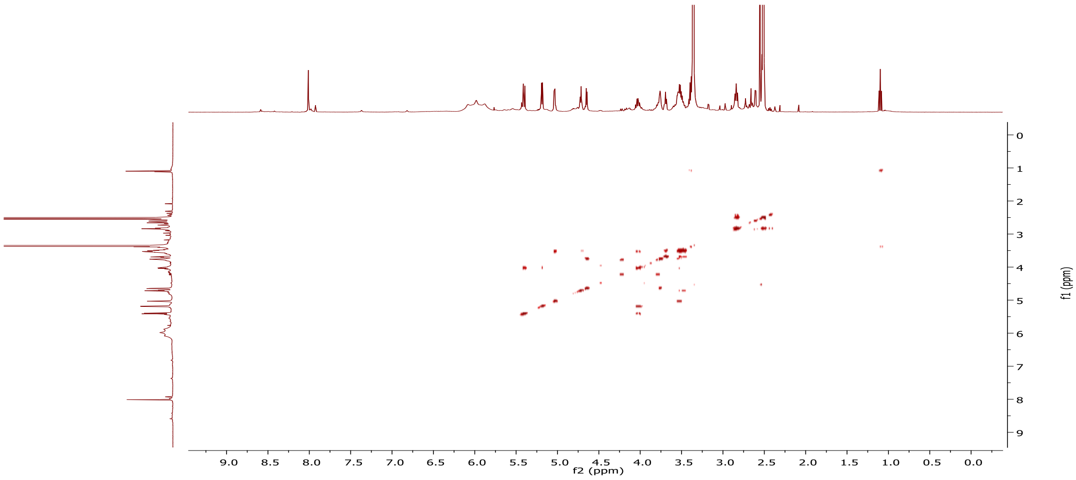

Figure S10c: COSY NMR spectrum of **2** in DMSO

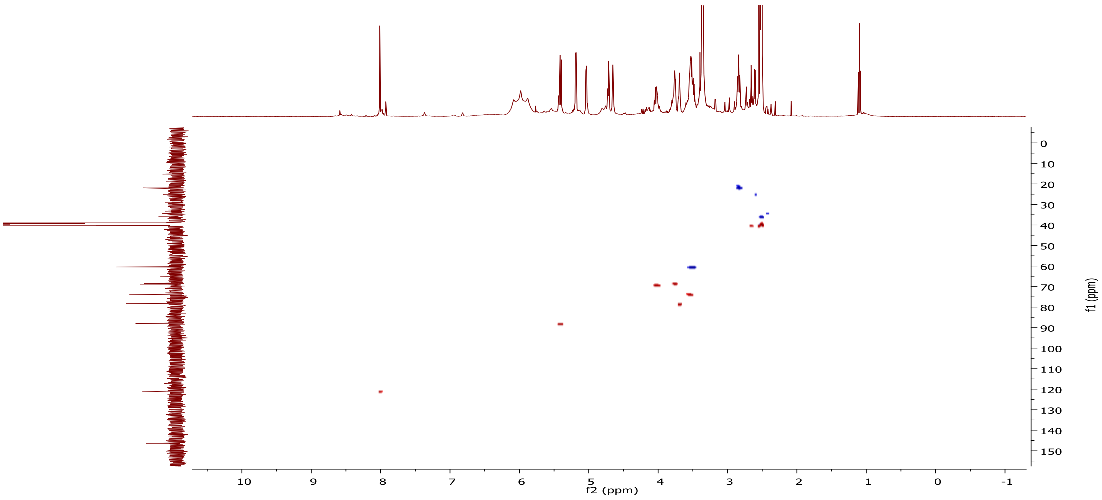

Figure S10d: HSQC NMR spectrum of **2** in DMSO

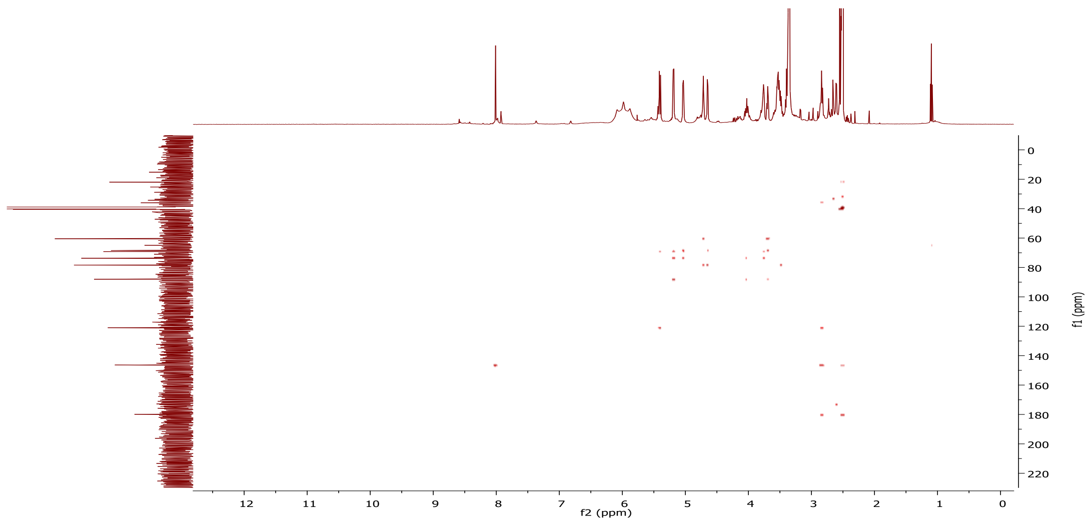

Figure S10e: HMBC NMR spectrum of **2** in DMSO

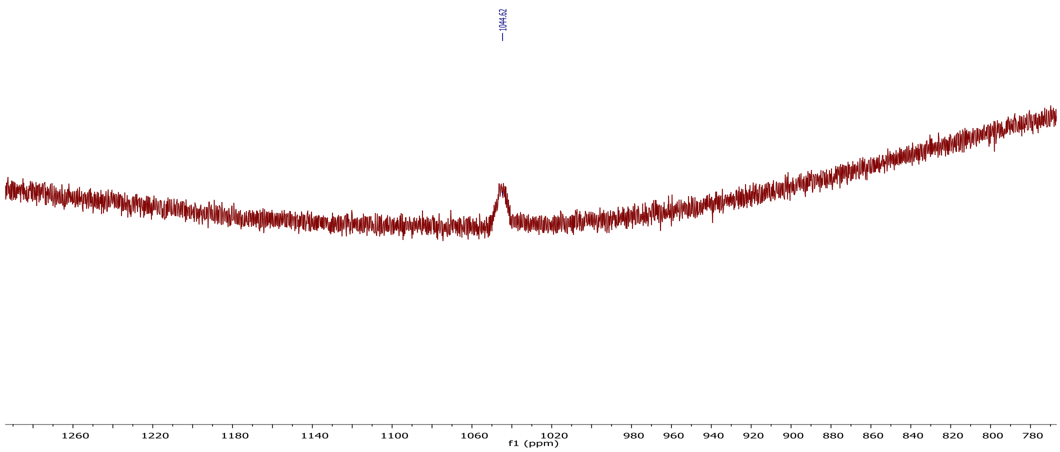

Figure S10f: ^{195}Pt NMR spectrum of **2** in DMSO

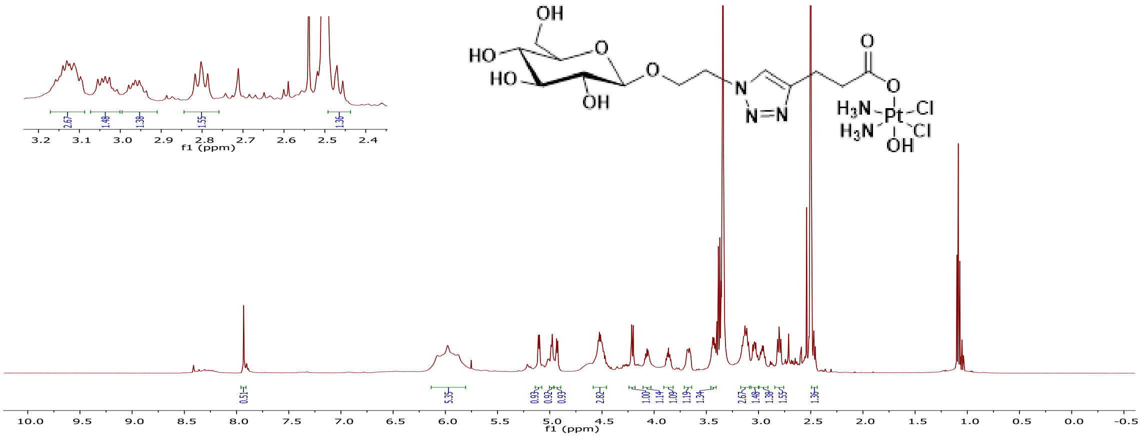

Figure S11a: ^{1}H NMR spectrum of **3** in DMSO

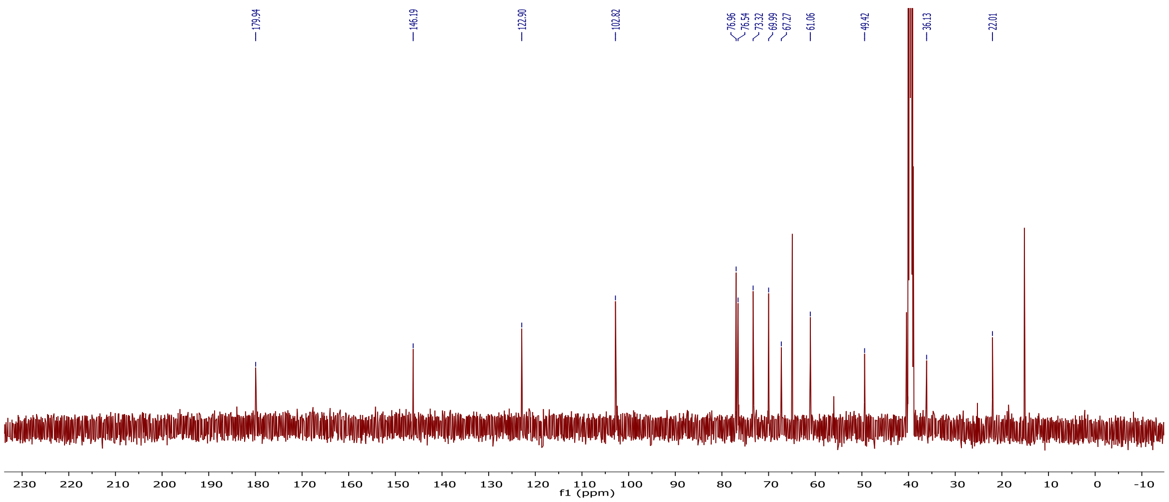

Figure S11b: ^{13}C NMR spectrum of **3** in DMSO

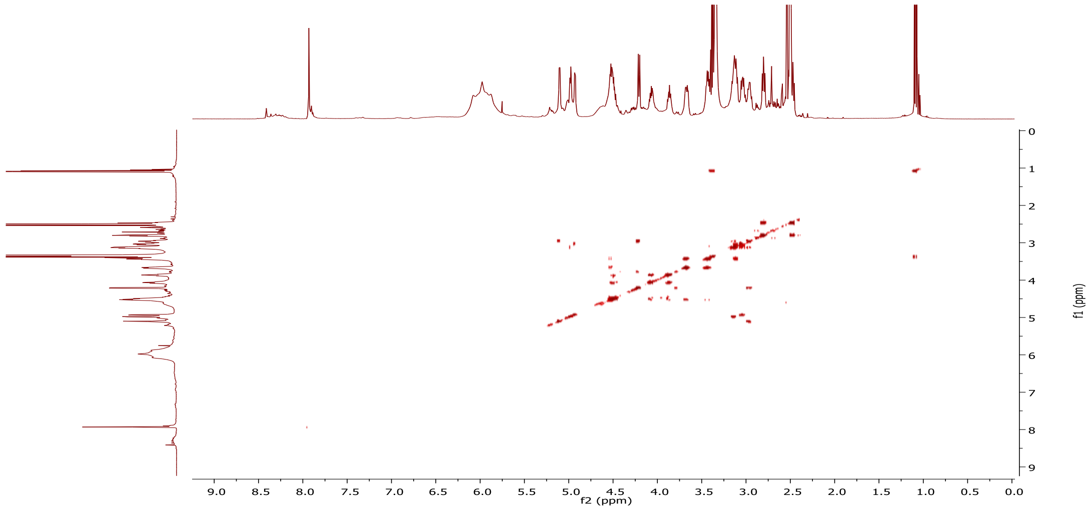

Figure S11c: COSY NMR spectrum of **3** in DMSO

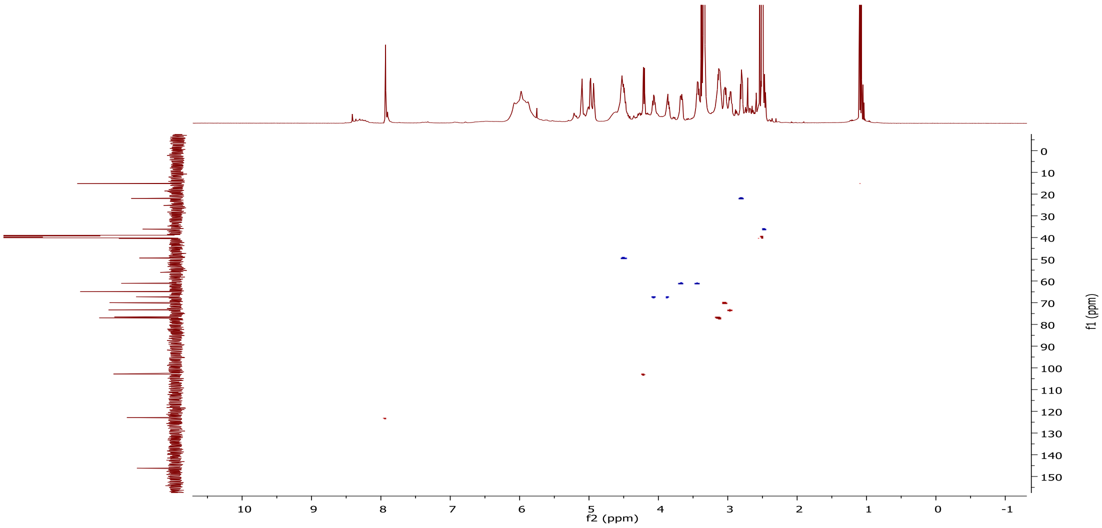

Figure S11d: HSQC NMR spectrum of **3** in DMSO

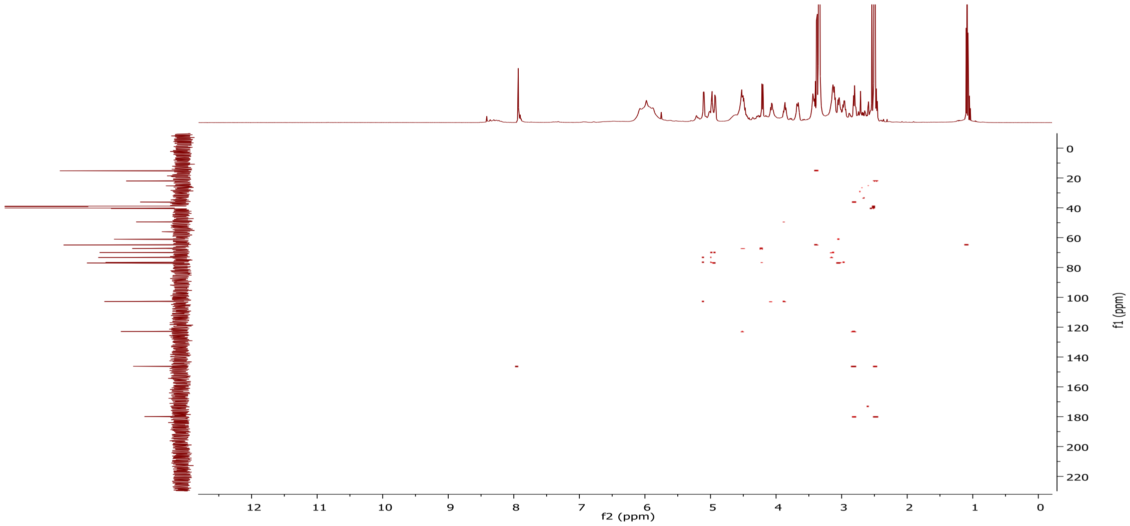

Figure S11e: HMBC NMR spectrum of **3** in DMSO

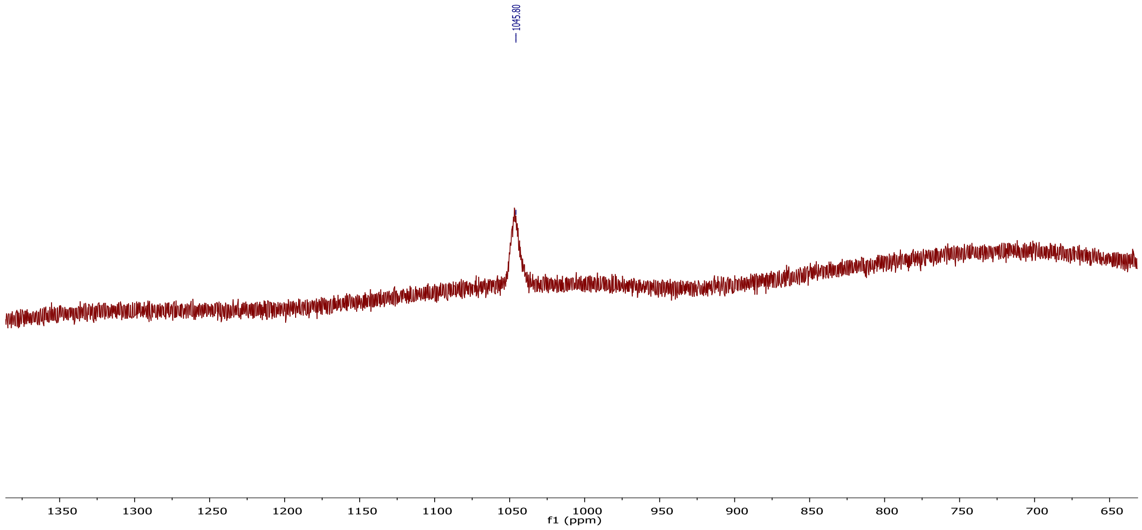

Figure S11f: ^{195}Pt NMR spectrum of **3** in DMSO

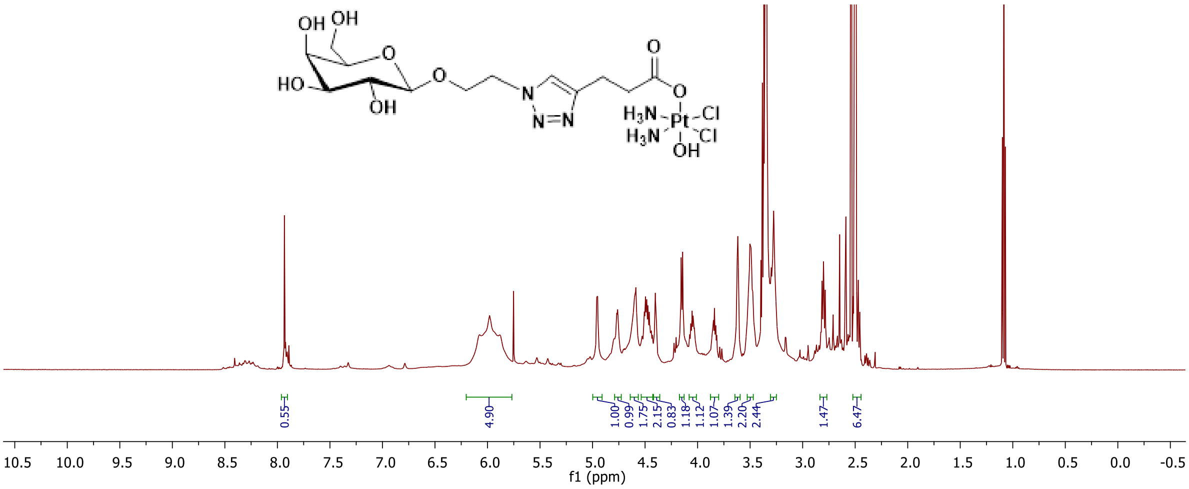

Figure S12a: ^{1}H NMR spectrum of **4** in DMSO

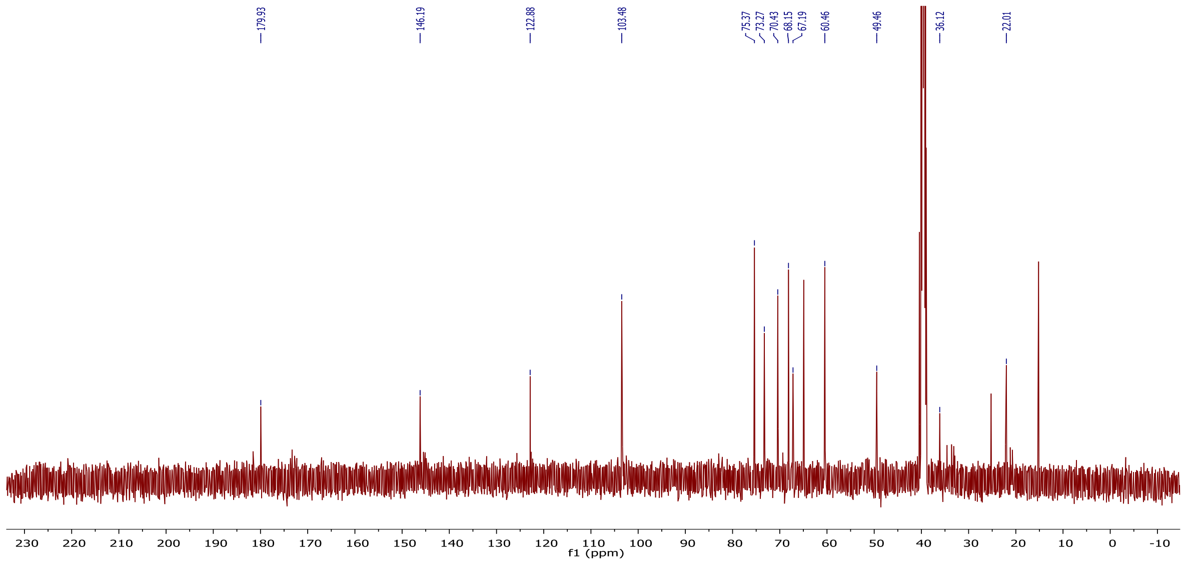

Figure S12b: ^{13}C NMR spectrum of **4** in DMSO

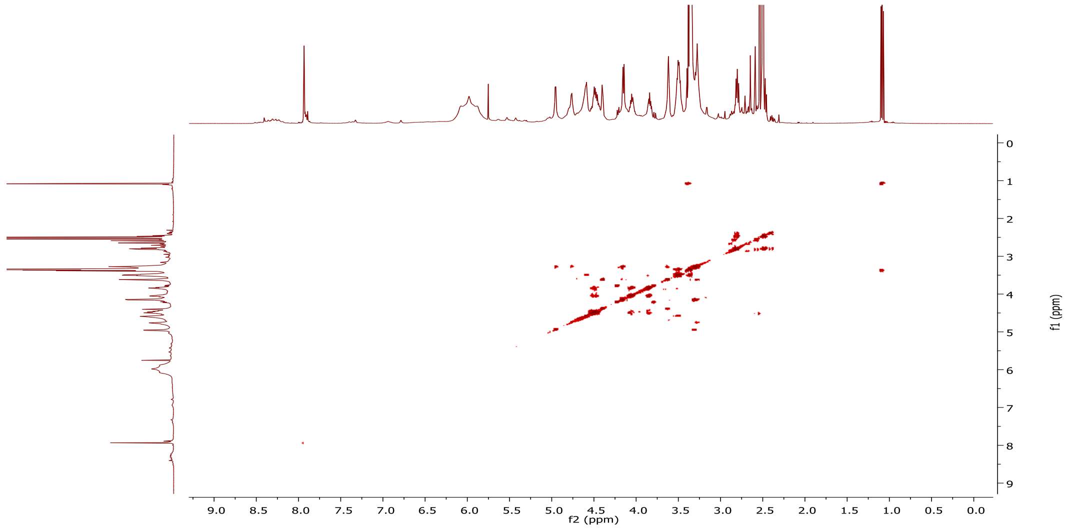

Figure S12c: COSY NMR spectrum of **4** in DMSO

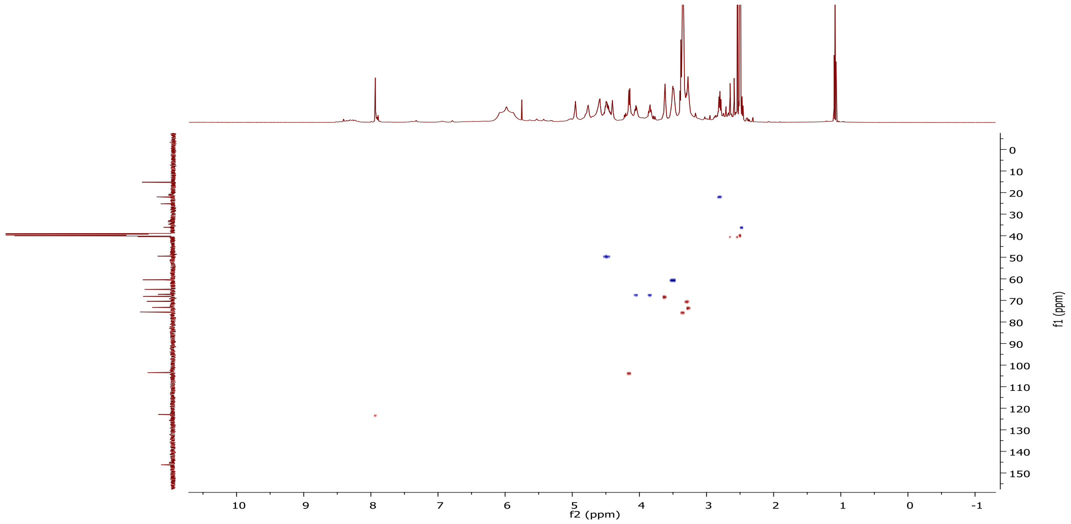

Figure S12d: HSQC NMR spectrum of **4** in DMSO

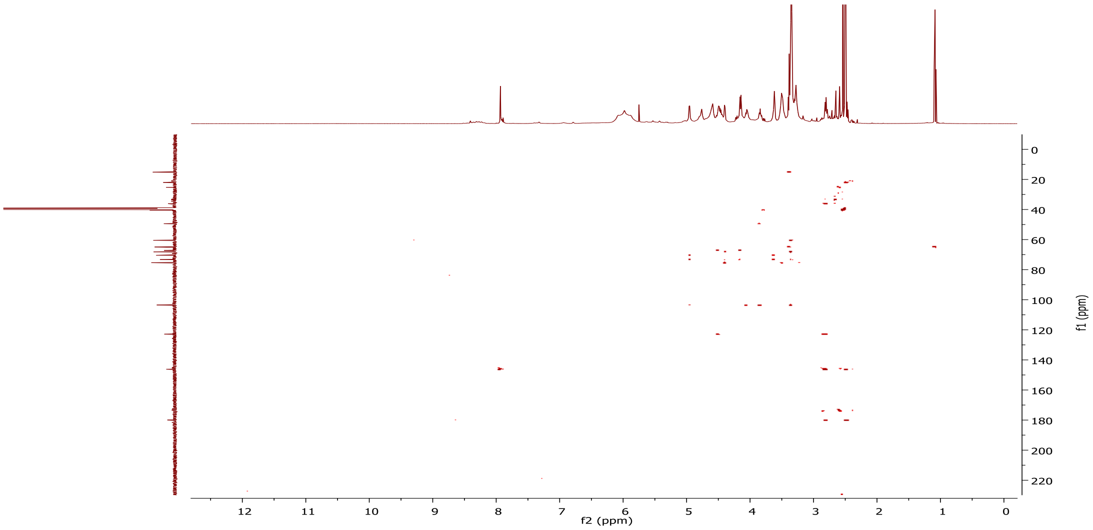

Figure S12e: HMBC NMR spectrum of **4** in DMSO

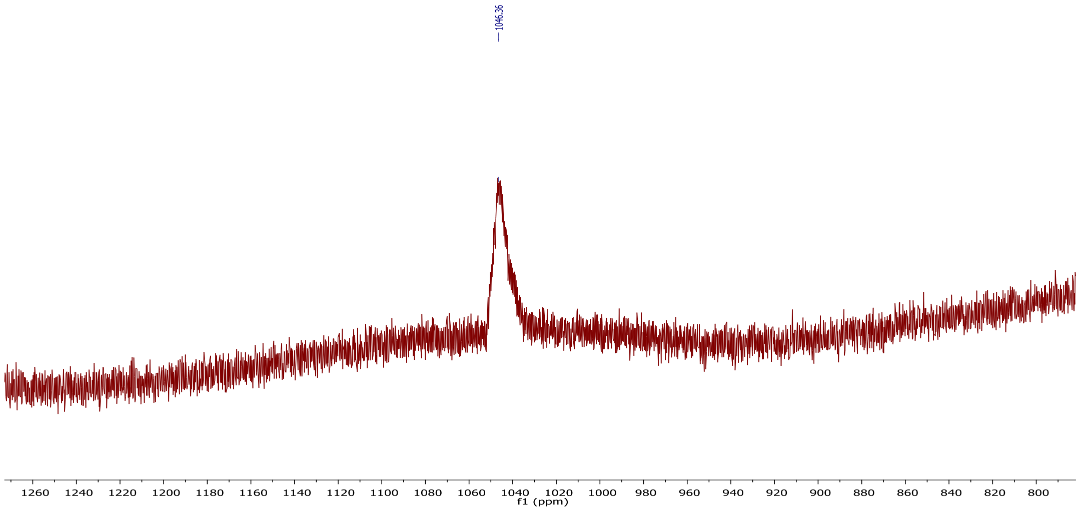

Figure S12f: ^{195}Pt NMR spectrum of **4** in DMSO

HR-MS Spectra

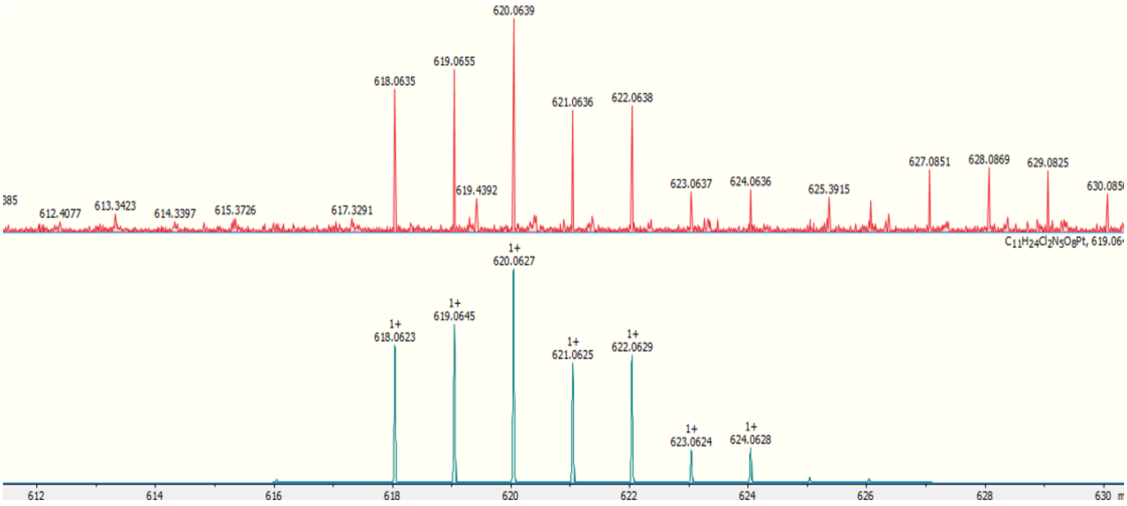

Figure S13: HR-MS spectrum of **1**

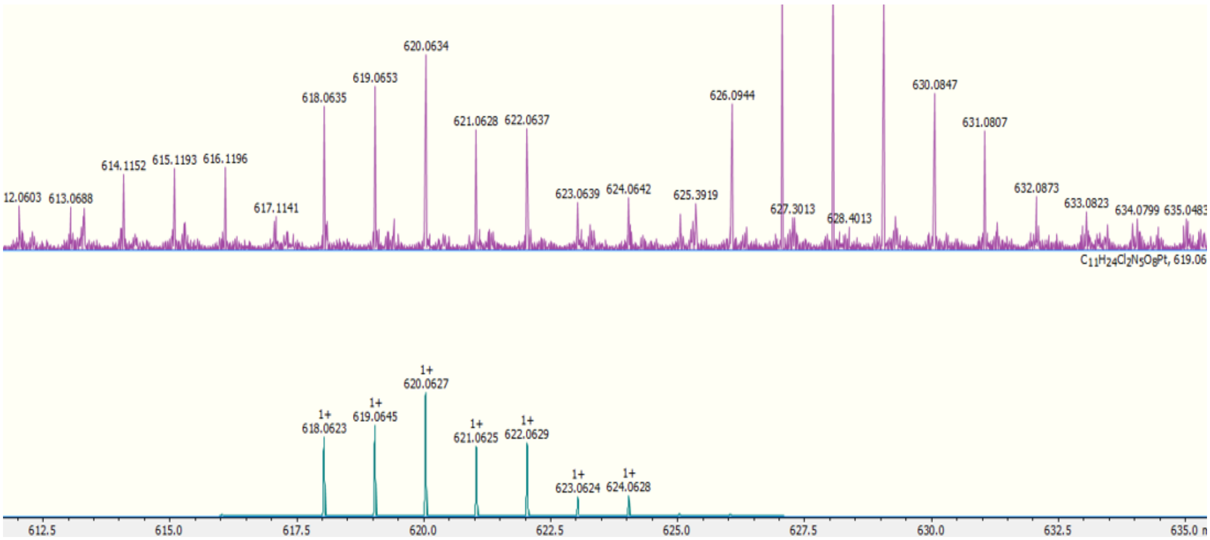

Figure S14: HR-MS spectrum of **2**

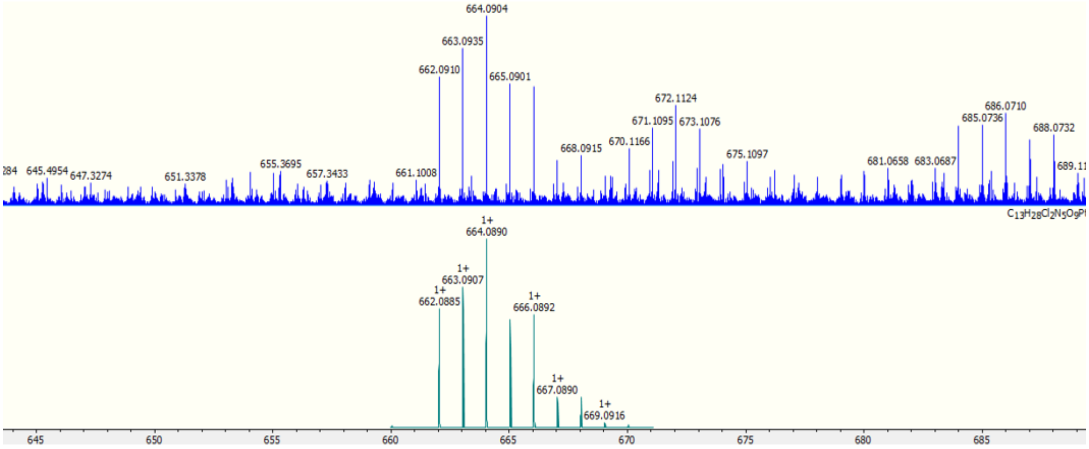

Figure S15: HR-MS spectrum of **3**

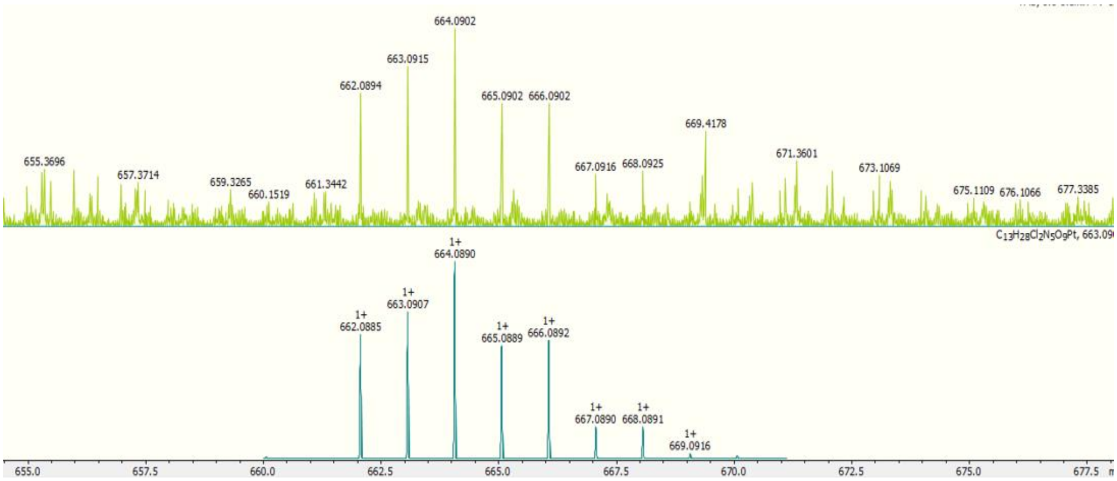

Figure S16: HR-MS spectrum of **4**

Stability

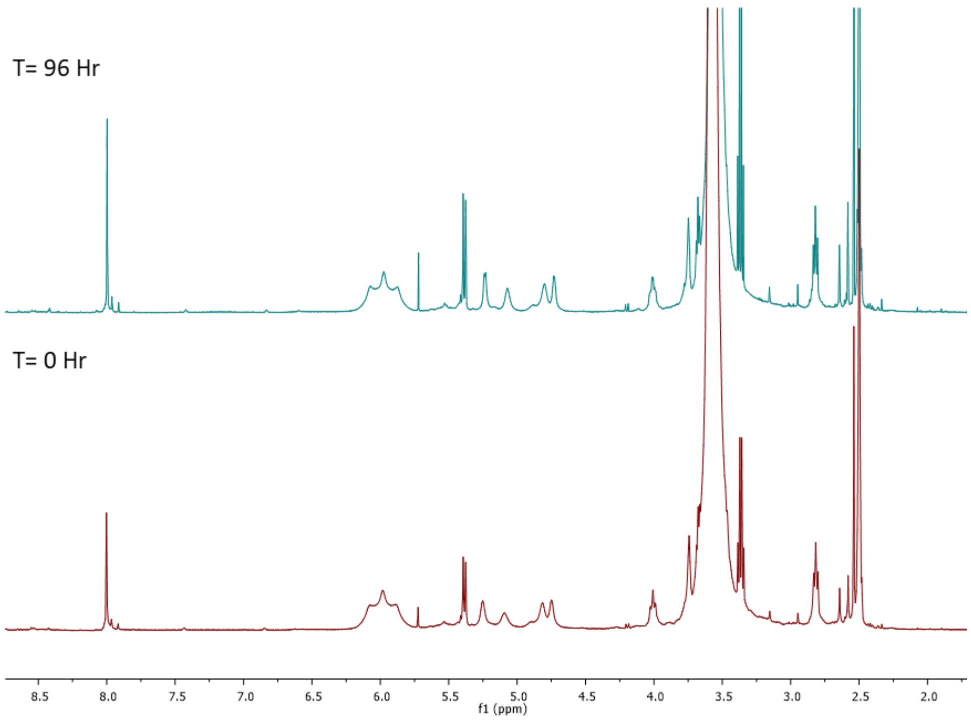

Figure S17a: ^{1}H NMR of **1** in DMSO and PBS buffer at 0 and 96 hours.

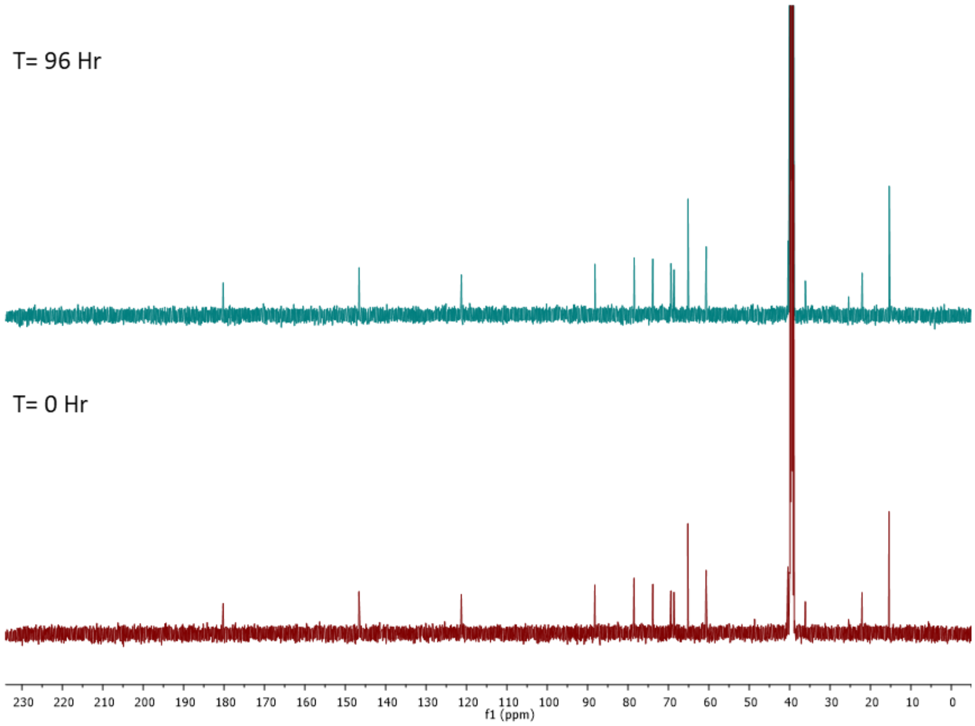

Figure S17b: ^{13}C NMR of **1** in DMSO and PBS buffer at 0 and 96 hours.

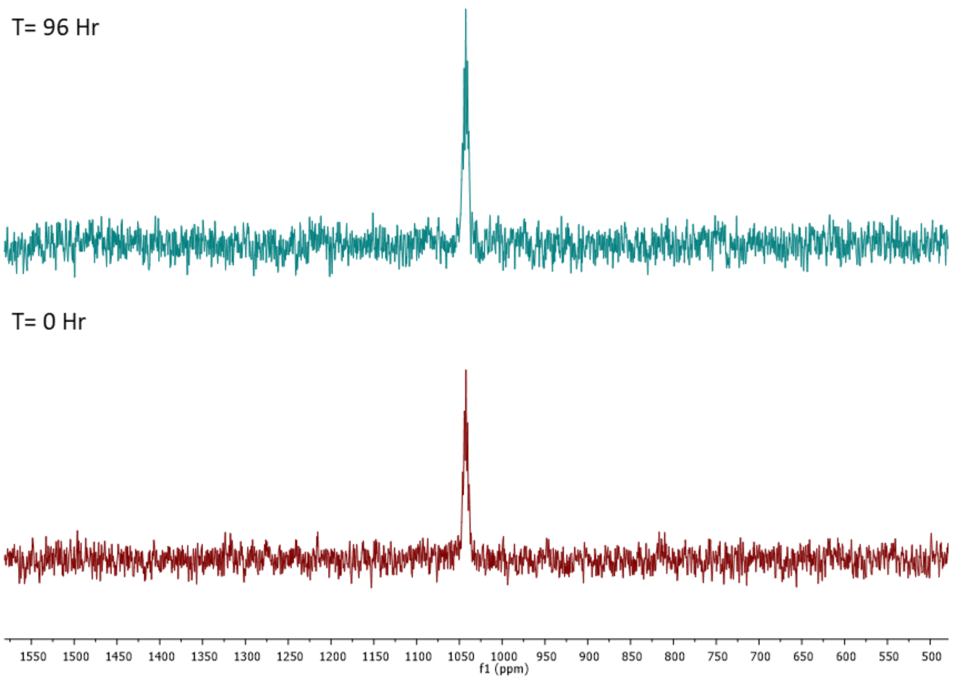

Figure S17c: ^{195}Pt NMR of **1** in DMSO and PBS buffer at 0 and 96 hours

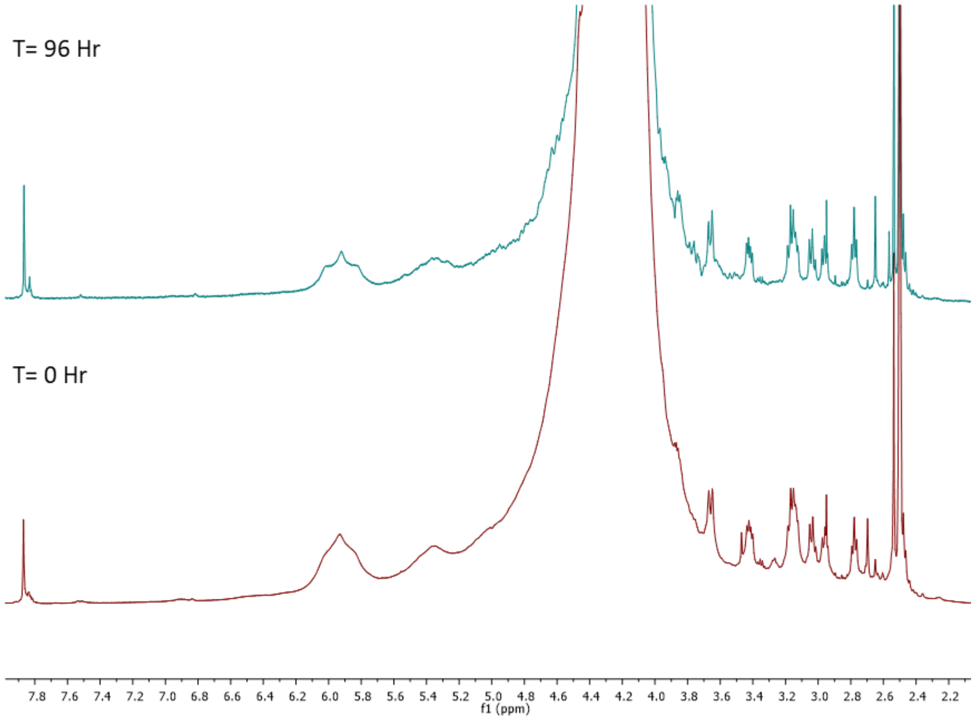

Figure S17d: ^{1}H NMR of **3** in DMSO and PBS buffer at 0 and 96 hours

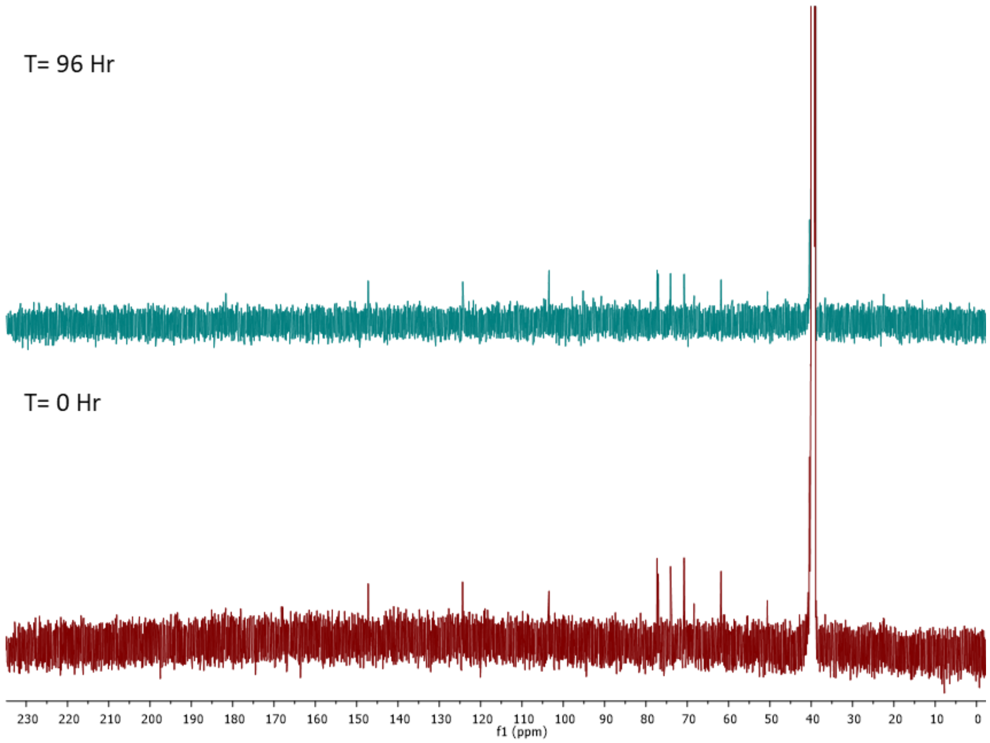

Figure S17e: ^{13}C NMR of **3** in DMSO and PBS buffer at 0 and 96 hours.

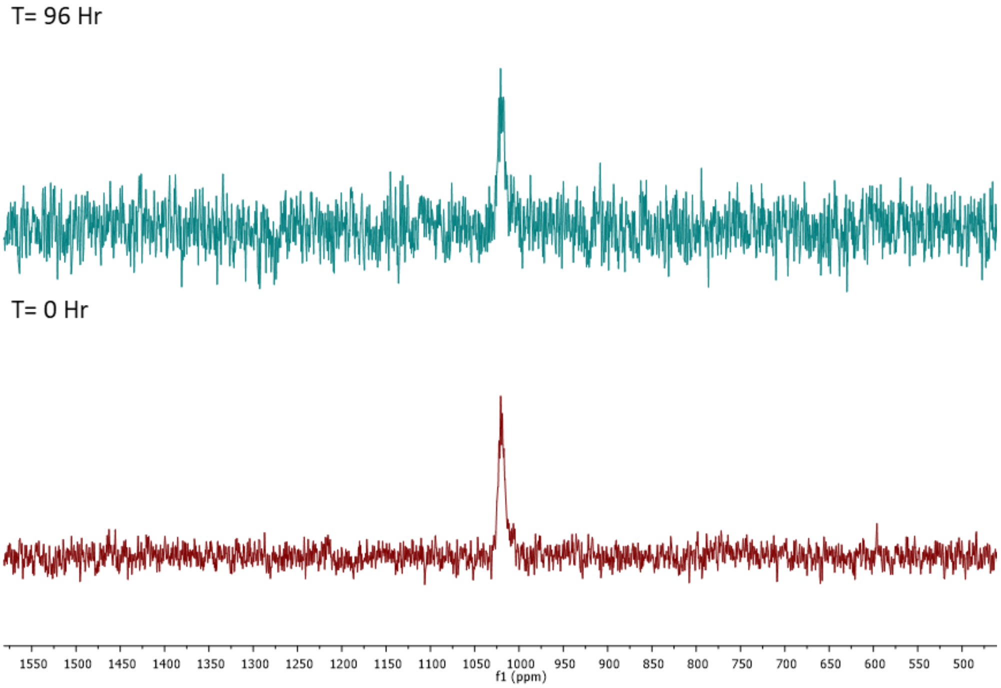

Figure S17f: ^{195}Pt NMR of **3** in DMSO and PBS at 0 and 96 hours.

Cyclic Voltammetry

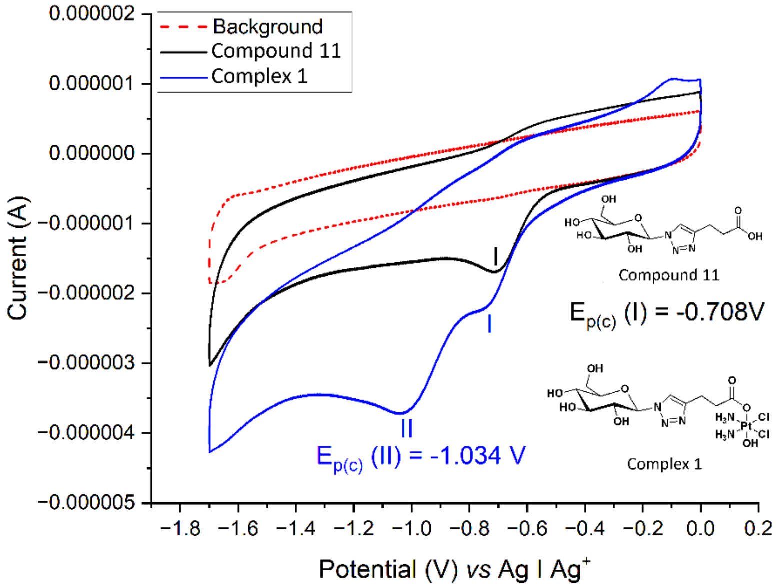

Figure S18: Cyclic voltammogram of Complex **1** and ligand **11**

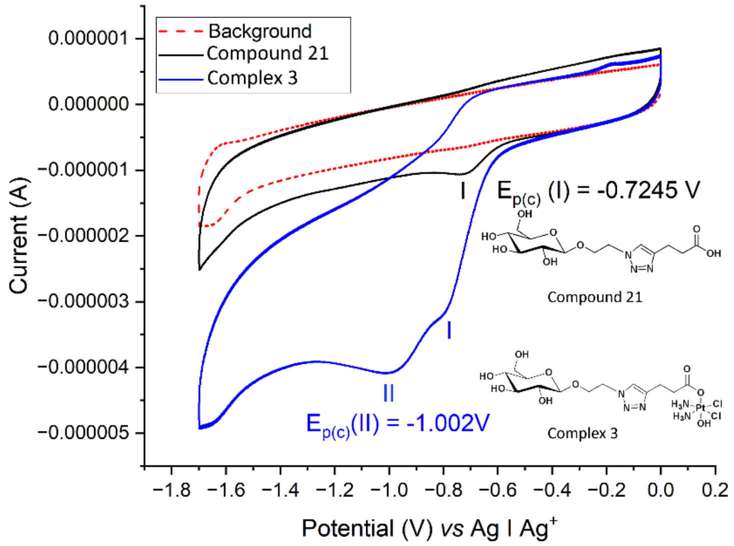

Figure S19: Cyclic voltammogram of Complex **3** and ligand **21**

Reduction Study

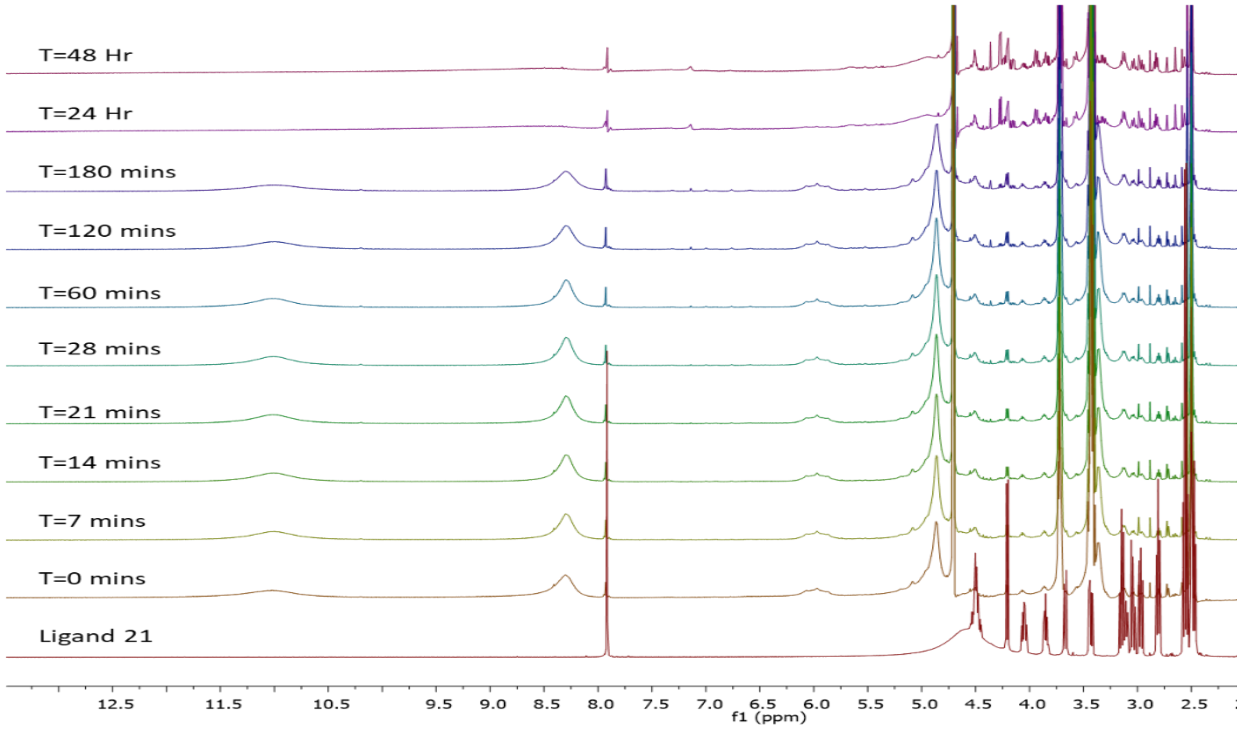

Figure S20: ^{1}H-NMR spectra of the complex **3** with addition of 10 eq. of ascorbic acid. NMR spectra were collected every 7minutes for 30 minutes and then every hour for 3 hours and finally left to reduce for 2 days.

Biological Figures

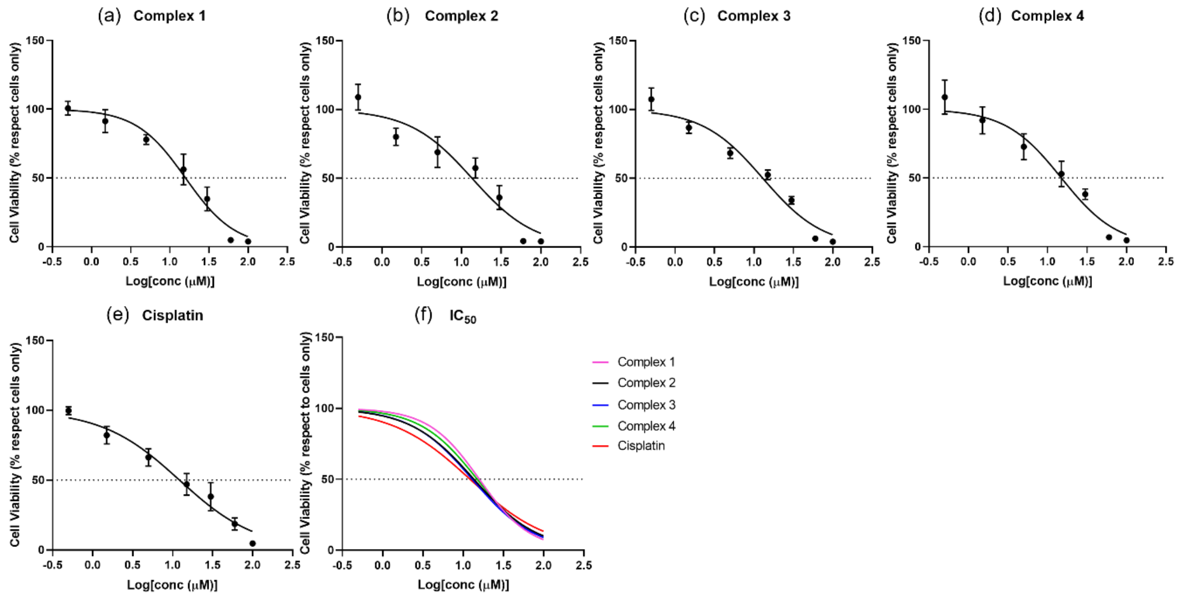

Figure S21: 2D *in vitro* screening of complexes 1-4 on MG63 cell line by MTT assay and IC_{50} (μM) values. Dose-response curves used to generate IC_{50} (μM) for complexes 1 (a), 2 (b), 3 (c) and 4 (d), and for cisplatin (e) inhibitor activity on cell viability of MG63 cell line. The Log[concentration] in μM and the normalized response (%) of survival fraction of cells are reported on X and Y asses, respectively. For each complex, the curve interpolation with 50% survival cells is highlighted in Y dotted line and correspond to its $LogIC_{50}$. A comparison of all dose-response curves is reported (f).

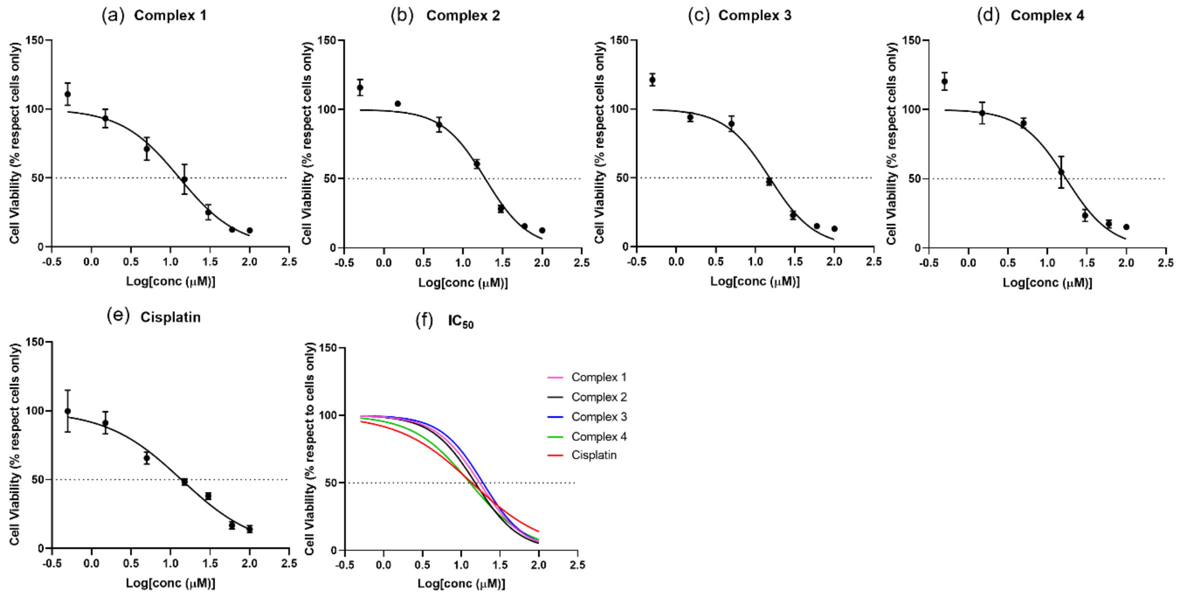

Figure S22: 2D *in vitro* screening of complexes 1-4 on SAOS-2 cell line by MTT assay and IC_{50} (μM) values. Dose-response curves used to generate IC_{50} (μM) for complexes 1 (a), 2 (b), 3 (c) and 4 (d), and for cisplatin (e) inhibitor activity on cell viability of SAOS-2 cell line. The Log[concentration] in μM and the normalized response (%) of survival fraction of cells are reported on X and Y asses, respectively. For each complex, the curve interpolation with 50% survival cells is highlighted in Y dotted line and correspond to its $LogIC_{50}$. A comparison of all dose-response curves is reported (f).

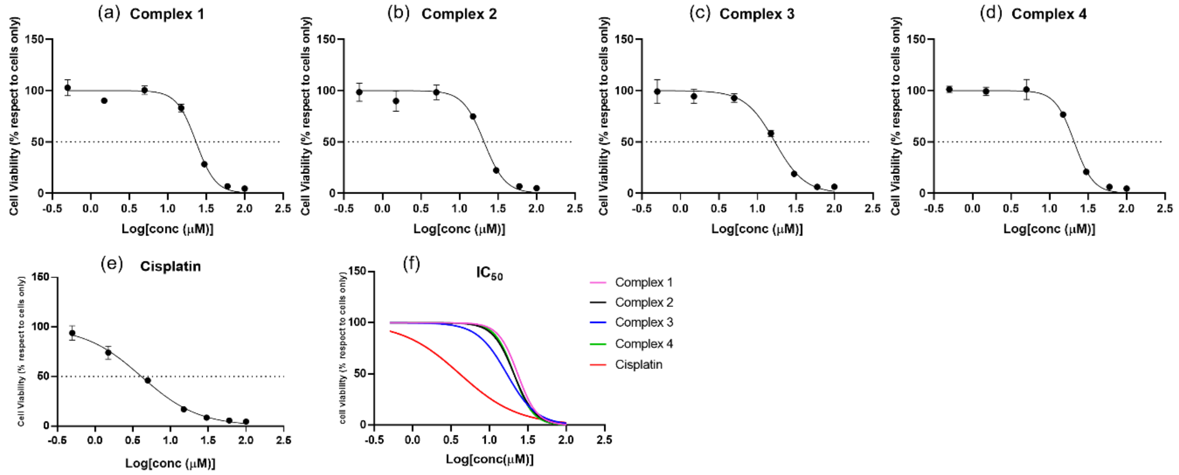

Figure S23: 2D *in vitro* screening of complexes 1-4 on hFOBs cell line by MTT assay and IC_{50} (μM) values. Dose-response curves used to generate IC_{50} (μM) for complexes 1 (a), 2 (b), 3 (c) and 4 (d), and for cisplatin (e) inhibitor activity on cell viability of hFOBs cell line. The Log[concentration] in μM and the normalized response (%) of survival fraction of cells are reported on X and Y asses, respectively. For each complex, the curve interpolation with 50% survival cells is highlighted in Y dotted line and correspond to its $LogIC_{50}$. A comparison of all dose-response curves is reported (f).

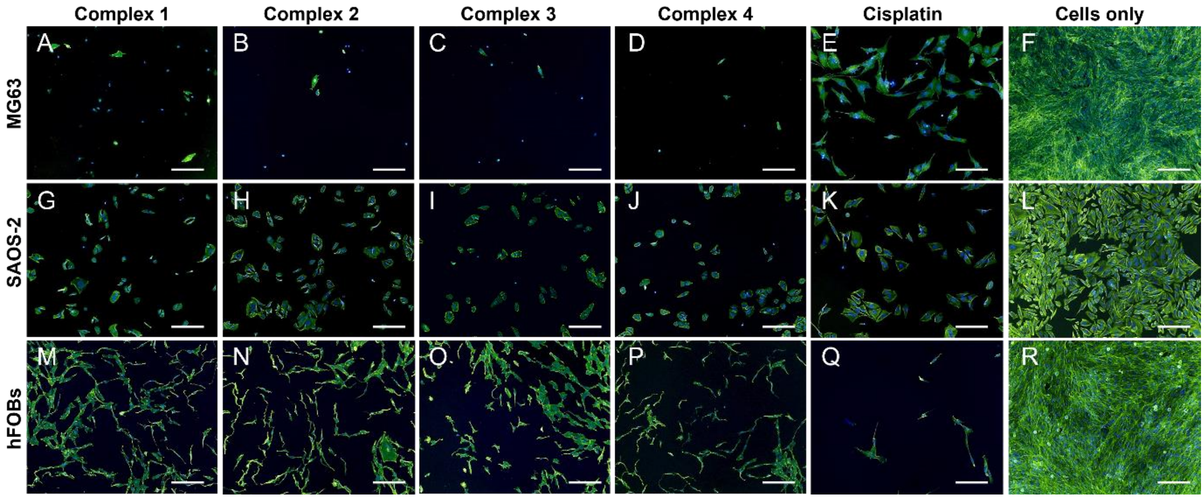

Figure S24: Actin and DAPI staining of 2D *in vitro* screening of complexes **1-4.** The cell morphology evaluation of MG63 (A – F), SAOS-2 (G – L) and hFOBs (M – R) cell lines cultured with complexes 1-4 and cisplatin are reported in the figure. For SAOS-2 and hFOBs 30 µM concentration was selected for the analysis, while 60 µM is reported for MG63 cells. F actin filaments in Phalloidin (Green) and cell nuclei in DAPI (Blue). Scale bars 200 µm.

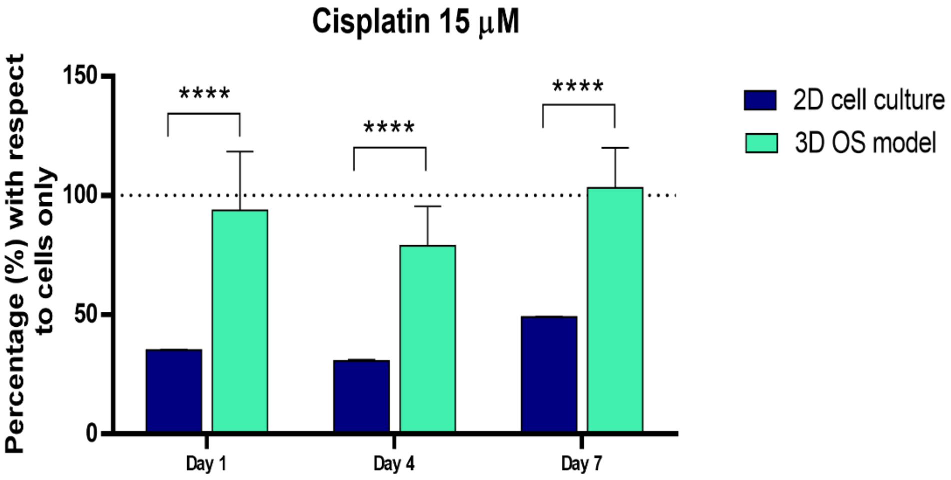

Figure S25: Anti-cancerous effect of cisplatin on 3D OS model *versus* 2D cell culture. Cell viability evaluation at day 1, 4 and 7 of culture by MTT assay. Data are reported in the graph as percentage (%) mean ± standard deviation. Significant differences of cisplatin cytotoxicity between different cell culture systems are reported in the graph at each time point ****p value ≤ 0.0001.

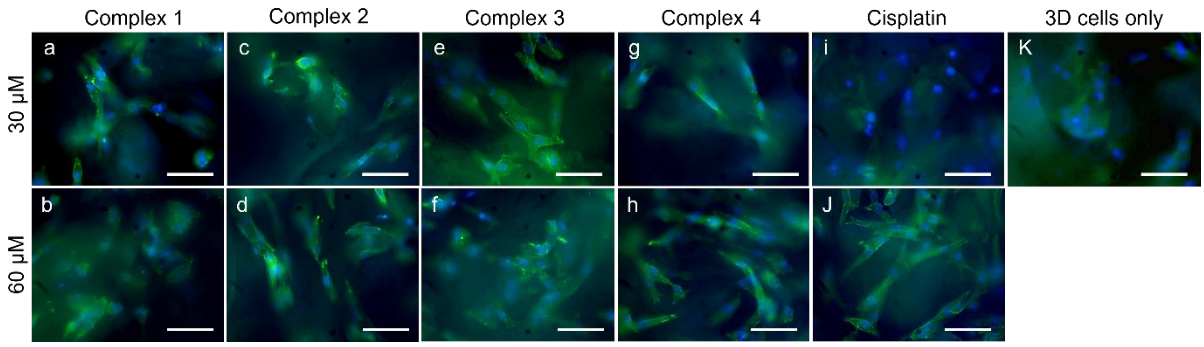

Figure S26: Actin and DAPI staining of *in vitro* 3D tumour-engineered models of osteosarcoma**.** Cell morphology evaluation of 3D tumour engineered models of MG63 cells after 72 hours in the presence of complex 1 (a;b), 2 (c;d), 3 (e;f), 4 (g;h) and cisplatin (i;j), and without any drug (k). F-Actin filaments in green (FITC) and cell nuclei in blue (DAPI). Scale bars 50 µm.
